# Development and Validation of an Arterial Pressure-Based Cardiac Output Algorithm Using a Convolutional Neural Network: Retrospective Study Based on Prospective Registry Data

**DOI:** 10.2196/24762

**Published:** 2021-08-16

**Authors:** Hyun-Lim Yang, Chul-Woo Jung, Seong Mi Yang, Min-Soo Kim, Sungho Shim, Kook Hyun Lee, Hyung-Chul Lee

**Affiliations:** 1 Department of Anesthesiology and Pain Medicine Seoul National University Hospital Seoul Republic of Korea; 2 Biomedical Research Institute Seoul National University Hospital Seoul Republic of Korea; 3 Department of Anesthesiology and Pain Medicine Seoul National University College of Medicine Seoul Republic of Korea; 4 School of Computing Korea Advanced Institute of Science and Technology Daejeon Republic of Korea; 5 Department of Information and Communication Engineering Daegu Gyeongbuk Institute of Science & Technology (DGIST) Daegu Republic of Korea

**Keywords:** cardiac output, deep learning, arterial pressure

## Abstract

**Background:**

Arterial pressure-based cardiac output (APCO) is a less invasive method for estimating cardiac output without concerns about complications from the pulmonary artery catheter (PAC). However, inaccuracies of currently available APCO devices have been reported. Improvements to the algorithm by researchers are impossible, as only a subset of the algorithm has been released.

**Objective:**

In this study, an open-source algorithm was developed and validated using a convolutional neural network and a transfer learning technique.

**Methods:**

A retrospective study was performed using data from a prospective cohort registry of intraoperative bio-signal data from a university hospital. The convolutional neural network model was trained using the arterial pressure waveform as input and the stroke volume (SV) value as the output. The model parameters were pretrained using the SV values from a commercial APCO device (Vigileo or EV1000 with the FloTrac algorithm) and adjusted with a transfer learning technique using SV values from the PAC. The performance of the model was evaluated using absolute error for the PAC on the testing dataset from separate periods. Finally, we compared the performance of the deep learning model and the FloTrac with the SV values from the PAC.

**Results:**

A total of 2057 surgical cases (1958 training and 99 testing cases) were used in the registry. In the deep learning model, the absolute errors of SV were 14.5 (SD 13.4) mL (10.2 [SD 8.4] mL in cardiac surgery and 17.4 [SD 15.3] mL in liver transplantation). Compared with FloTrac, the absolute errors of the deep learning model were significantly smaller (16.5 [SD 15.4] and 18.3 [SD 15.1], *P*<.001).

**Conclusions:**

The deep learning–based APCO algorithm showed better performance than the commercial APCO device. Further improvement of the algorithm developed in this study may be helpful for estimating cardiac output accurately in clinical practice and optimizing high-risk patient care.

## Introduction

Cardiac output (CO; L/min), the amount of blood pumped from the left ventricle per minute, is the main determinant of oxygen delivery to the body, including to the brain and vital organs, and is an important monitoring parameter during hemodynamic optimization. It is sometimes referred to as the stroke volume (SV; mL/beat), calculated by dividing the CO by the heart rate (HR; beats per minute). Particularly, in the perioperative phase, hemodynamic optimization is directly related to postoperative complications, which are the third leading cause of death worldwide [1]. Patients’ outcomes can potentially be improved by applying immediate treatment to maintain CO within 4-8 L/min or maintain SV within 60-100 mL/beat during major surgery [2]. Optimization of CO is also essential for high-risk patients [3]. Early interventions for hemodynamic control can significantly reduce mortality by more than 20% in high-risk patients [4].

The thermodilution method using a pulmonary artery catheter (PAC) has been regarded as a gold standard for measuring CO in clinical practice [5]. However, owing to its invasiveness, the risks associated with placement limit its use to only cardiac surgery, liver transplantations, and some critically ill patients. Instead, arterial pressure-based cardiac output (APCO) methods have been proposed as a less invasive method for estimating CO from the arterial pressure waveform without the risk of complications associated with a PAC [6,7]. These methods estimate systemic vascular resistance from arterial pressure waveform and general patient characteristics and predict the SV. As the arterial line is less invasive and usually inserted for continuous blood pressure monitoring, APCO devices such as the FloTrac (Edwards Lifesciences, Irvine, CA, United States) or LiDCO Rapid (LiDCO Ltd, London, UK) are widely used in perioperative CO management. However, inaccuracies of the commercially available APCO devices have been reported, especially, in sepsis or liver transplantation patients [8,9]. Improvements of the algorithm by researchers are also not possible, because only a subset of the algorithm has been openly released.

Recent advances in machine learning techniques have led to many new approaches to solving clinical problems [10]. Deep learning techniques, such as convolutional neural networks (CNNs), have performed well in bio-signal analysis [11]. In contrast, the shortage of clinical bio-signal data makes it difficult to train deep learning models properly [12,13]. Publicly available bio-signal datasets are still limited compared with medical imaging or structured datasets [14-16]. Furthermore, data with reduced clinical use, such as PAC-based CO, worsen this tendency. In this case, after training a model with a relatively common dataset, a transfer learning technique can be used to refine the model parameters with relatively rare data. Previous studies also reported performance gains from transfer learning with bio-signal data [17-20].

In this study, a novel APCO algorithm was built using a transfer learning technique. The algorithm learned the commercial APCO algorithm and then was trained with less-common PAC data. In addition, several preprocessing techniques were proposed to analyze the arterial pressure waveforms for predicting CO. Finally, the deep learning model was validated using real-world bio-signal data, which was collected during a separate period than the training data and includes cardiac surgery and liver transplantation patients. This study hypothesized that a model developed using real-world clinical data, deep learning techniques, and transfer learning techniques can be more accurate than a commercial APCO device for estimating CO. 

## Methods

### Study Approval

All data used in this study were obtained from the prospective registry of the vital signs for surgical patients at Seoul National University Hospital. The registry was approved by the Institutional Review Board of Seoul National University Hospital (H-1408-101-605) and registered at the clinical trial registration site (ClinicalTrials.gov, NCT02914444). This retrospective study was also approved by the Institutional Review Board (H-2007-015-1138). However, the need for written informed consent was waived because of the anonymity of the data.

### Data Collection

The registry collected synchronous vital signs and bio-signal data from various medical devices using the Vital Recorder Program [21]. Among the cases in the registry, those from between August 2016 and September 2019 were used in this study. The cases collected in the last 8 months of the study period (February 2019 to September 2019) were used for the testing dataset. The remaining cases were used for training the model.

During data collection, CO monitors were used according to the discretion of the anesthesiologist. CO values were collected at 2-second intervals from the serial port of APCO devices, such as the EV1000 clinical platform or the Vigileo system (Edwards Lifesciences, Irvine, CA, United States) with a fourth-generation FloTrac algorithm or a PAC-based device such as the Vigilance II (Edwards Lifesciences, Irvine, CA, United States). The arterial pressure waveform was recorded at 500 Hz from the analog output port of the TRAM module (GE Healthcare, Chicago, IL, United States), and the heart rate was recorded at 2-second intervals from the serial port of the Solar 8000 patient monitor (GE Healthcare, Chicago, IL, United States). General patient characteristics (ie, age, sex, weight, and height) were collected from electronic medical records.

The deep learning model requires massive amounts of data for good performance. However, with PAC-based CO monitoring data, such as the Vigilance II, it is difficult to retain massive amounts of data, which hinders the ability to develop of a good deep learning model using real-world databases. Hence, the model was pretrained using APCO data from the EV1000 or Vigileo, from which data are relatively easy to obtain. After that, we tuned the model using PAC data from the Vigilance II, from which data are hard to obtain. In total, 1572 cases of surgery were recorded with APCO monitoring devices for pretraining, 290 cases were recorded with PAC-based CO monitoring devices for tuning or testing, and 195 cases were recorded with both APCO and PAC-based CO monitoring devices for tuning or testing. Among the 2057 cases, 1958 cases (95.19%) that were operated on from August 2016 to January 2019 were used for pretraining or tuning, and the remaining 99 cases (4.81%) that were operated on since February 2019 were used for testing.

### Data Preprocessing

A dataset of arterial pressure waveforms was preprocessed, including corresponding SV values. The arterial pressure waveforms were resampled from 500 Hz to 100 Hz and sliced into 20-second segments. Each pair of a 20-second segment and an SV was referred to as a “sample.” For preprocessing the samples, the following 4 steps were performed: (1) converting the output of PAC-based CO data to SV, (2) smoothing the APCO data, (3) delaying the PAC data, and (4) removing unsuitable samples.

The APCO monitoring device provides the SV value, because it estimates the amount of blood moving from arterial waves when a stroke occurs. In contrast, a PAC-based CO monitoring device emits the CO value, because it measures the temperature change by blood flow from the pulmonary artery catheter. Owing to the physiological differences between the 2 methods, CO values needed to be converted from the Vigilance II monitor to SV, using HR values (SV = CO/HR), to synchronize with APCO data.

In addition, because there were larger fluctuations in the APCO data than clinically expected CO changes, the APCO data were smoothed using a locally weighted scatterplot smoother (LOWESS) algorithm [22]. We used the hyperparameter, λ=0.03, of the LOWESS algorithm. If APCO data were not recorded for more than 200 seconds in a single case due to recording errors, the LOWESS algorithm was applied separately.

In addition, the PAC-based SV value was delayed, to synchronize the time differences between arterial pressure waveforms and PAC data. There are several minutes of delay in the CO values using the Vigilance II in its “Trend” mode [23,24]. To determine the time lag of the CO values in the Trend modes, we used the device with the “STAT” mode in several cases, allowing both the CO values and the “CO stat” values to be transferred. Then, the CO values were compared with the CO stat values, and we obtained the minimum mean absolute difference with the delay of 2 minutes. Thus, PAC-based SV values were shifted to the time 2 minutes earlier than the recorded time. More detailed descriptions and examples of delaying PAC values are provided in Multimedia Appendix 1.

Finally, unsuitable samples were removed for robust deep learning model training. Samples with a blood pressure <25 mmHg or >250 mmHg and an SV <20 mL or >200 mL were removed from the dataset. After applying a beat-detection algorithm, we eliminated samples with an HR <30 beats/min or >180 beats/min, a pulse pressure <20 mm Hg, or with frequent (>50%) ventricular premature beats [25].

### Model Building

**Figure 1:**
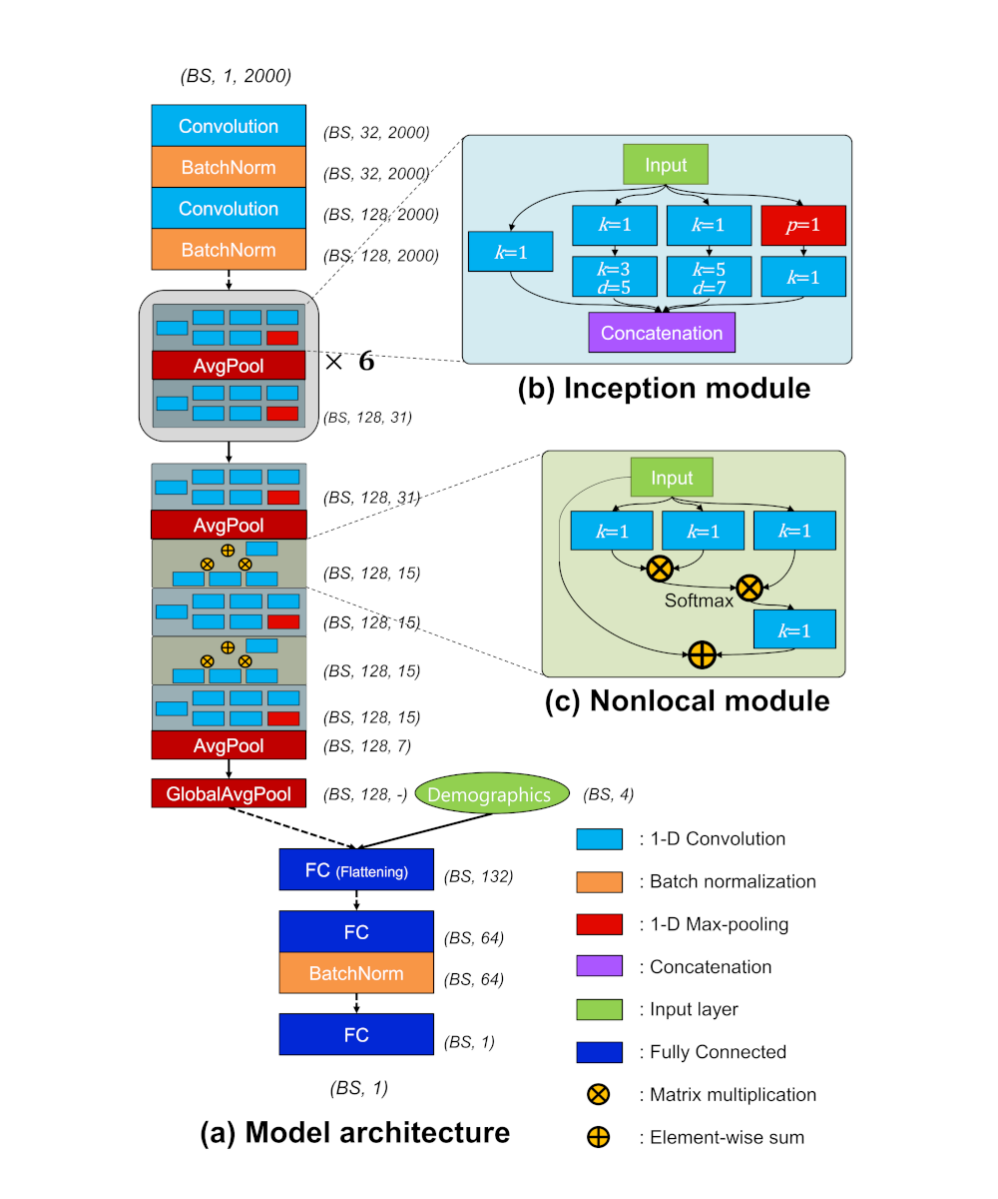
Proposed convolutional neural network model for estimating stroke volume from arterial pressure waveform. (A) Overall model architecture. (B) Details of the inception module. (C) Details of the nonlocal module. The variable *k* indicates kernel size of the convolution layer, *d* means the dilation rate of the convolution layer, and *p* represents pooling rate. GlobalAvgPool indicates the global average pooling layer, which computes the mean value for each feature map and supplies abstracted feature maps to the flattening layer. Dotted arrows represent dropout of 0.5, while solid arrows mean full connections. BS: batch size; FC: fully connected.

A CNN model was designed to learn the appropriate feature extraction from the 20-second segments of arterial pressure waveforms. The goal of the model was to estimate PAC-based CO values using the arterial pressure waveform and the patient’s demographics (ie, age, sex, weight, and height) (Figure 1). The model consists of 2 parts: feature extraction and regression. The feature extraction part of the model was composed of 2 successive pairs of convolution and batch-normalization layers, 15 inception modules, 2 nonlocal modules with pooling, and dropout layers [26-29]. The regression part of the model, which was composed of 3 fully connected, batch-normalization, and dropout layers, takes a concatenation of the extracted features from the feature extraction part and the patient’s demographic information as input and returns a predicted SV value. For all layers, a rectified linear unit was used as the activation function.

Input samples were abstracted by 2 consecutive pairs of convolution and batch-normalization layers (dropout rate of 0.5), before feeding them into the inception modules. The inception modules consisted of 4 paths with 6 convolution layers and 1 pooling layer. The filter size of each path in the inception module was 32, and the outputs of the 4 paths were concatenated to 128. The detailed configurations of the inception module are illustrated in Figure 1B. Note that *p* represents the pooling rate of the average pooling layer, *k* indicates the kernel size of the convolution layer, and *d* is the dilation rate of the dilated convolution layer. After an odd-numbered inception module, an average pooling layer intervened to reduce the size of feature maps from the previous inception module. The shaded inception module block in Figure 1A was repeated 6 times. Nonlocal modules were added just before the last 2 inception modules to consider the global covariance in each segment. The first fully connected layer was a flattening layer (size of 132) that took the concatenation of the average of feature maps by global average pooling (size of 128) and demographic information (size of 4). The number of neurons in the last 2 fully connected layers was 64 and 1, respectively. Immediately after the second fully connected layers, there was a batch-normalization layer to achieve robustness for cases in which a batch is biased to a specific SV range. The last fully connected layer consisted of a single neuron, which represents a float value of the SV. For all fully connected layers, a dropout of 0.5 was used. The output dimensions of each module or layer are described in Figure 1. Note that dimensions are represented as *BS*, channel, and length, where *BS* indicates batch size. The dotted arrows indicate a dropout of 0.5.

### Model Training

Our model was trained using a transfer learning method with 2 steps: pretraining and tuning. During pretraining, SV values were used from EV1000 or Vigileo to find rough parameters of the model for analyzing arterial pressure waveforms. The input of pretraining was 20-second segments of arterial pressure waveform and patient demographic information, and the output was the predicted SV. After that, the parameters were tuned using PAC data. The input and output of tuning were the same as in pretraining; however, the parameters of the regressor part were initialized with the Xavier algorithm to be retrained with PAC data [30]. Gradient-descent optimizers, RAdam, and Lookahead were used to update parameters [31,32]. The batch size was 512, and the loss function was the root mean squared error. Note that the loss function was calculated based on the equation (Ŷ = predicted value; Y = ground truth; *N* = batch size):

Root mean squared error = (∑*^N^*(Ŷ-Y)^2^/*N*)^1/2^** (1)**

The model was tested every 200 steps using 30% of the training datasets to calculate the validation errors. The training was stopped when the validation errors no longer decreased after 50 times and then was restarted with a smaller learning rate (decay rate of 0.5). The training was performed using our own program (code available at [33]), written in Python using PyTorch 1.1.0 on a graphics processing unit server with two 10-core Intel Xeon central processing units and eight Nvidia GTX 1080Ti graphics processing units.

### Statistical Analysis

The statistics of patient demographics were described in the training and testing groups, comparing the heterogeneity of the variables. Note that training groups contained pretraining and tuning datasets, and the testing group contained the testing dataset. For continuous variables (ie, age, height, and weight), a Mann-Whitney *U* test was performed for comparisons after testing for normality using a Shapiro-Wilk test. For categorical variables (ie, sex), a Pearson chi-square test was conducted for comparisons between groups.

The performance of the deep learning model was validated using error, absolute error, percentage error, and absolute percentage error, using the testing dataset. Each metric was calculated based on the equation (Ŷ = predicted value; Y = PAC value; *N* = number of samples):

Error (mL) = ∑*^N^* (Ŷ-Y)/*N*** (2)**

Absolute error (mL) = ∑*^N^* |Ŷ-Y|/*N*** (3)**

Percentage error (%) = [∑*^N^* {(Ŷ-Y)/Y}/*N*] × 100 **(4)**

Absolute percentage error (%) = {∑*^N^* |(Ŷ-Y)/Y|/*N*} × 100 **(5)**

Efforts were made to validate the generalizability and substitutability of our model by comparing its performance with the FloTrac in two radically different patient groups: the patients who underwent cardiac surgery and the patients who underwent liver transplantation surgery. Among the test dataset, both the FloTrac and Vigilance II devices were used simultaneously in 16 cases of cardiac surgery and 40 cases of liver transplantation surgery. With these 56 cases, direct comparisons were performed between the deep learning model and FloTrac using a paired *t* test for overall cases and each subgroup.

Spearman correlation coefficients were calculated between the SV of the deep learning model and PAC, and between the SV of the EV1000 or Vigileo and PAC. Bland-Altman analysis was used to test the agreement of either the pair of the deep learning model and PAC-based SV or the pair of the FloTrac and PAC-based SV [34]. Bias was defined as the mean difference between SVs, and the upper and lower limits of agreement were defined as ±1.96 SDs of the bias. The trending ability of the deep learning model was examined using a 4-quadrant plot analysis [35]. The concordance rate of the association for percentage changes in SV was calculated between our model or the FloTrac and PAC, with the exclusion of 10% of the changes [36].

All data are expressed as the mean (SD), median (interquartile range), or absolute numbers (%). *P* values <.05 were considered statistically significant. Statistical analyses were performed using Python Scipy 1.4.1.

## Results

Data from 2057 surgical cases (1232 general, 59.89%; 636 thoracic, 30.92%; 159 urologic, 7.73%; 23 gynecologic, 1.12%; 6 otolaryngologic, 0.29%; and 1 plastic surgery, 0.05%) in the registry were extracted and preprocessed. Of these 2057 surgical cases, we used the data from 1958 cases (95.19%) for training and 99 cases (4.81%; 59 cardiac surgery, 60%; 40 liver transplantation, 40%) for testing. For transfer learning, of 2057 surgical cases, we used the data from 1572 cases (76.42%) for pretraining and the data from 386 cases (18.77%; 245 cardiac surgeries, 63.5%; 141 liver transplantations, 36.5%) for tuning. Demographic information of the patients was not different between the training and testing datasets, except, that the patients in the testing dataset were slightly older (Table 1).

**Table 1:** Demographics of patients for the training and testing dataset.

Characteristic	Training dataset (n=1958)	Testing dataset (n=99)	Statistical test	
			Method used	*P* value
Age, median (interquartile range), years	61.2 (51.2-69.5)	63.8 (57.4-71.9)	Mann-Whitney *U* test	.02
Sex, number male (%)	1195 (61.03%)	66 (67%)	Pearson chi-square test	.309
Height, median (interquartile range), years	164.0 (157.3-170.0)	163.4 (156.7-168.0)	Mann-Whitney *U* test	.231
Weight, median (interquartile range), kg	63.1 (55.2-72.4)	64.0 (57.5-72.1)	Mann-Whitney *U* test	.494

The absolute error of the deep learning model for the testing dataset was 14.5 (SD 13.4) mL (Table 2). In the subgroup analysis, the absolute errors of the deep learning model were 10.2 (SD 8.4) mL for cardiac surgery and 17.4 (SD 15.3) for liver transplantation.

**Table 2:** Stroke volume estimation of the deep learning model.

Measure	Overall (n=99), mean (SD)	Cardiac surgery (n=59), mean (SD)	Liver transplantation (n=40), mean (SD)
Error (mL)	–4.4 (19.2)	2.3 (13.0)	–9.0 (21.3)
Absolute error (mL)	14.5 (13.4)	10.2 (8.4)	17.4 (15.3)
Percentage error (%)	0.4 (27.4)	9.0 (26.8)	–5.5 (26.2)
Absolute percentage error (%)	20.5 (18.2)	20.5 (19.4)	20.4 (17.4)

In the testing dataset, the data from 56 cases with both PAC and FloTrac (16 cardiac surgery, 29%; 40 liver transplantation, 71%) data were used to compare the performance of the deep learning model with that of the FloTrac. The absolute error of the deep learning model was significantly lower than that of the FloTrac (16.5 mL vs 18.3 mL, *P*<.001; Table 3). In the subgroup analysis, the absolute errors of the deep learning model were lower than those of the FloTrac, in both cardiac surgery (11.1 mL vs 14.3 mL, *P*<.001) and liver transplantation (17.4 mL vs 19.0 mL, *P*<.001; Table 3). The individual plots of the time course of the measured and predicted SVs in all 99 testing cases are available in Multimedia Appendix 2.

**Table 3:** Comparison of performance in stroke volume estimation between the deep learning model and FloTrac algorithm.

Measure		Deep learning model, mean (SD)	FloTrac, mean (SD)	Statistical test	
				Count, n	*P* value
**Error (mL)**					
	Overall (n=56)	–7.9 (20.7)	–8.4 (22.1)	158725	<.001
	Cardiac surgery (n=16)	–1.7 (15.2)	2.7 (18.3)	65260	<.001
	Liver transplantation (n=40)	–9.0 (21.3)	–10.3 (22.2)	93465	<.001
**Absolute error (mL)**					
	Overall (n=56)	16.5 (14.8)	18.3 (15.1)	158725	<.001
	Cardiac surgery (n=16)	11.1 (10.5)	14.3 (11.8)	65260	<.001
	Liver transplantation (n=40)	17.4 (15.3)	19.0 (15.4)	93465	<.001
**Percentage error (%)**					
	Overall (n=56)	–4.4 (26.9)	–5.6 (28.6)	158725	<.001
	Cardiac surgery (n=16)	1.8 (29.9)	9.5 (34.9)	65260	<.001
	Liver transplantation (n=40)	–5.5 (26.2)	–8.3 (26.5)	93465	<.001
**Absolute percentage error (%)**					
	Overall (n=56)	20.3 (18.3)	22.5 (18.5)	158725	<.001
	Cardiac surgery (n=16)	19.3 (22.9)	25.4 (25.7)	65260	<.001
	Liver transplantation (n=40)	20.4 (17.4)	22.0 (16.9)	93465	<.001

**Figure 2:**
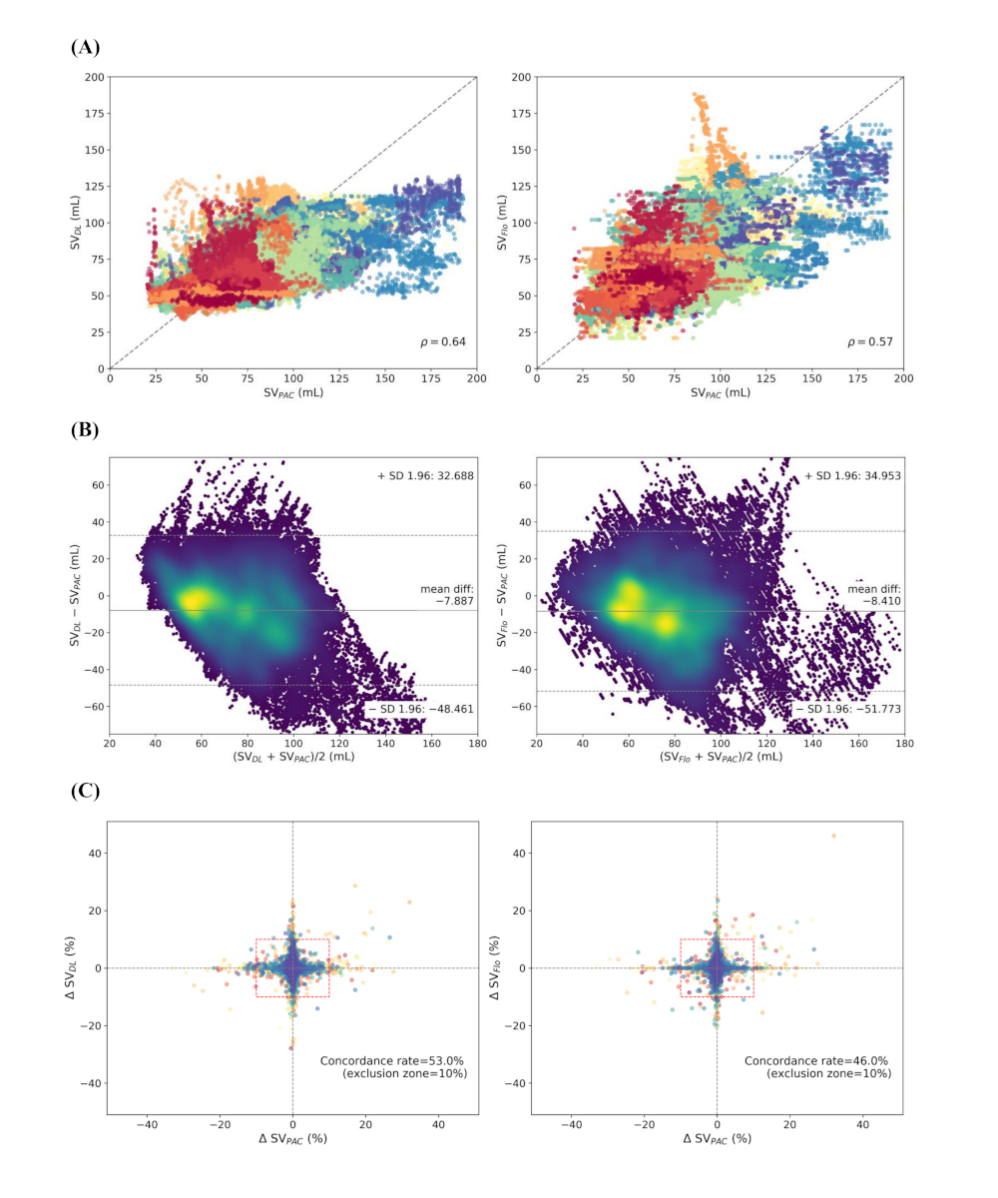
Scatter plot. (A) Bland-Altman plot with density highlight. (B) Four-quadrant plots. (C) Plot between target stroke volume and predicted stroke volume. DL: deep learning; Flo: FloTrac; PAC: pulmonary artery catheter; SV: stroke volume.

The Spearman rho value of our deep learning model was 0.64 (*P*<.001), whereas the rho value of the FloTrac was 0.57 (*P*<.001; Figure 2). Bland-Altman analysis demonstrated that the lower and upper limits of agreements, respectively, were –48.5 (95% CI –48.7 to –48.3) mL and 32.7 (95% CI 32.5-33.0) mL for the deep learning model and –51.8 (95% CI –52.0 to –51.5) mL and 35.0 (95% CI 34.8-35.3) mL for the FloTrac. The 4-quadrant plot showed concordance rates of 53% for the deep learning model and 46% for FloTrac (Figure 2). Mean differences were smaller in our deep learning model than in the commercial APCO device.

## Discussion

### Principal Results

In this study, we built and evaluated an open-source deep learning–based APCO algorithm using a large set of prospectively collected registry data. The performance of the deep learning model was better than that of the FloTrac, the commercially available APCO algorithm.

A mathematical analysis of the association between arterial blood flow and pressure waveform has a long history of over 100 years [37]. A key factor in this flow-pressure association is the estimation of systemic vascular resistance (SVR), because the flow is determined by the pressure gradient and vascular resistance [38]. However, since SVR changes with the patient's condition, the coefficient also needs to be updated in real-time [39-41]. An uncalibrated APCO algorithm, which automatically updates the coefficient using the patient’s general characteristics and arterial pressure waveforms, can be a convenient solution in clinical situations. However, currently available commercial uncalibrated APCO devices have been reported to work poorly in patients with vasodilatory states such as sepsis or liver transplantation [8,9,38,42,43]. Our results showed that our deep learning–based APCO algorithm outperformed the FloTrac algorithm in both cardiac surgery and liver transplantation patients. However, both the deep learning–based and FloTrac algorithms showed a positive bias in cardiac surgery patients, who usually have low SVs, and a negative bias in liver transplantation patients, who usually have high SVs. This tendency to return to the average is also shown in Figure 2C and may be a fundamental limitation of the APCO algorithm, in which SVR should be estimated only from the arterial pressure waveform and patient’s demographics. Otherwise, this tendency may occur because most of the data obtained from routine clinical practice are within the normal SV range.

### Clinical, Academic, and Technical Implications

The measurement of CO is essential for clinical hemodynamic optimization. As the deep learning model is more accurate than the commercial APCO device, it can help enhance patient management and improve final outcomes. For example, goal-directed fluid therapy or SV optimization can be performed using our model. However, further validation and implementation are required for clinical applications. Disclosing our dataset and model, researchers can improve the model and validate our algorithm or their own algorithm. We believe that this approach can facilitate developing more accurate APCO algorithms and help in its clinical application. In the domain of detecting arrhythmia in the electrocardiogram, numerous studies and technologies have been proposed using publicly available data, such as the MIT-BIH dataset [44-47]. Likewise, our open dataset can be an academic reference for the APCO domain. Finally, a transfer learning method was proposed based on 2 datasets with different characteristics in bio-signal fields, and its scalability was confirmed. These techniques will provide good technical strategies for developing machine learning algorithms in the medical field with scanty data, such as PAC-based CO.

### Comparison With Prior Work

In a previous study, Moon et al [48] built a deep learning–based APCO algorithm using the data of 31 liver transplantation patients. However, their model only included the patients who underwent liver transplantation and has not been validated in the other types of surgery. In this study, a larger dataset was used that included both cardiac surgery and liver transplantation cases, with balanced ratios. In addition, a transfer learning technique was used, in which the parameters were pretrained with a large amount of APCO data and then tuned with PAC data. This may explain why our model worked better than the FloTrac for both cardiac and liver transplantation cases.

### Limitations

This study has some limitations. First, the data used in this study were from a single-center registry of a surgical cohort, which may contain a limited range of CO. This problem can be overcome by adding more data. However, the clinical use of PAC is gradually decreasing; other modalities such as a Doppler flowmeter or echocardiogram are required. Second, continuous CO measurement methods used as ground-truth values in this study can be less accurate in certain situations, such as in rapid fluid administration, compared with the gold-standard intermittent thermodilution technique [49]. In addition, the delay time for processing in the Vigilance II monitor was not fully revealed [24]. Third, there was a statistical difference in age between the training and testing sets. This was an inevitable problem that occurred because of the use of real-world clinical data based on a prospective registry. However, elderly patients may have isolated systolic hypertension, which may alter the arterial pressure waveforms and affect the results. Fourth, there was no visualization with explainable artificial intelligence algorithms of how the proposed algorithm produces the results [50,51]. A proposal for a method that can display an indication for high or low CO and SVR from a waveform would have great clinical benefit. Finally, although various technological methods have been adopted, our developed model may be a local optimum and not a global optimum. Therefore, the raw data of this study was disclosed, allowing other researchers to improve the model.

### Conclusions

In conclusion, an uncalibrated APCO algorithm was developed and validated using a CNN and a transfer learning technique. The performance of our model was better than that of current commercial, uncalibrated APCO devices. Further improvement of the open-source algorithm developed in this study may be helpful for estimating cardiac output accurately in clinical practice and optimizing high-risk patient care.

## Appendix

Multimedia Appendix 1 Details of delaying the pulmonary artery catheter cardiac output data.

Multimedia Appendix 2 The individual plots of the time course of the measured and predicted stroke volume in all 99 testing cases.

## Abbreviations

APCO: arterial pressure-based cardiac output
CNN: convolutional neural network
CO: cardiac output
HR: heart rate
LOWESS: locally weighted scatterplot smoother
PAC: pulmonary artery catheter
SV: stroke volume
SVR: systemic vascular resistance
