# Supplementary material for: Development and Validation of an Arterial Pressure-Based Cardiac Output Algorithm Using a Convolutional Neural Network: Retrospective Study Based on Prospective Registry Data
Source: JMIR Med Inform. 2021 Aug 16;9(8):e24762. doi: 10.2196/24762 (PMC8406105; doi:10.2196/24762)
Supplement: Multimedia Appendix 1 [file medinform_v9i8e24762_app1.docx]

# Supplemental Materials for the development and validation of an arterial pressure-based cardiac output algorithm using a convolutional neural network: A retrospective study based on prospective registry data

### Details of delaying the PAC data

The PAC-based CO monitoring device measures the temperature changes of saline affected by blood flow from the pulmonary artery catheter, which is inserted from the peripheral vein to the pulmonary artery. They generate continuous CO values with an average of multiple measurements to maintain the conventional legacy, compared with bolus or standard pulsatile techniques.[^1–3^](https://www.zotero.org/google-docs/?zEQMYV) Therefore, we had to consider the actual measurements (not averaged values) as input for a deep learning model to build a real-time APCO algorithm. The Vigilance II (Edwards Lifesciences, Irvine, CA, USA), a PAC-based device for our model building, provides user options of ‘Trend_mode’ and ‘STAT_mode’ (a.k.a CCO mode and ICO mode), which provides averaged and actual measurements, respectively. A couple of studies reported the time lag between the Trend and STAT modes; hence, we experimentally determined the best time delay of the Trend mode from our dataset.[^4,5^](https://www.zotero.org/google-docs/?lpmKVP)

Supplementary figure 1 shows the mean absolute error (MAE) between the Trend mode and n-sec delayed STAT mode per case. Note that the unit of the x-axis was 2 s, which was the output interval of the Vigilance II. In our dataset, the best matched time delay of the Trend mode was 120 s with an MAE of 0.0421. Therefore, we prepared a 20-s segment of the arterial waveform to be 2 min earlier than the current CO value.


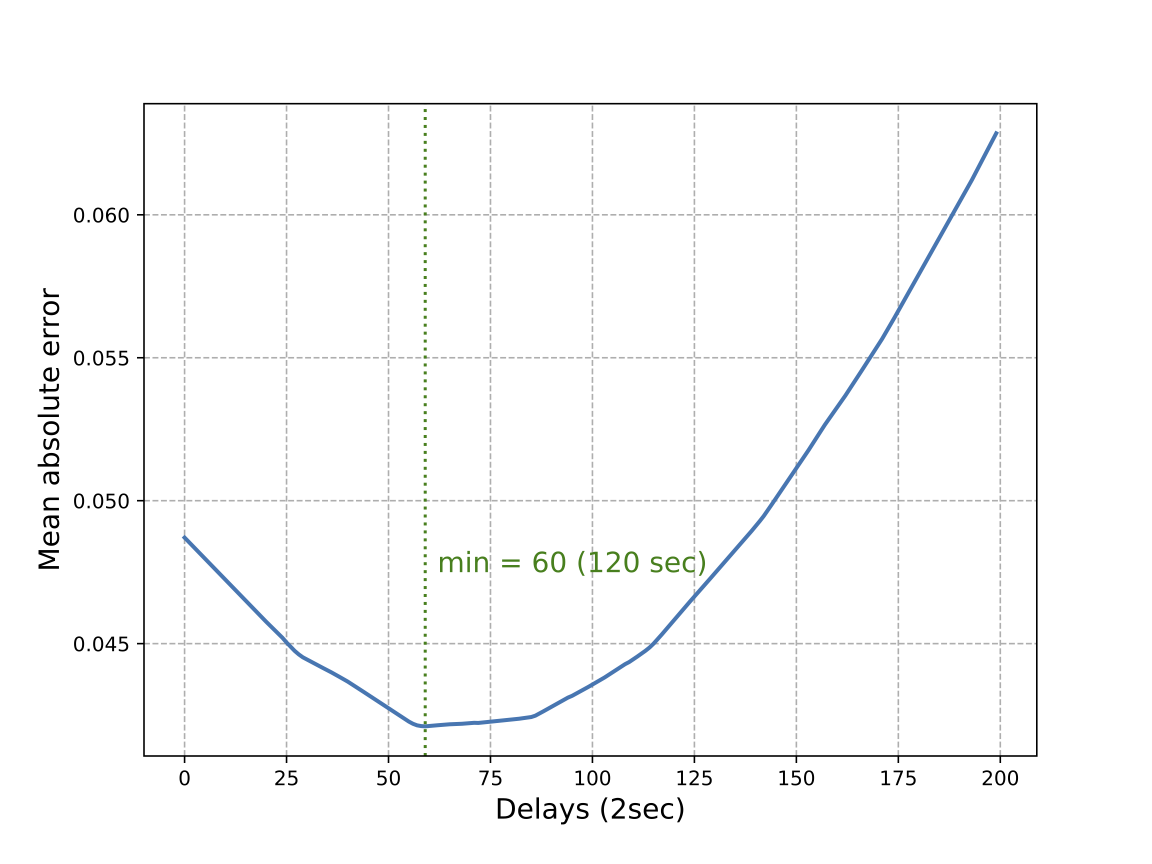


**Supplementary figure 1.** Mean absolute error per delays. Delay of 120 seconds showed the lowest mean absolute error.

Supplementary Figures 2 and 3 depict two-minute delayed PAC examples of two surgeries: cardiac surgery and liver transplantation surgery. Note that the blue line indicates the Trend mode and the orange line represents the STAT mode. With two minutes of delay of the STAT mode value, CO values had greater agreement.


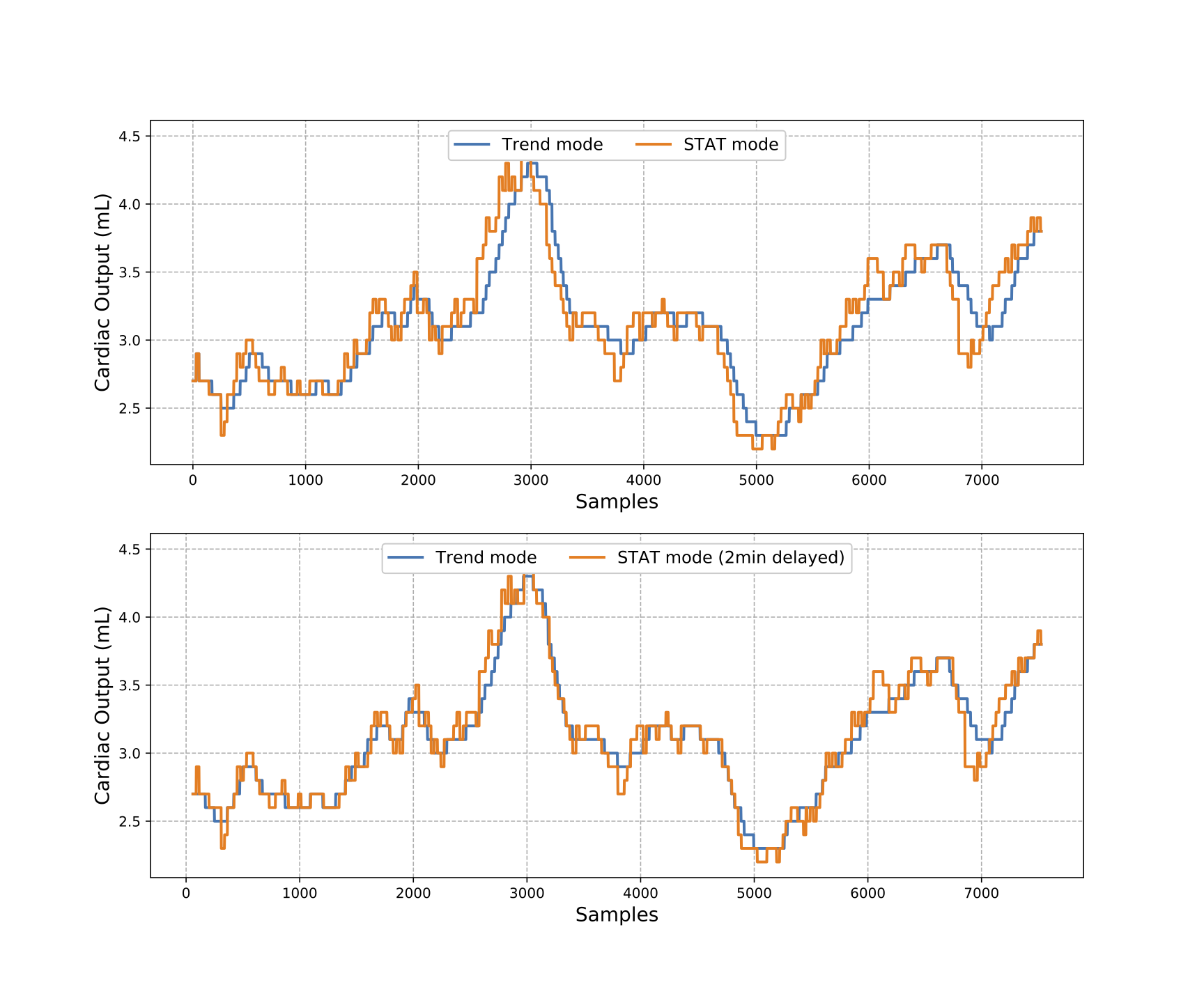


**Supplementary figure 2.** Delayed PAC example of cardiac surgery. Top: CO without delay. Bottom: CO with two-minute delay.


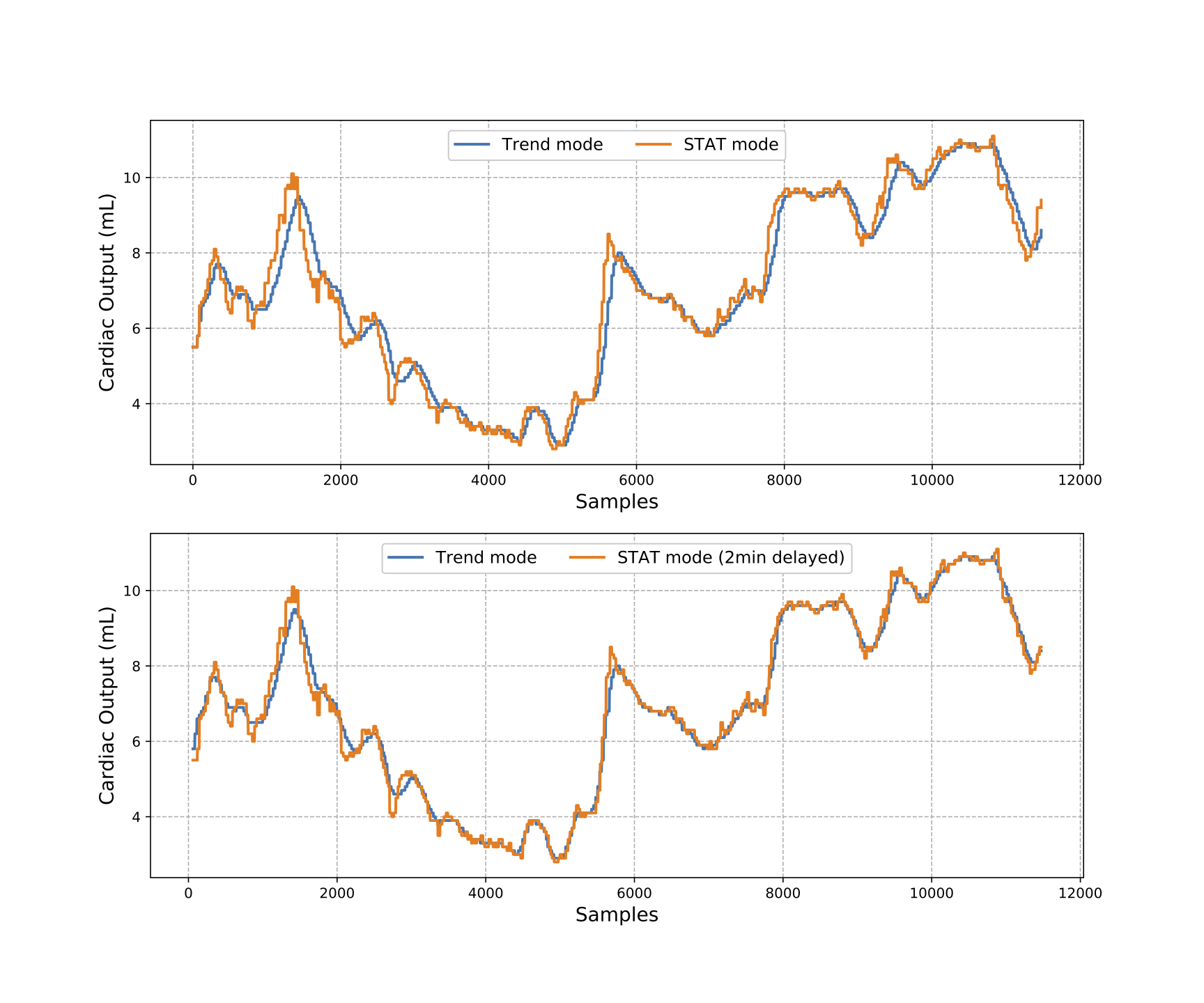


**Supplementary figure 3.** Delayed PAC example of liver transplantation surgery. Top: CO without delay. Bottom: CO with two-minute delay.

**References**

[1. Lefrant JY, Bruelle P, Ripart J, et al. Cardiac output measurement in critically ill patients: comparison of continuous and conventional thermodilution techniques. *Can J Anaesth J Can Anesth*. 1995;42(11):972-976. doi:10.1007/BF03011067](https://www.zotero.org/google-docs/?6f0pI5)

[2. Boldt J, Menges T, Wollbrück M, Hammermann H, Hempelmann G. Is continuous cardiac output measurement using thermodilution reliable in the critically ill patient? *Crit Care Med*. 1994;22(12):1913-1918.](https://www.zotero.org/google-docs/?6f0pI5)

[3. Yelderman ML, Ramsay MA, Quinn MD, Paulsen AW, McKown RC, Gillman PH. Continuous thermodilution cardiac output measurement in intensive care unit patients. *J Cardiothorac Vasc Anesth*. 1992;6(3):270-274. doi:10.1016/1053-0770(92)90137-v](https://www.zotero.org/google-docs/?6f0pI5)

[4. Lazor MA, Pierce ET, Stanley GD, Cass JL, Halpern EF, Bode RH. Evaluation of the accuracy and response time of stat-mode continuous cardiac output. *J Cardiothorac Vasc Anesth*. 1997;11(4):432-436. doi:10.1016/S1053-0770(97)90050-1](https://www.zotero.org/google-docs/?6f0pI5)

[5. Lakhal K, Ehrmann S, Boulain T. Predictive performance of passive leg raising in patients with atrial fibrillation. *Br J Anaesth*. 2016;117(3):399. doi:10.1093/bja/aew233](https://www.zotero.org/google-docs/?6f0pI5)
