# Supplementary material for: Development and Validation of an Arterial Pressure-Based Cardiac Output Algorithm Using a Convolutional Neural Network: Retrospective Study Based on Prospective Registry Data
Source: JMIR Med Inform. 2021 Aug 16;9(8):e24762. doi: 10.2196/24762 (PMC8406105; doi:10.2196/24762)
Supplement: Multimedia Appendix 2 [file medinform_v9i8e24762_app2.pdf]

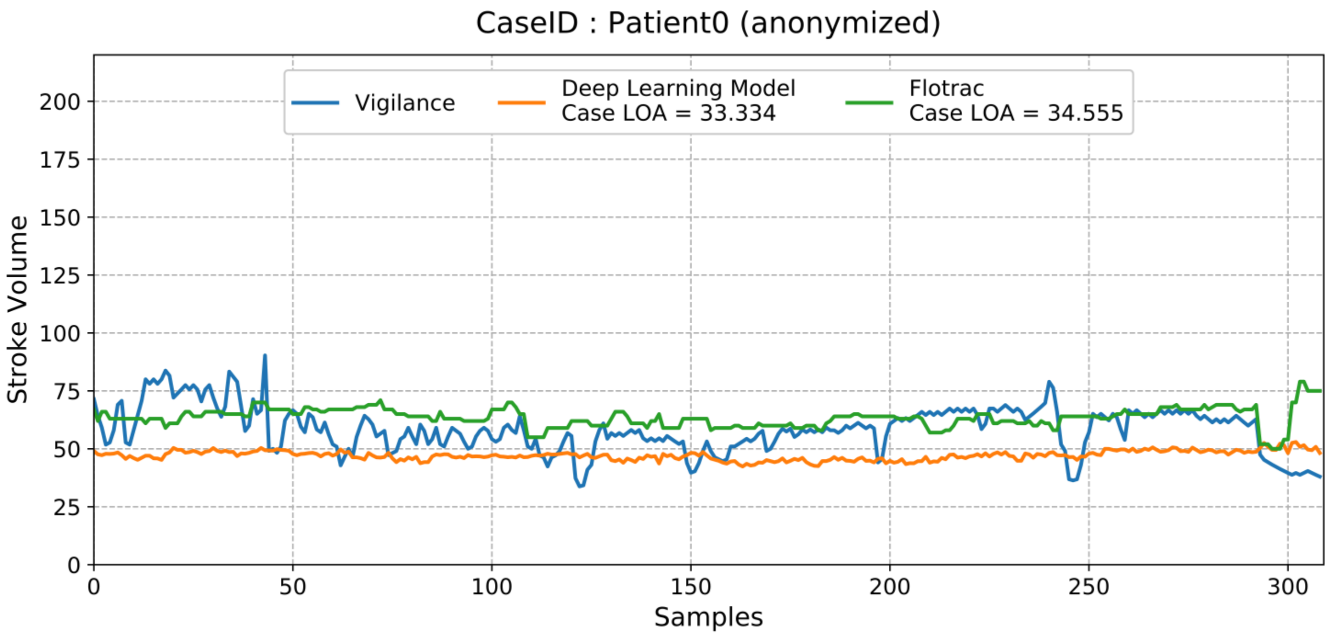
CaseID : Patient0 (anonymized)
Vigilance
Deep Learning Model
Case LOA = 33.334
Flotrac
Case LOA = 34.555
Stroke Volume
Samples
0
25
50
75
100
125
150
175
200
0
50
100
150
200
250
300

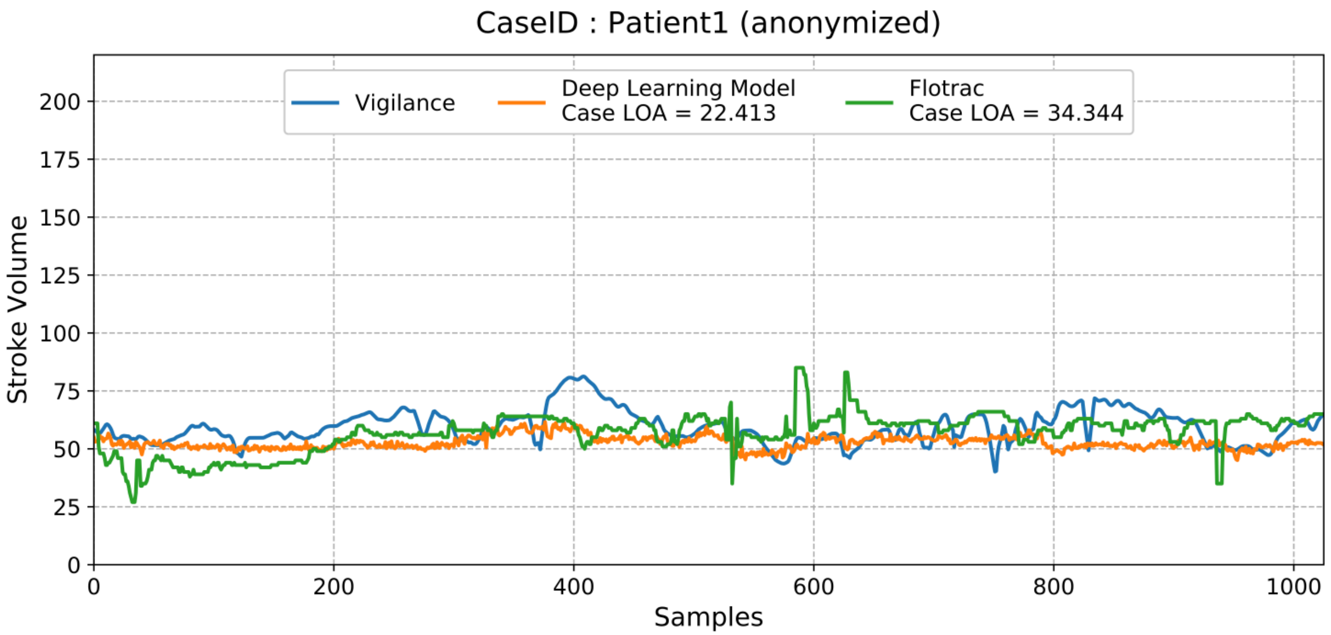

CaseID : Patient1 (anonymized)
Vigilance
Deep Learning Model
Case LOA = 22.413
Flotrac
Case LOA = 34.344
Stroke Volume
Samples
0
25
50
75
100
125
150
175
200
0
200
400
600
800
1000

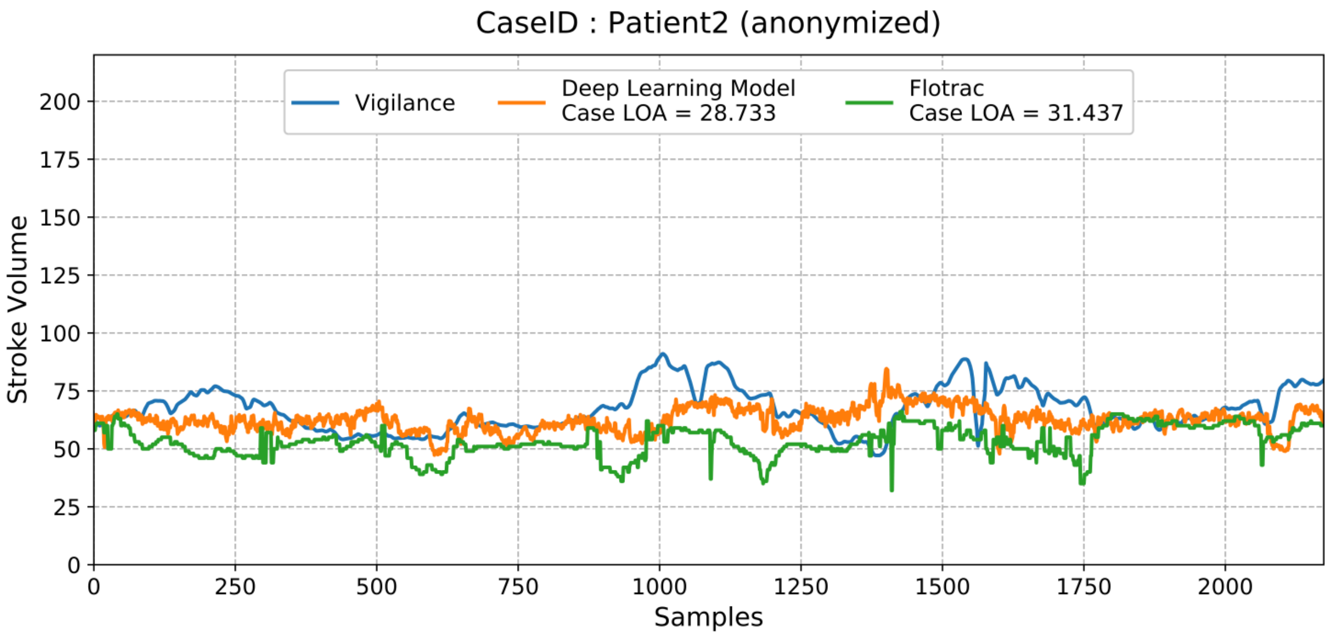

CaseID : Patient2 (anonymized)
Vigilance
Deep Learning Model
Case LOA = 28.733
Flotrac
Case LOA = 31.437
Stroke Volume
Samples
0
25
50
75
100
125
150
175
200
0
250
500
750
1000
1250
1500
1750
2000

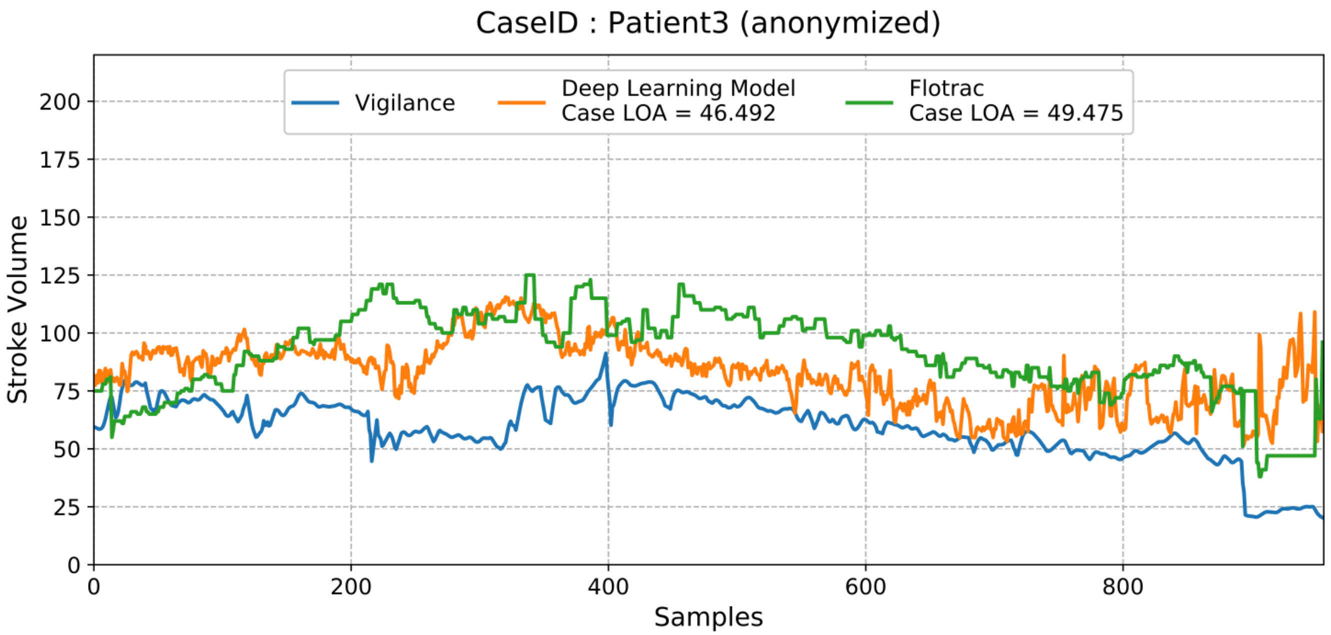

CaseID : Patient3 (anonymized)
Vigilance
Deep Learning Model
Case LOA = 46.492
Flotrac
Case LOA = 49.475
Stroke Volume
Samples
0
25
50
75
100
125
150
175
200
0
200
400
600
800

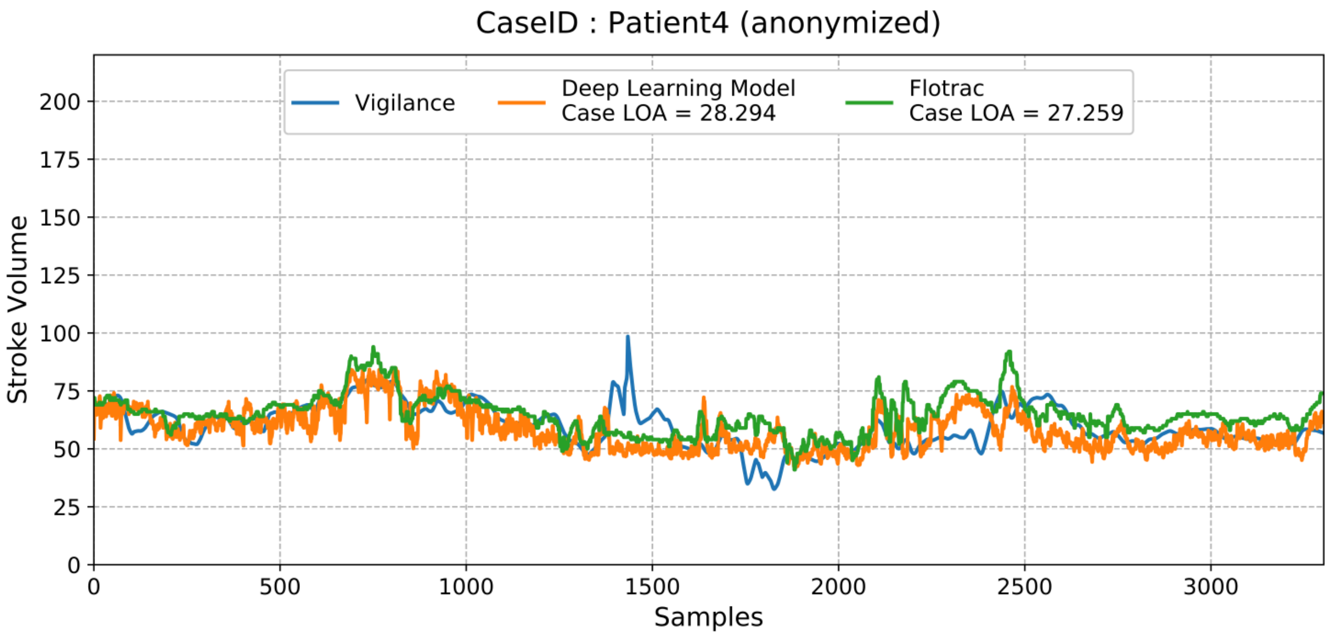
CaseID : Patient4 (anonymized)
Vigilance
Deep Learning Model
Case LOA = 28.294
Flotrac
Case LOA = 27.259
Stroke Volume
Samples
0
25
50
75
100
125
150
175
200
0
500
1000
1500
2000
2500
3000

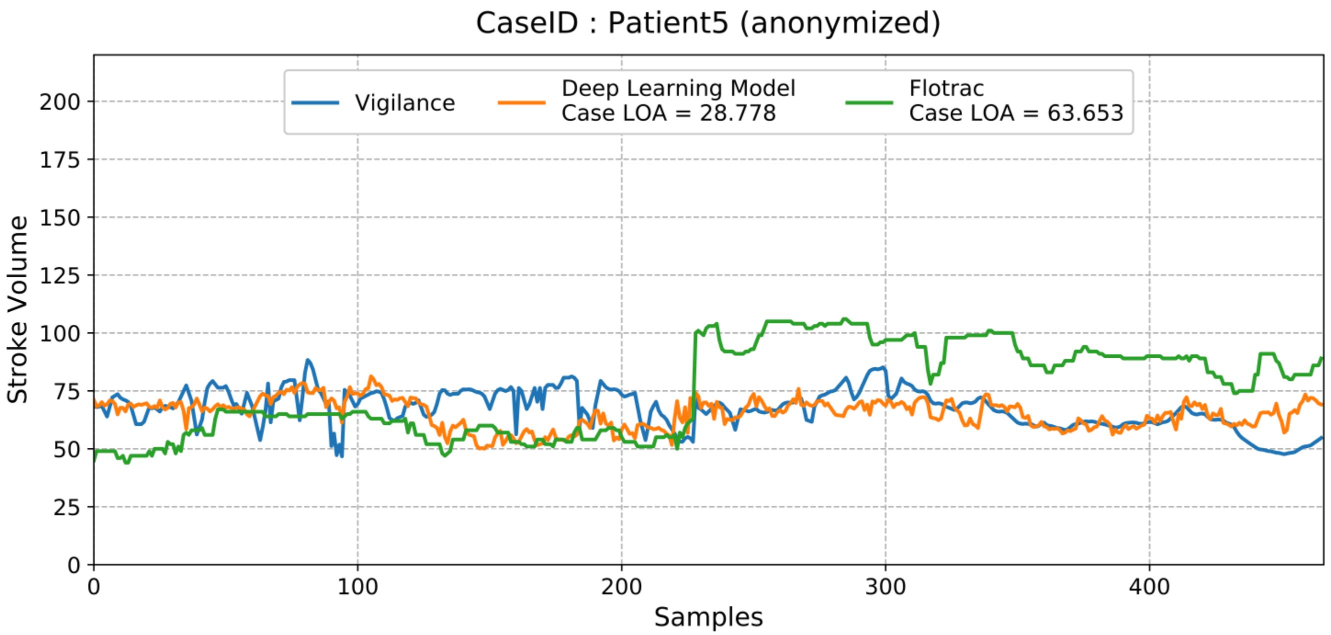

CaseID : Patient5 (anonymized)
Vigilance
Deep Learning Model
Case LOA = 28.778
Flotrac
Case LOA = 63.653
Stroke Volume
200
175
150
125
100
75
50
25
0
0
100
200
300
400
Samples

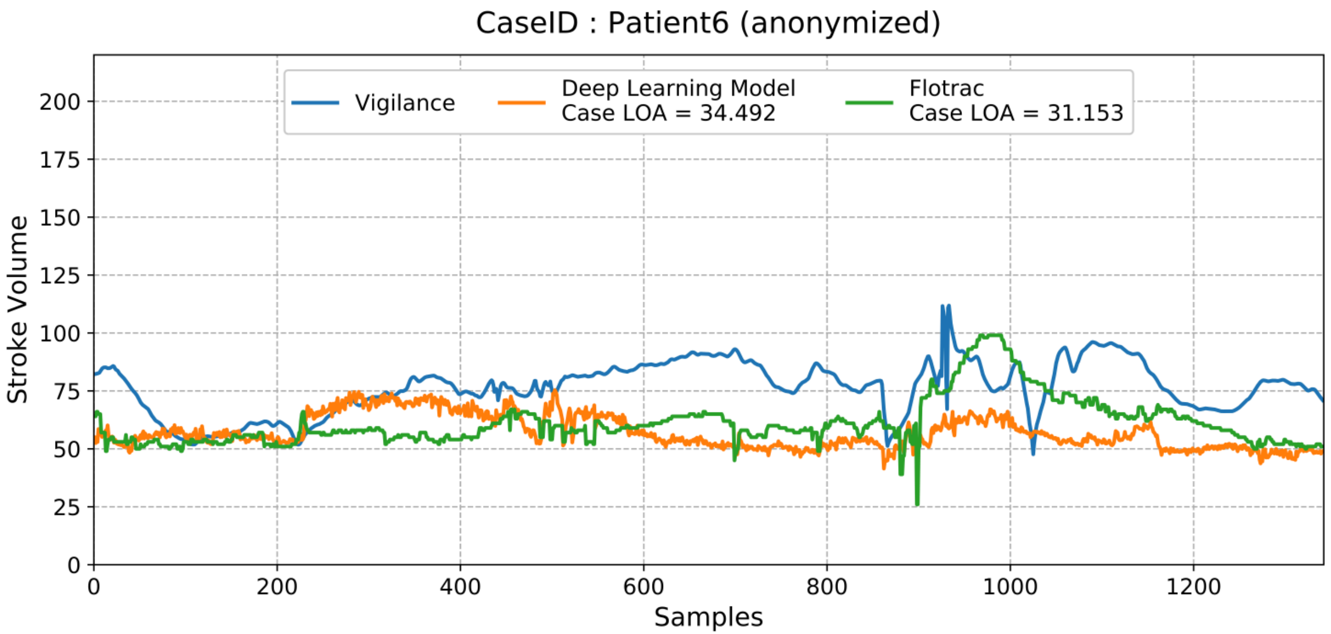
CaseID : Patient6 (anonymized)
Vigilance
Deep Learning Model
Case LOA = 34.492
Flotrac
Case LOA = 31.153
Stroke Volume
Samples
0
25
50
75
100
125
150
175
200
0
200
400
600
800
1000
1200

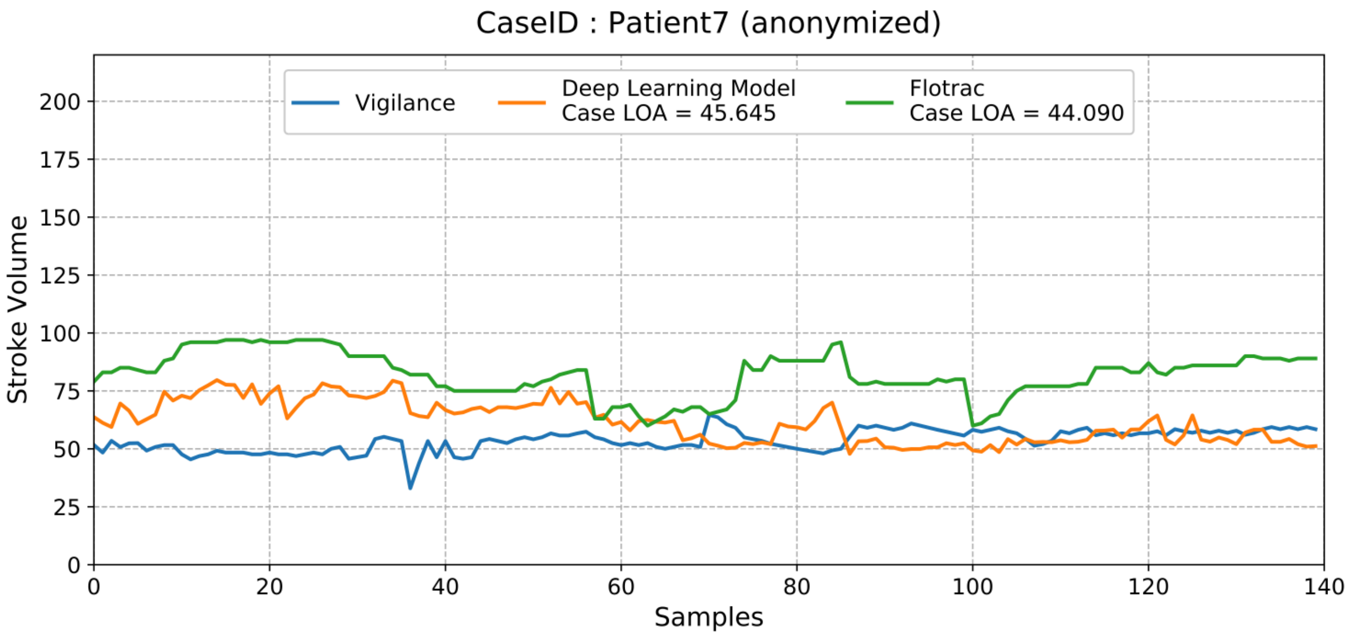

CaseID : Patient7 (anonymized)
Vigilance
Deep Learning Model
Case LOA = 45.645
Flotrac
Case LOA = 44.090
Stroke Volume
Samples
0
25
50
75
100
125
150
175
200
0
20
40
60
80
100
120
140

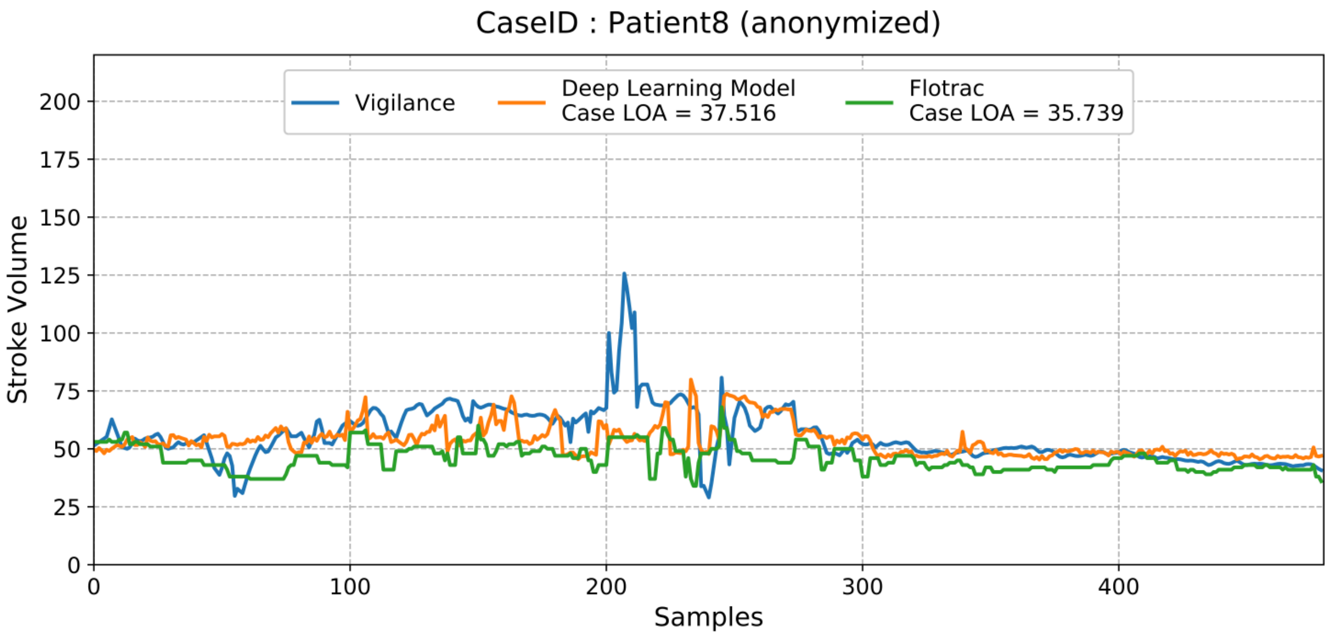

CaseID : Patient8 (anonymized)
Vigilance
Deep Learning Model
Case LOA = 37.516
Flotrac
Case LOA = 35.739
Stroke Volume
Samples
0
25
50
75
100
125
150
175
200
0
100
200
300
400

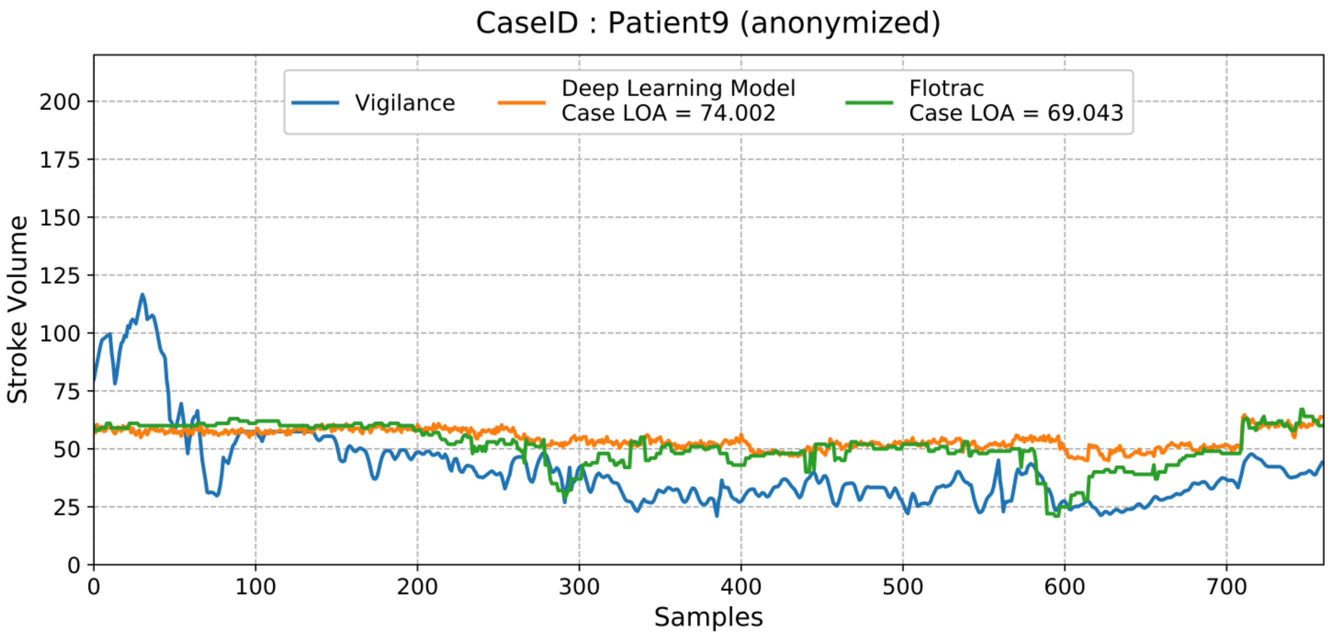

CaseID : Patient9 (anonymized)
Vigilance
Deep Learning Model
Case LOA = 74.002
Flotrac
Case LOA = 69.043
Stroke Volume
Samples
0
25
50
75
100
125
150
175
200
0
100
200
300
400
500
600
700

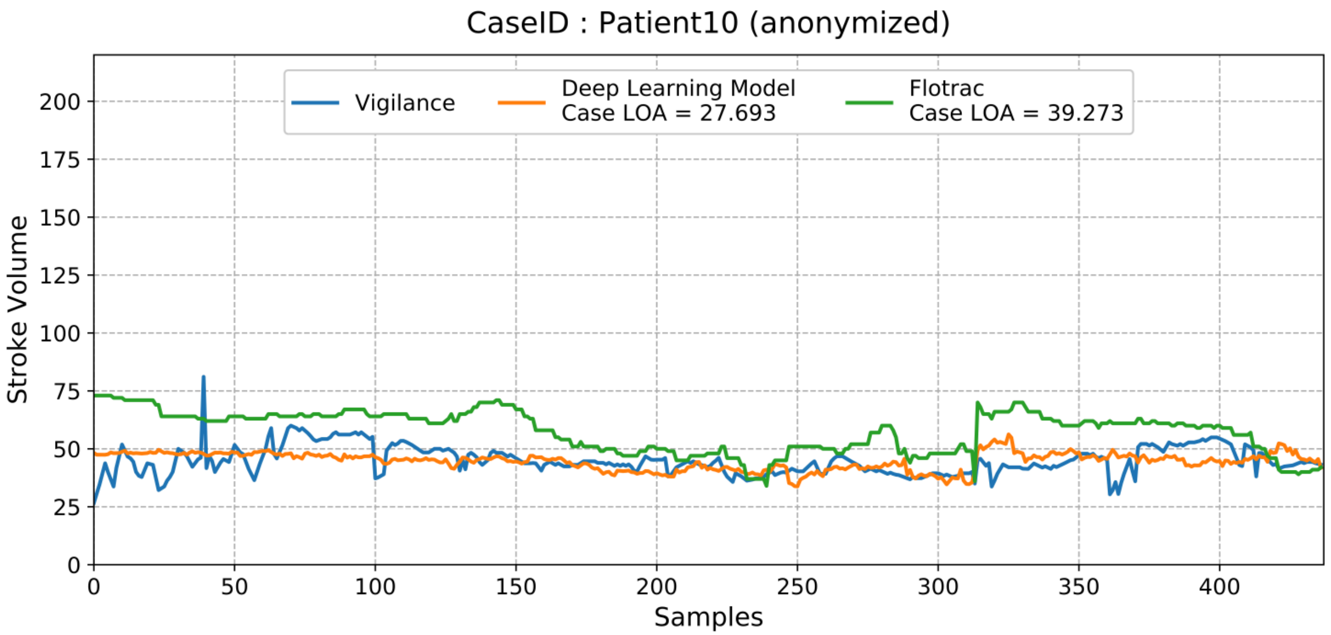

CaseID : Patient10 (anonymized)
Vigilance
Deep Learning Model
Case LOA = 27.693
Flotrac
Case LOA = 39.273
Stroke Volume
Samples
0
25
50
75
100
125
150
175
200
0
50
100
150
200
250
300
350
400

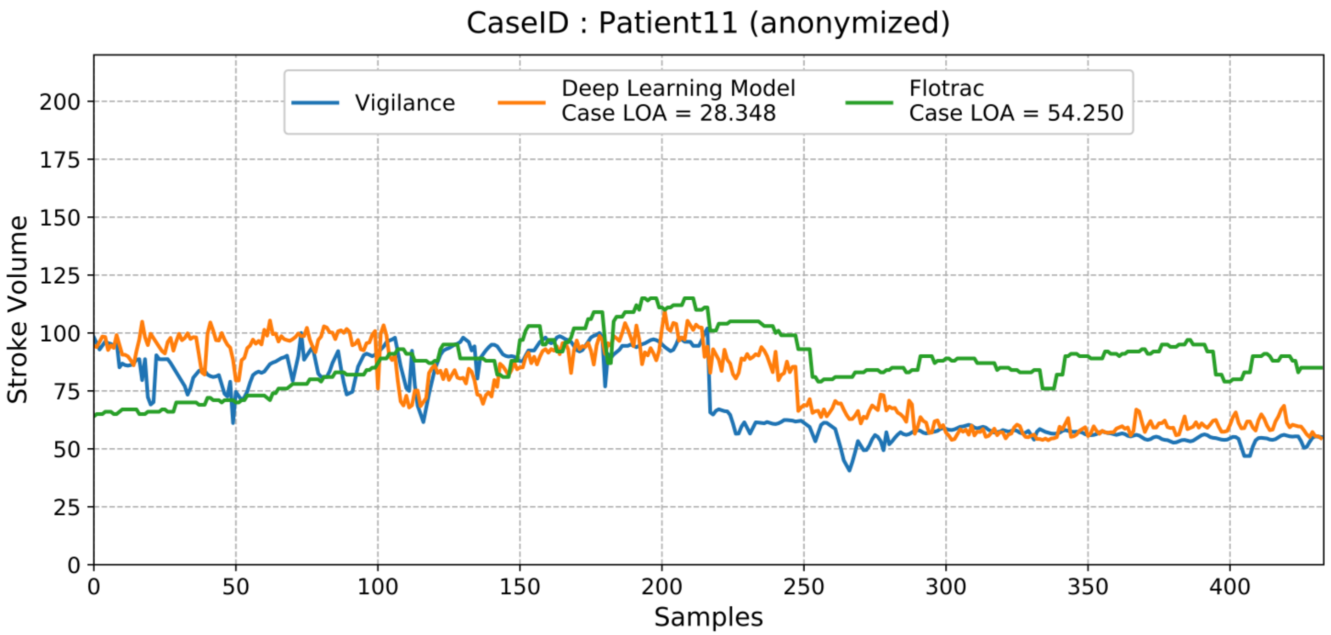
CaseID : Patient11 (anonymized)
Vigilance
Deep Learning Model
Case LOA = 28.348
Flotrac
Case LOA = 54.250
Stroke Volume
Samples
0
25
50
75
100
125
150
175
200
0
50
100
150
200
250
300
350
400

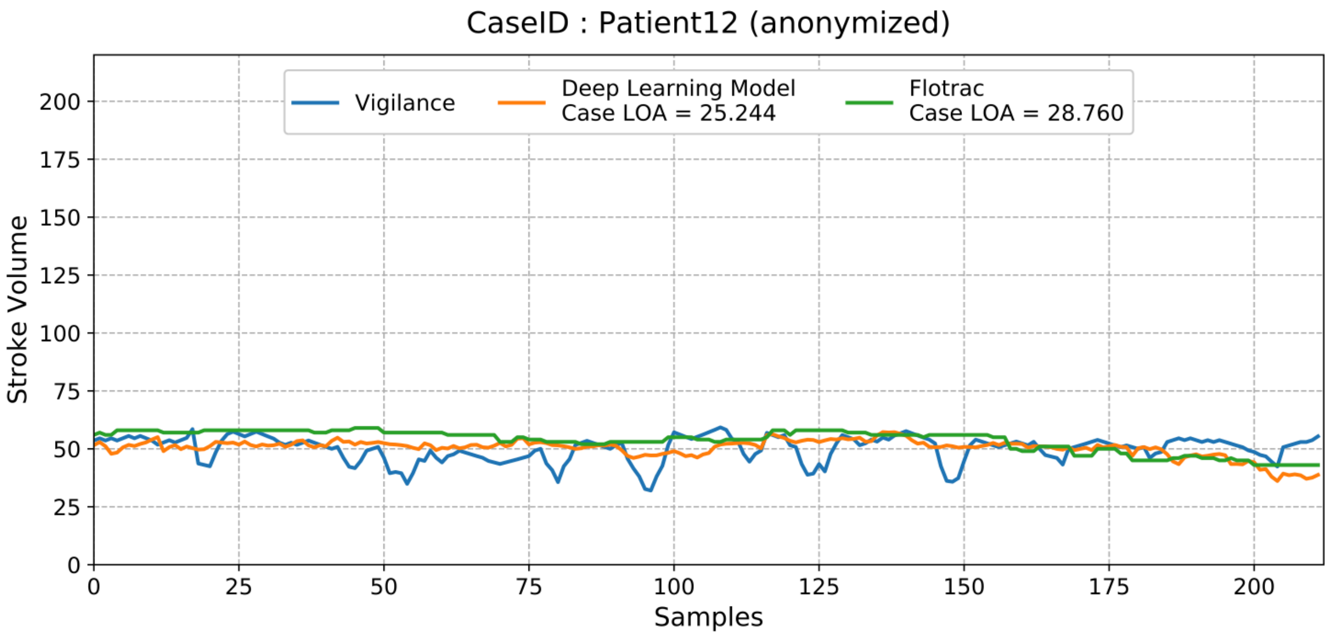

CaseID : Patient12 (anonymized)
Vigilance
Deep Learning Model
Case LOA = 25.244
Flotrac
Case LOA = 28.760
Stroke Volume
Samples
0
25
50
75
100
125
150
175
200

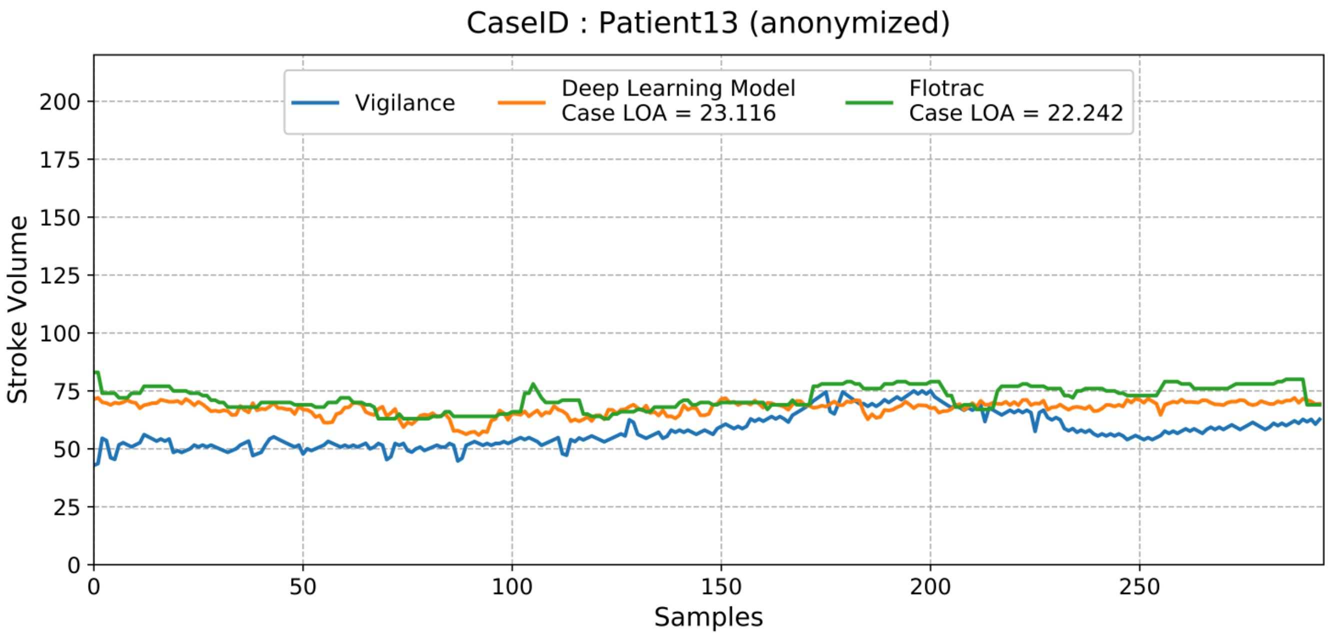

CaseID : Patient13 (anonymized)
Vigilance
Deep Learning Model
Case LOA = 23.116
Flotrac
Case LOA = 22.242
Stroke Volume
Samples
0
25
50
75
100
125
150
175
200
250

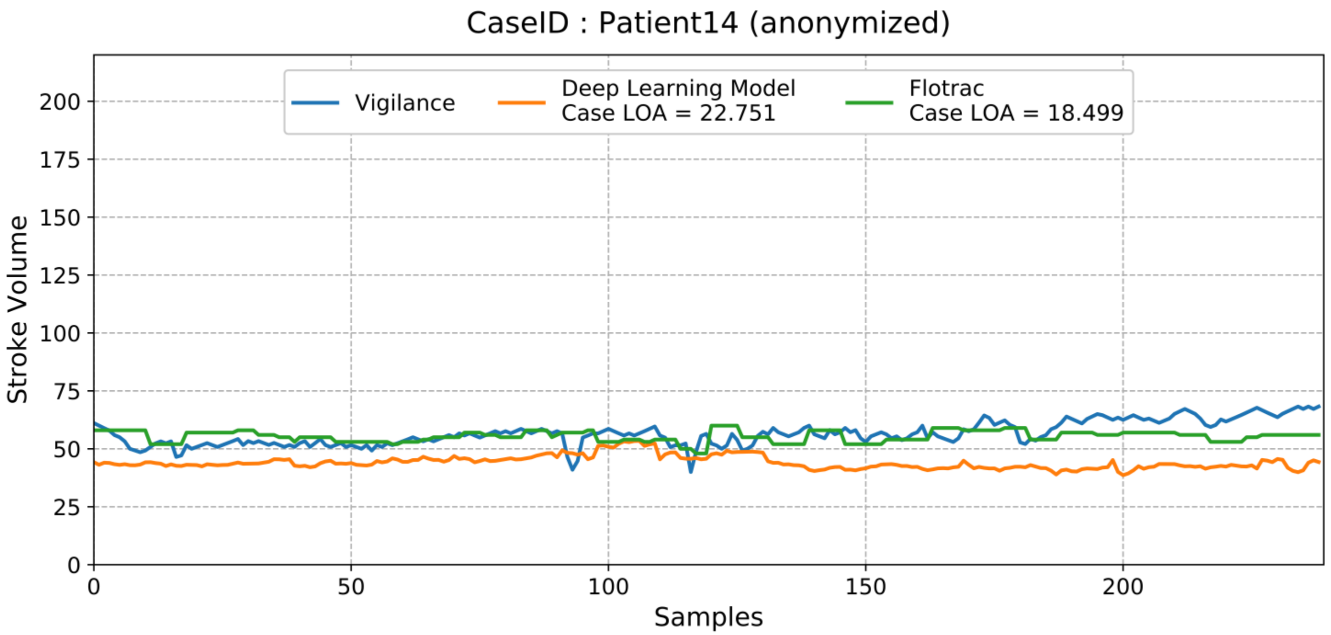

CaseID : Patient14 (anonymized)
Vigilance
Deep Learning Model
Case LOA = 22.751
Flotrac
Case LOA = 18.499
Stroke Volume
0
25
50
75
100
125
150
175
200
0
50
100
150
200
Samples

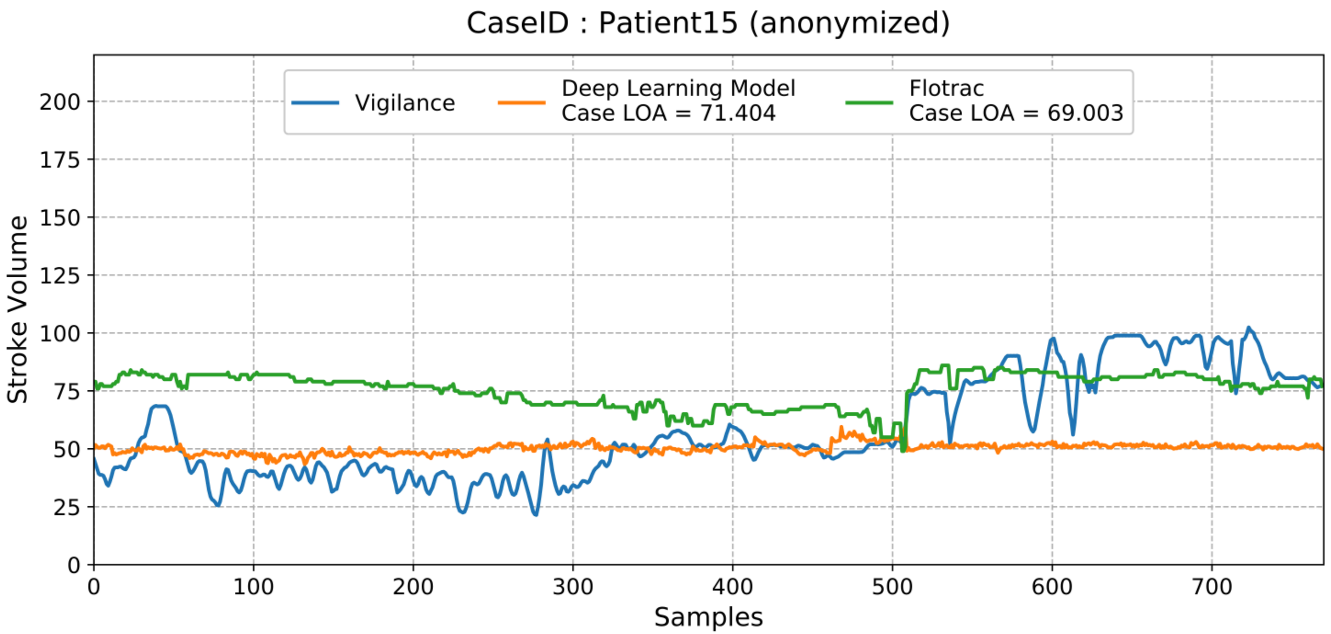

CaseID : Patient15 (anonymized)
Vigilance
Deep Learning Model
Case LOA = 71.404
Flotrac
Case LOA = 69.003
Stroke Volume
Samples
0
25
50
75
100
125
150
175
200
0
100
200
300
400
500
600
700

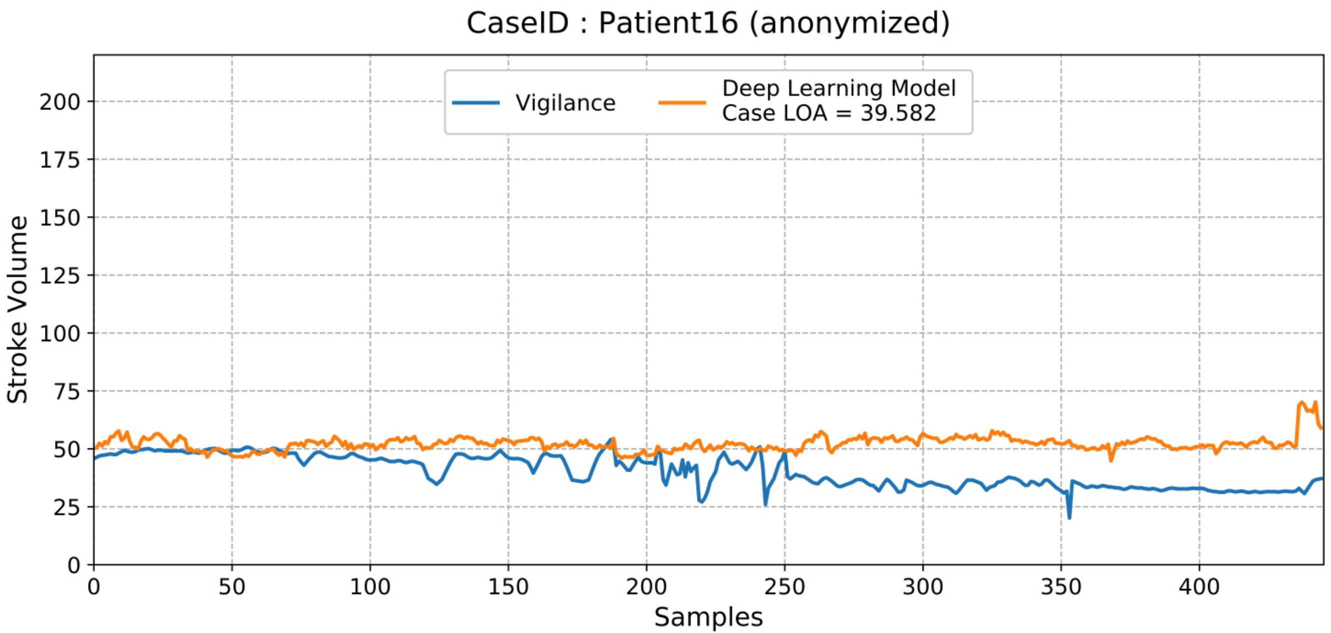

CaseID : Patient16 (anonymized)
Vigilance
Deep Learning Model
Case LOA = 39.582
Stroke Volume
Samples
0
25
50
75
100
125
150
175
200
0
50
100
150
200
250
300
350
400

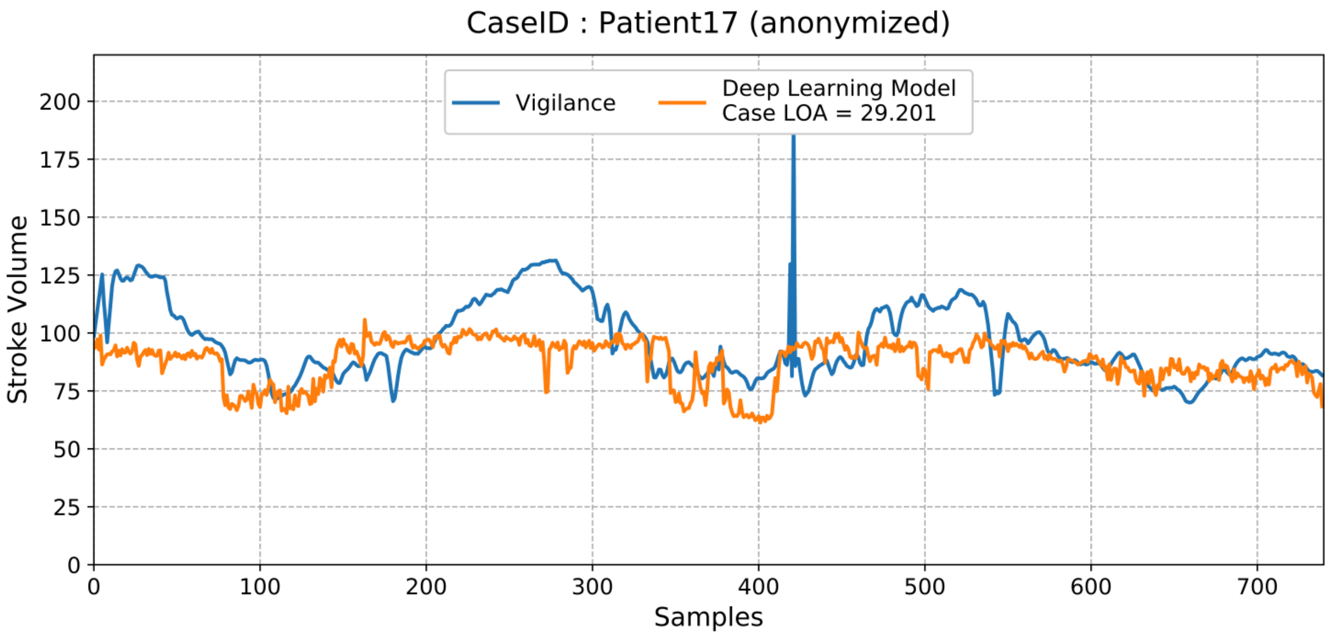
CaseID : Patient17 (anonymized)
Vigilance
Deep Learning Model
Case LOA = 29.201
Stroke Volume
Samples
0
25
50
75
100
125
150
175
200
0
100
200
300
400
500
600
700

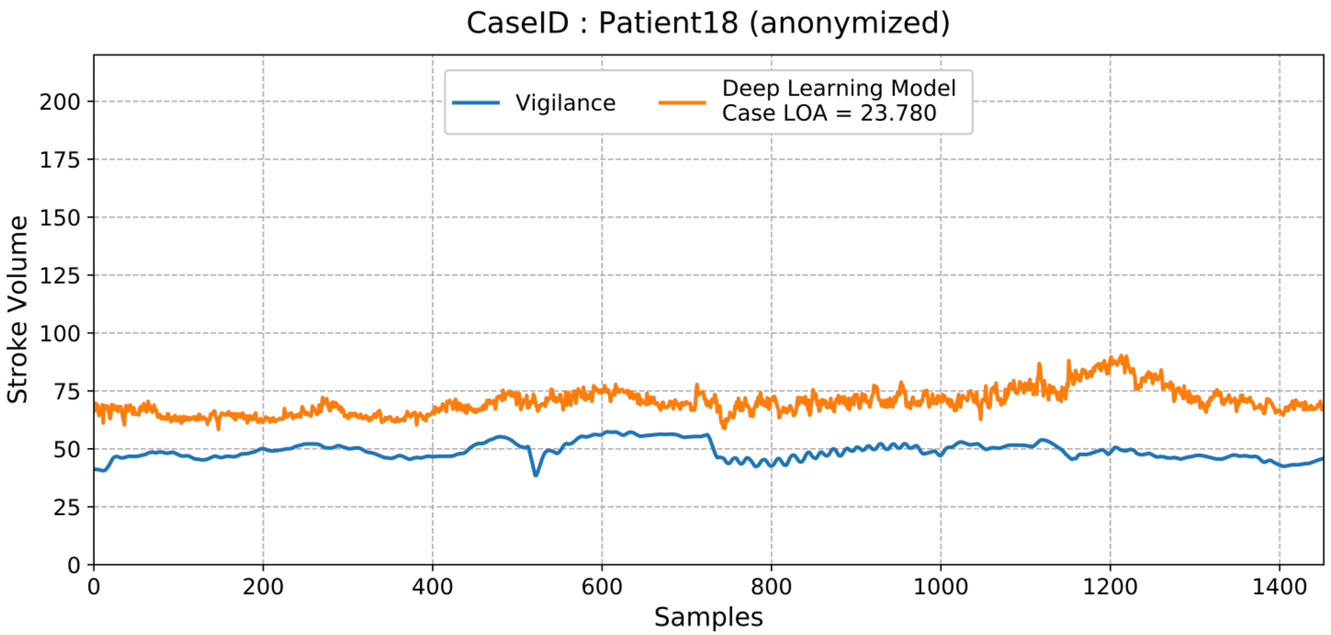
CaseID : Patient18 (anonymized)
Vigilance
Deep Learning Model
Case LOA = 23.780
Stroke Volume
Samples
0
25
50
75
100
125
150
175
200
0
200
400
600
800
1000
1200
1400

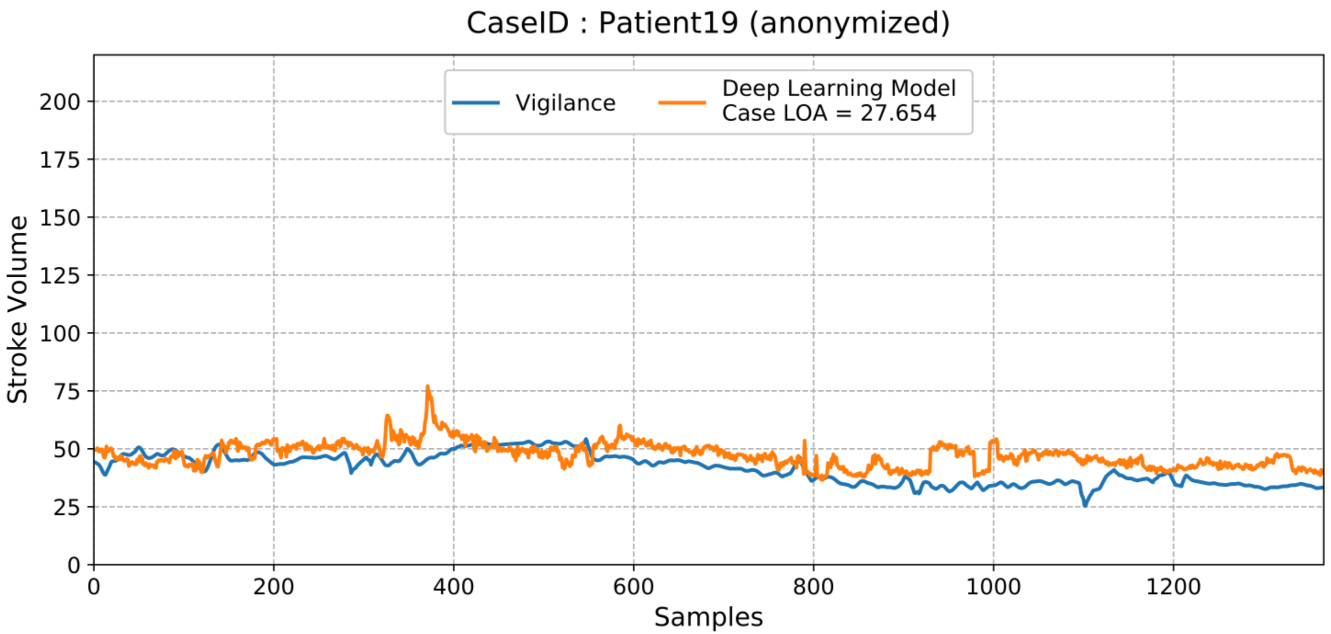
CaseID : Patient19 (anonymized)
Vigilance
Deep Learning Model
Case LOA = 27.654
Stroke Volume
Samples
0
25
50
75
100
125
150
175
200
0
200
400
600
800
1000
1200

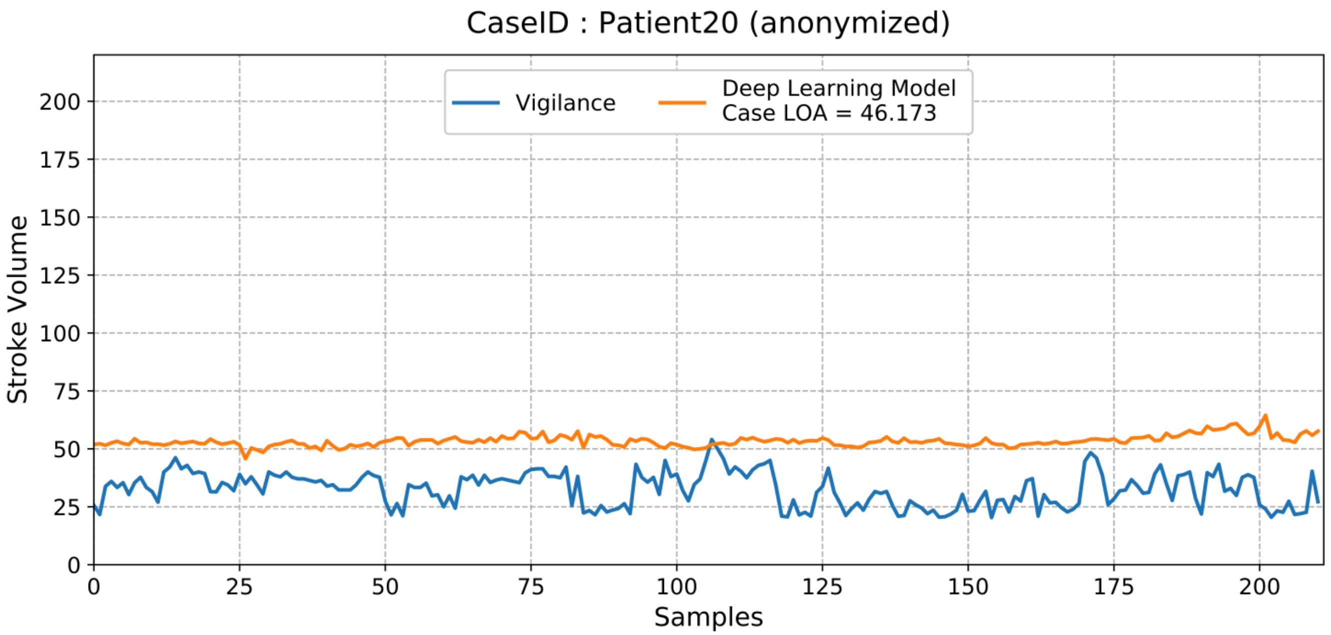

CaseID : Patient20 (anonymized)
Vigilance
Deep Learning Model
Case LOA = 46.173
Stroke Volume
Samples
0
25
50
75
100
125
150
175
200

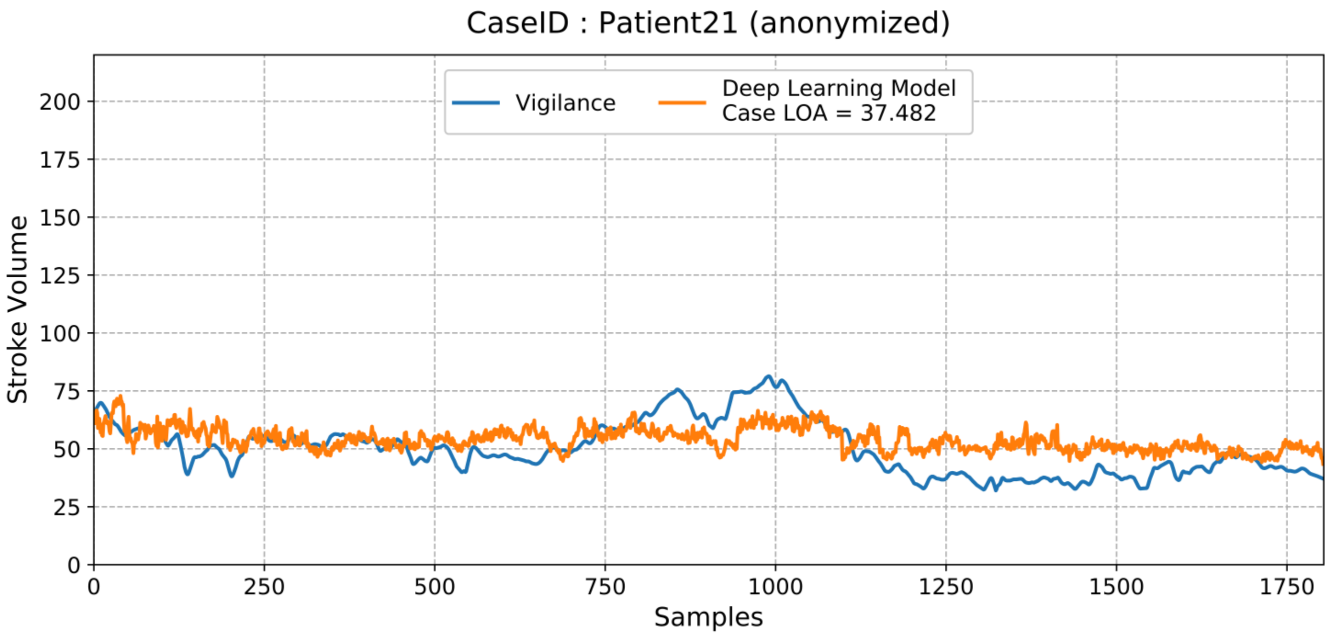

CaseID : Patient21 (anonymized)
Vigilance
Deep Learning Model
Case LOA = 37.482
Stroke Volume
Samples
0
25
50
75
100
125
150
175
200
0
250
500
750
1000
1250
1500
1750

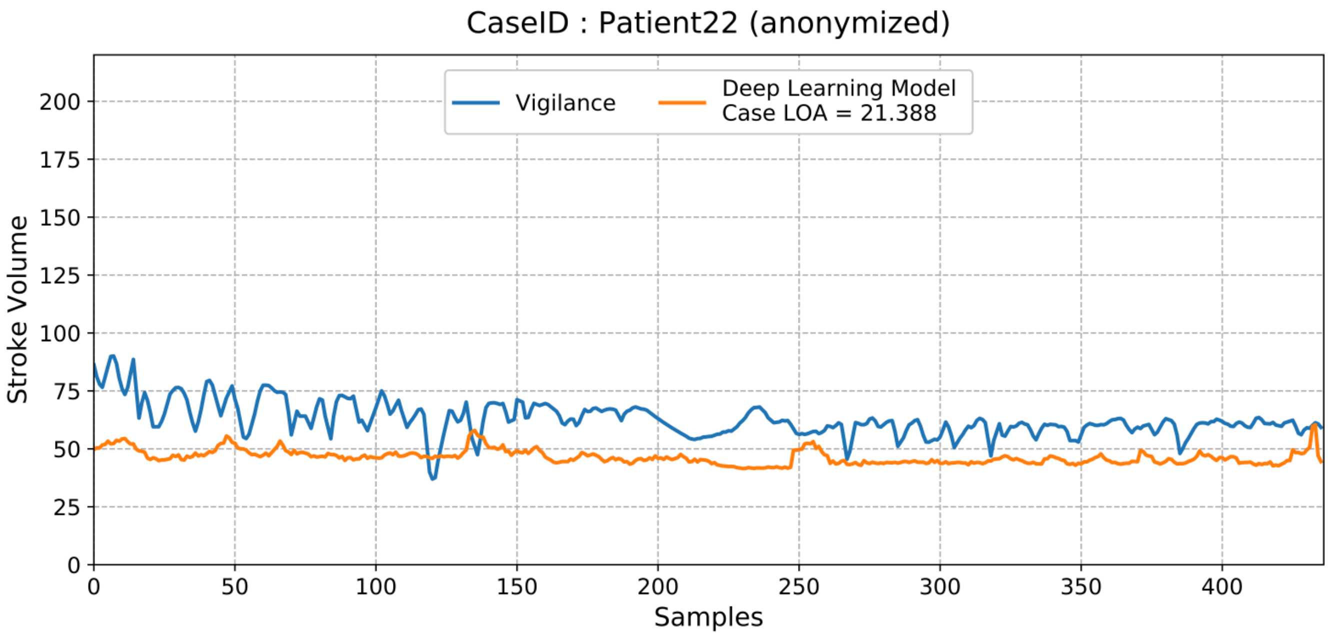

CaseID : Patient22 (anonymized)
Vigilance
Deep Learning Model
Case LOA = 21.388
Stroke Volume
Samples
0
25
50
75
100
125
150
175
200
0
50
100
150
200
250
300
350
400

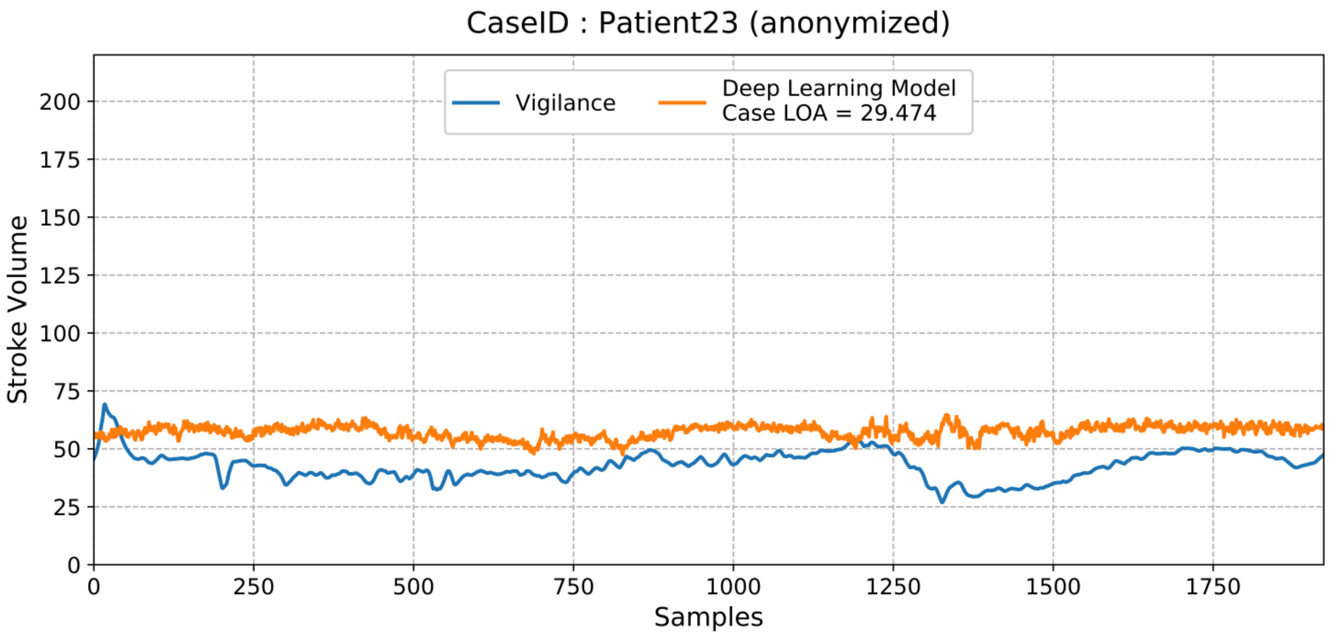

CaseID : Patient23 (anonymized)
Vigilance
Deep Learning Model
Case LOA = 29.474
Stroke Volume
Samples
0
25
50
75
100
125
150
175
200
250
500
750
1000
1250
1500
1750

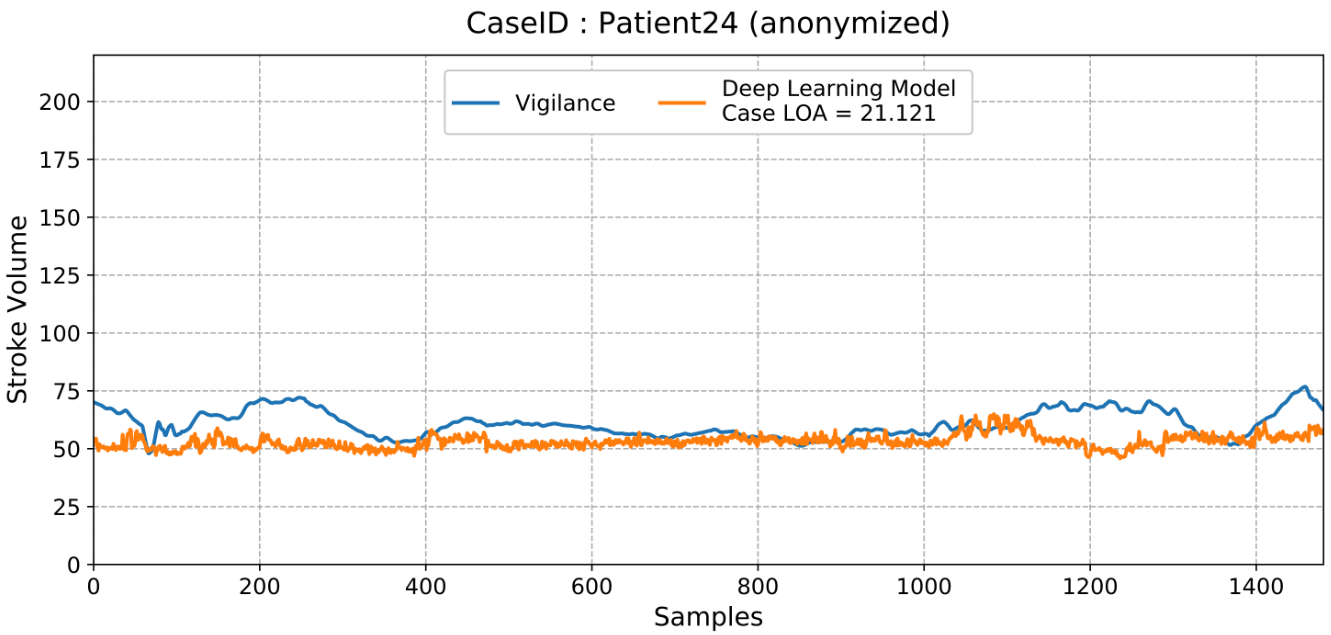
CaseID : Patient24 (anonymized)
Vigilance
Deep Learning Model
Case LOA = 21.121
Stroke Volume
200
175
150
125
100
75
50
25
0
0
200
400
600
800
1000
1200
1400
Samples

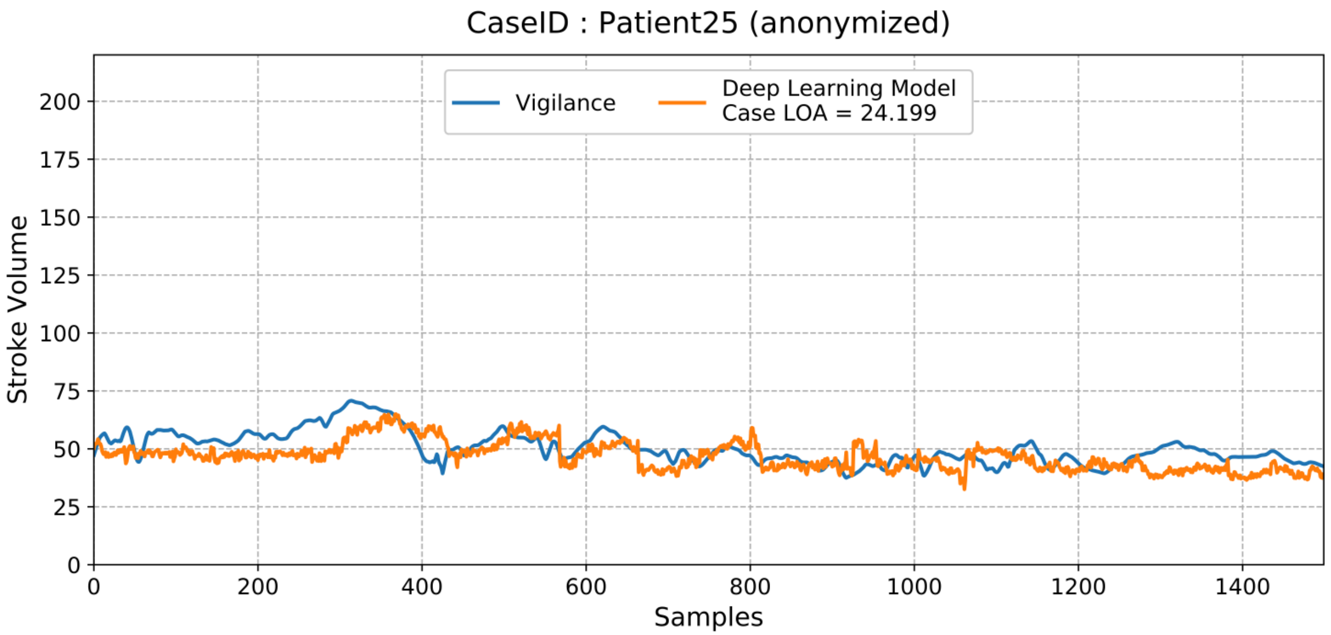

CaseID : Patient25 (anonymized)
Vigilance
Deep Learning Model
Case LOA = 24.199
Stroke Volume
0
25
50
75
100
125
150
175
200
0
200
400
600
800
1000
1200
1400
Samples

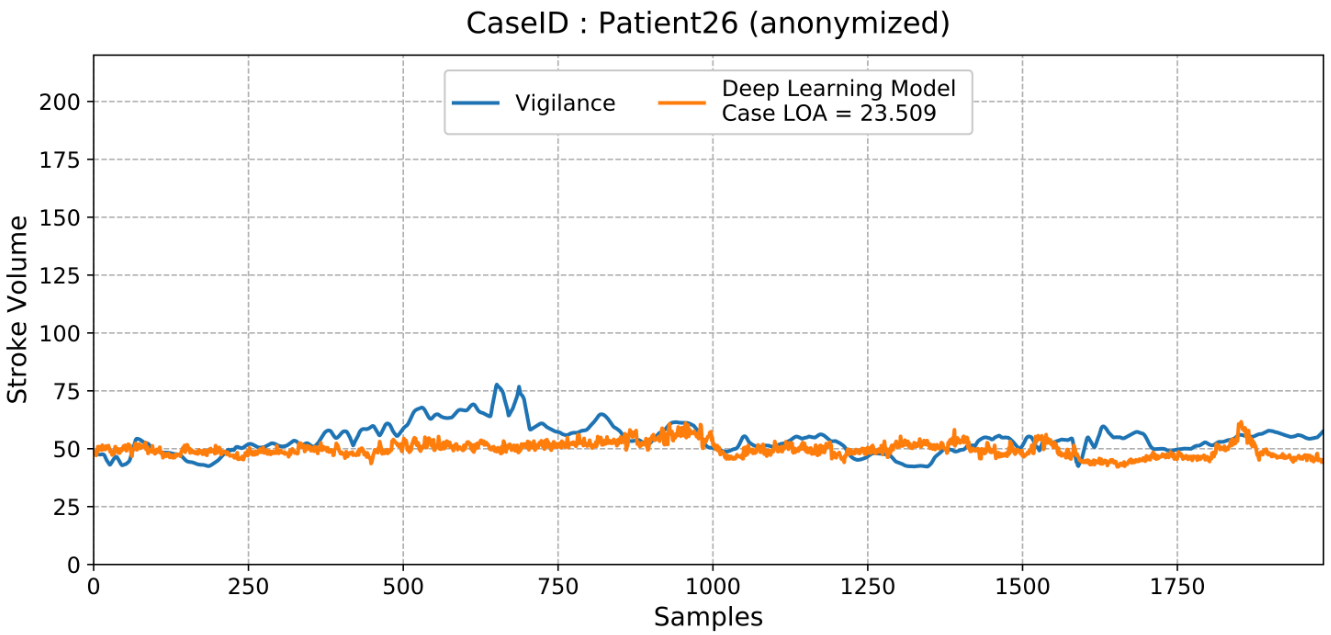

CaseID : Patient26 (anonymized)
Vigilance
Deep Learning Model
Case LOA = 23.509
Stroke Volume
Samples
0
25
50
75
100
125
150
175
200
250
500
750
1000
1250
1500
1750

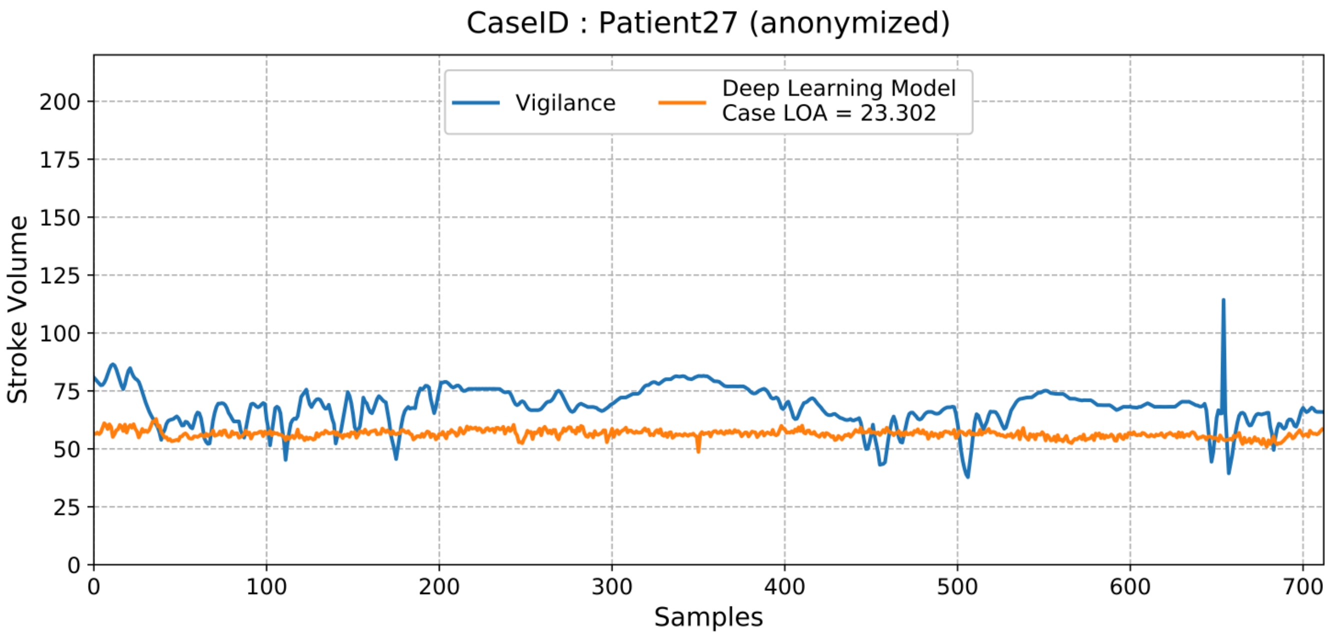

CaseID : Patient27 (anonymized)
Vigilance
Deep Learning Model
Case LOA = 23.302
Stroke Volume
Samples
0
25
50
75
100
125
150
175
200
0
100
200
300
400
500
600
700

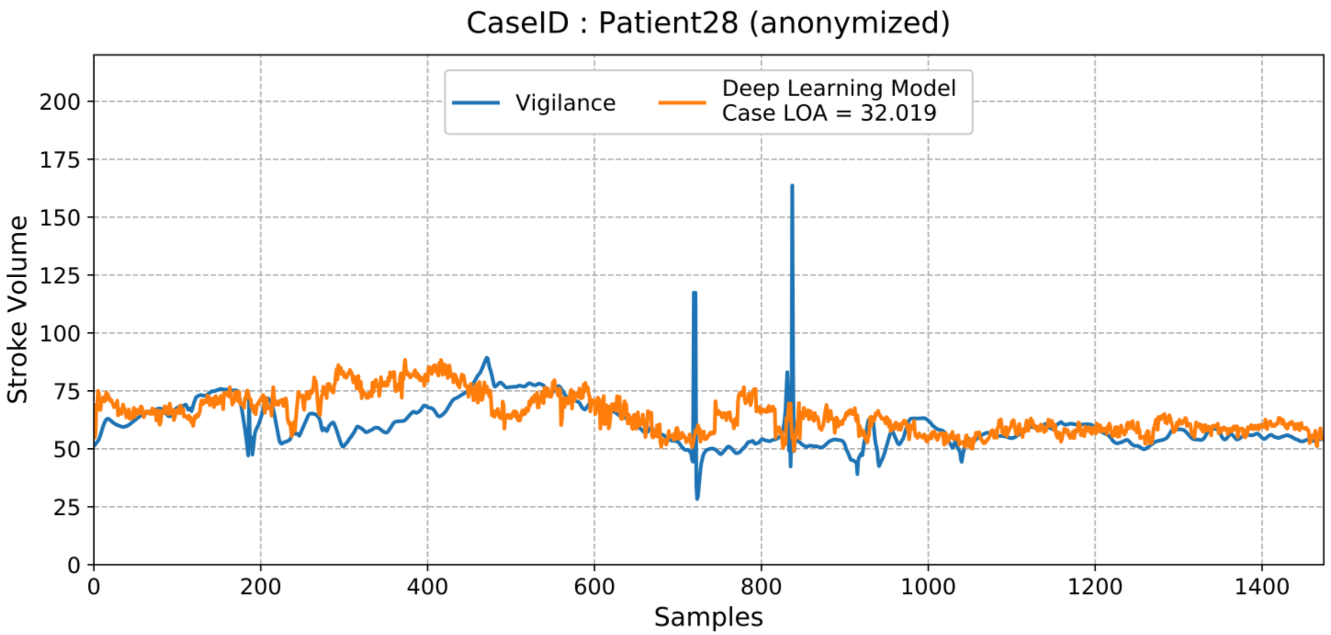
CaseID : Patient28 (anonymized)
Vigilance
Deep Learning Model
Case LOA = 32.019
Stroke Volume
Samples
0
25
50
75
100
125
150
175
200
0
200
400
600
800
1000
1200
1400

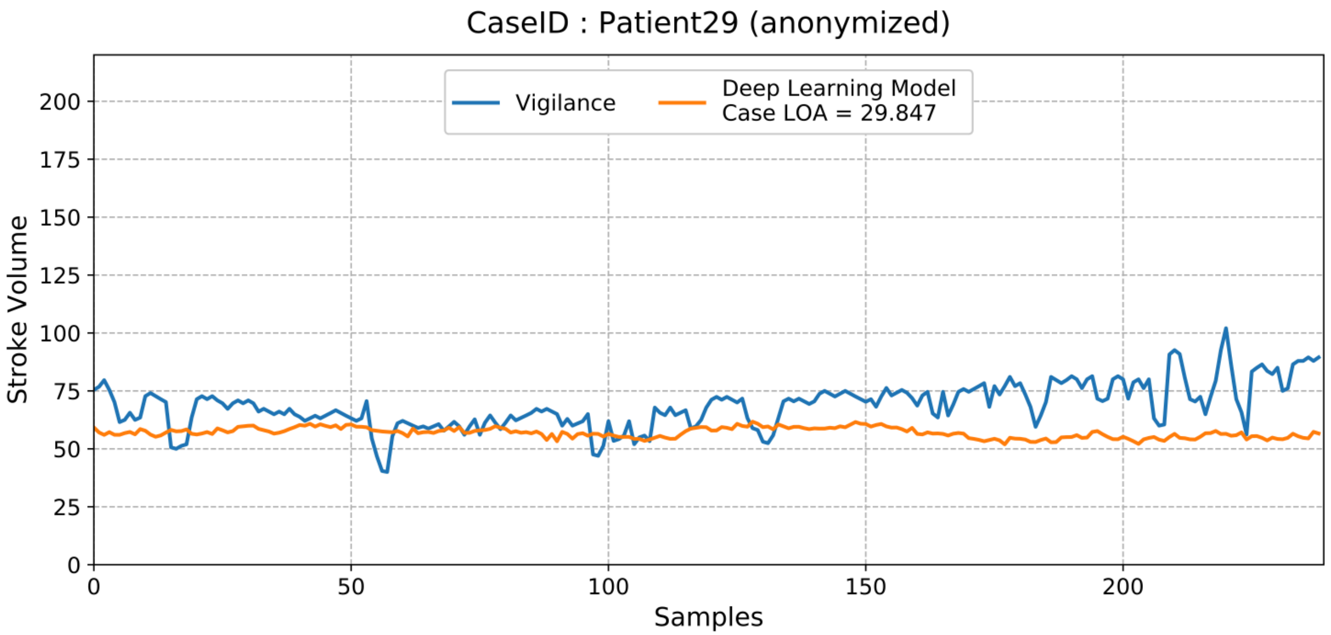
CaseID : Patient29 (anonymized)
Vigilance
Deep Learning Model
Case LOA = 29.847
Stroke Volume
Samples
0
25
50
75
100
125
150
175
200
0
50
100
150
200

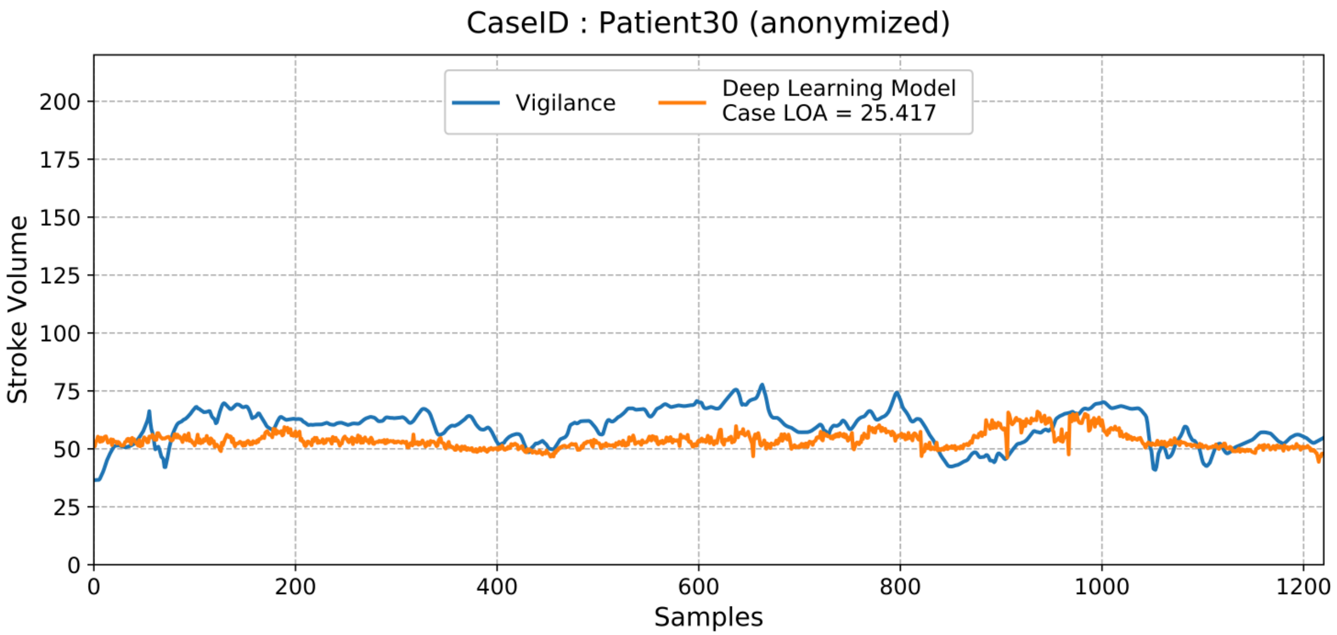

CaseID : Patient30 (anonymized)
Vigilance
Deep Learning Model
Case LOA = 25.417
Stroke Volume
Samples
0
25
50
75
100
125
150
175
200
0
200
400
600
800
1000
1200

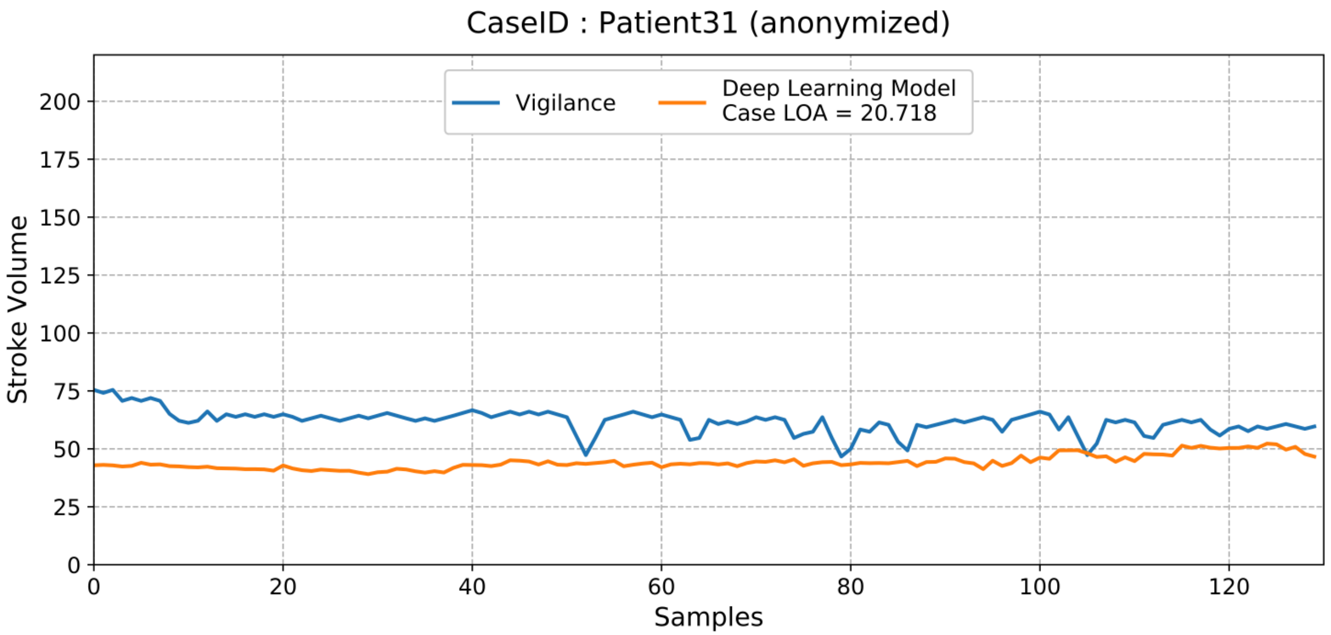

CaseID : Patient31 (anonymized)
Vigilance
Deep Learning Model
Case LOA = 20.718
Stroke Volume
200
175
150
125
100
75
50
25
0
20
40
60
80
100
120
Samples

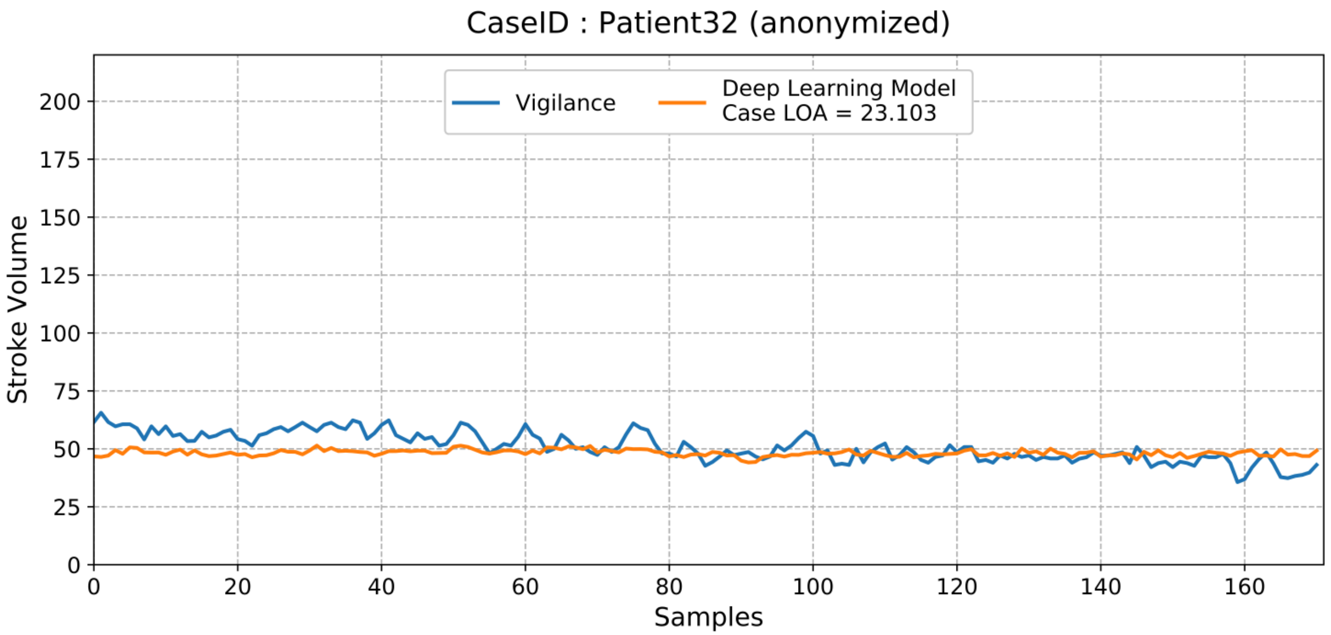
CaseID : Patient32 (anonymized)
Vigilance
Deep Learning Model
Case LOA = 23.103
Stroke Volume
Samples
0
25
50
75
100
125
150
175
200
0
20
40
60
80
100
120
140
160

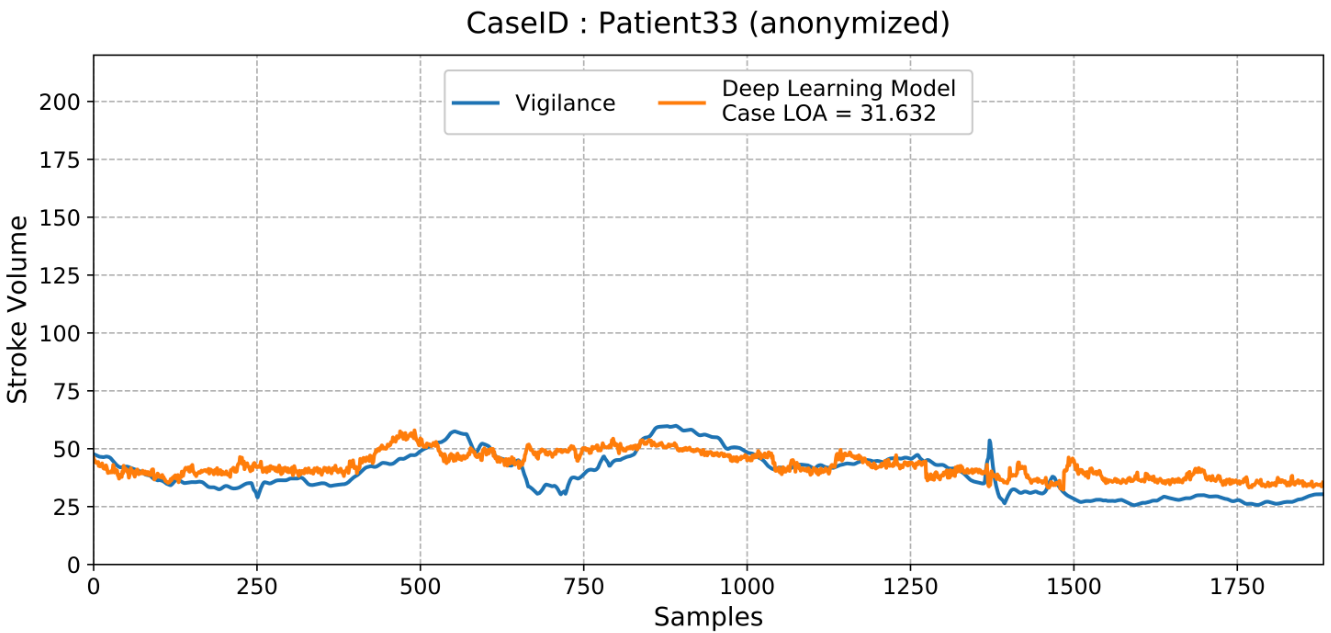

CaseID : Patient33 (anonymized)
Vigilance
Deep Learning Model
Case LOA = 31.632
Stroke Volume
Samples
0
25
50
75
100
125
150
175
200
0
250
500
750
1000
1250
1500
1750

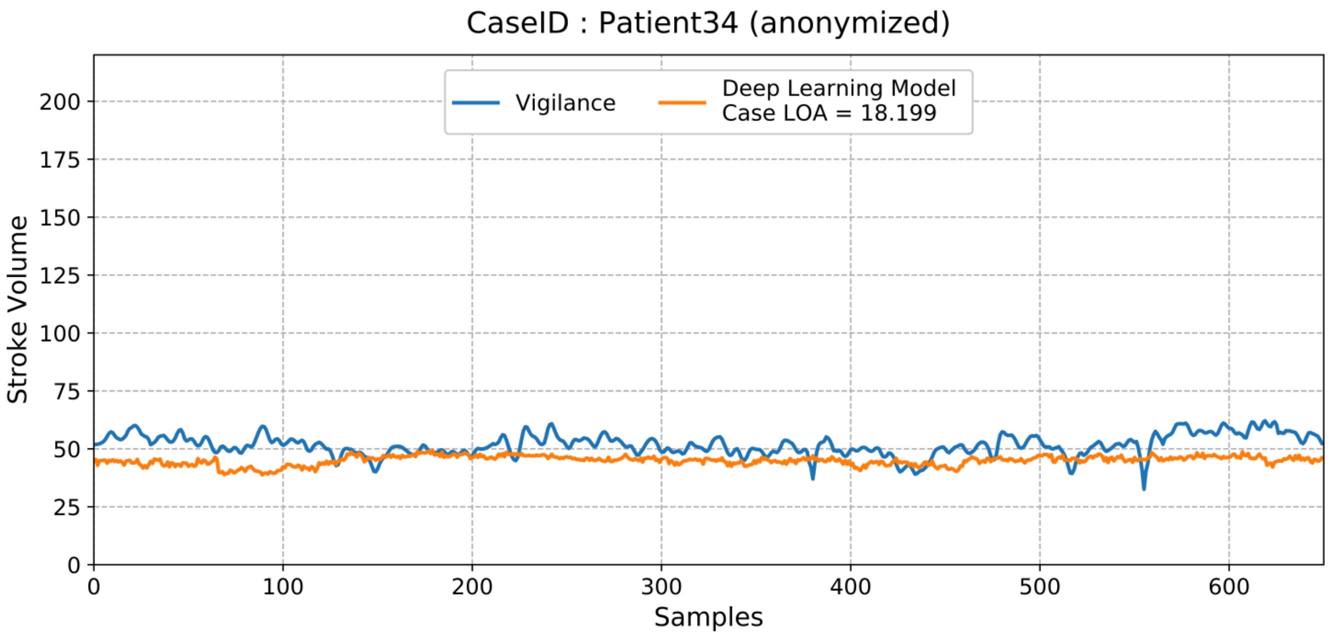

CaseID : Patient34 (anonymized)
Vigilance
Deep Learning Model
Case LOA = 18.199
Stroke Volume
Samples
0
25
50
75
100
125
150
175
200
0
100
200
300
400
500
600

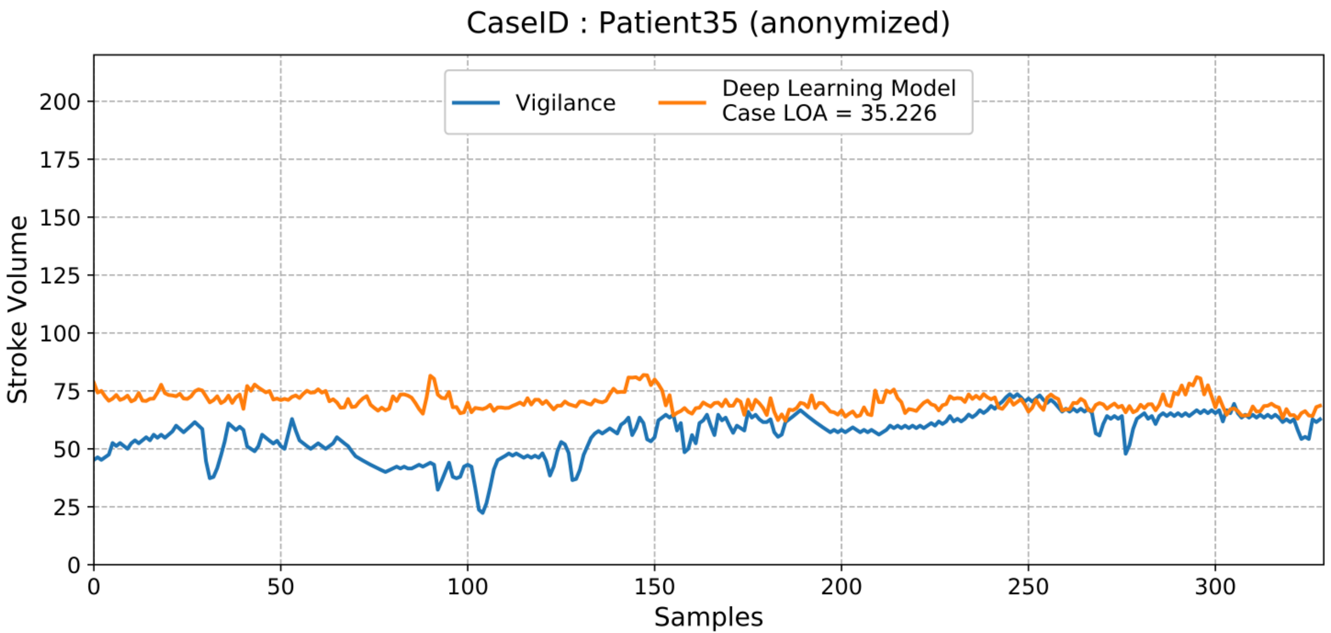

CaseID : Patient35 (anonymized)
Vigilance
Deep Learning Model
Case LOA = 35.226
Stroke Volume
Samples
0
25
50
75
100
125
150
175
200
0
50
100
150
200
250
300

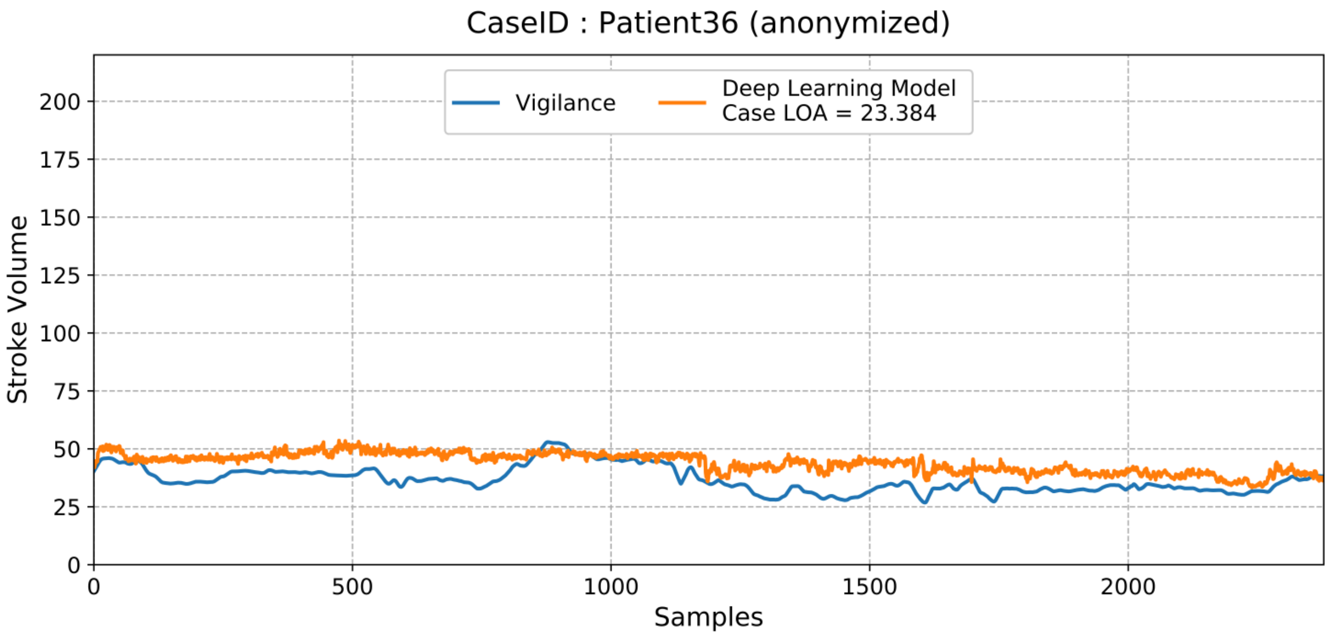
CaseID : Patient36 (anonymized)
Vigilance
Deep Learning Model
Case LOA = 23.384
Stroke Volume
Samples
0
25
50
75
100
125
150
175
200
500
1000
1500
2000

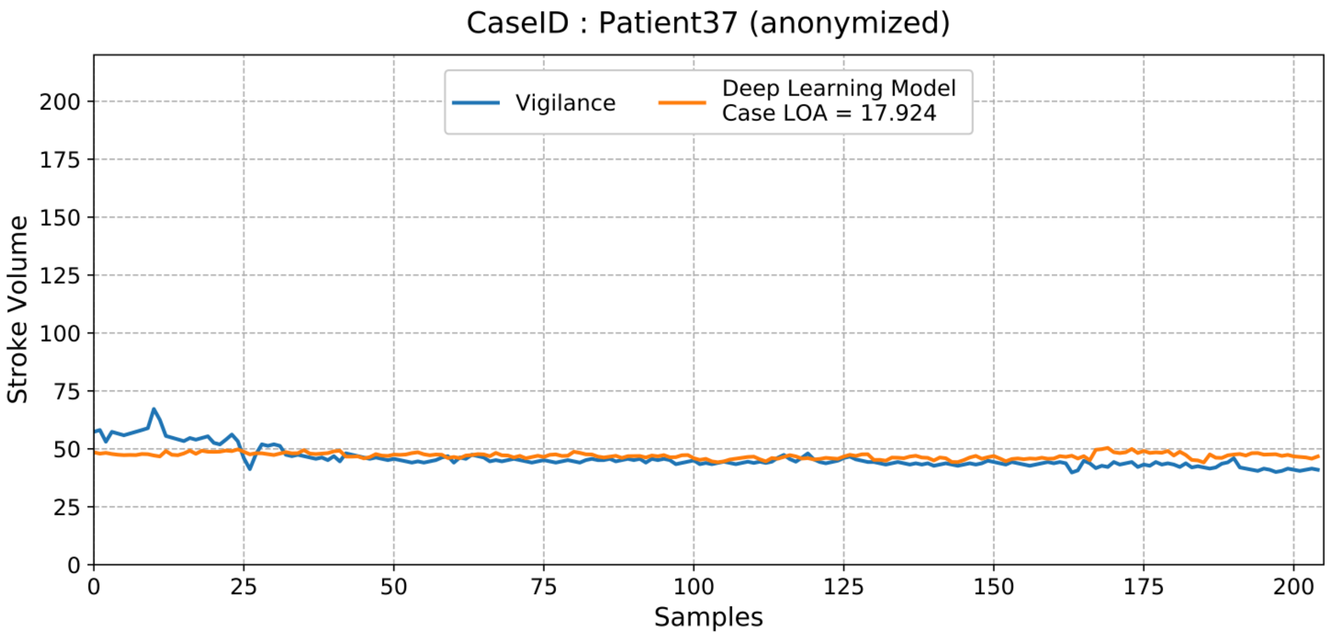

CaseID : Patient37 (anonymized)
Vigilance
Deep Learning Model
Case LOA = 17.924
Stroke Volume
Samples
0
25
50
75
100
125
150
175
200

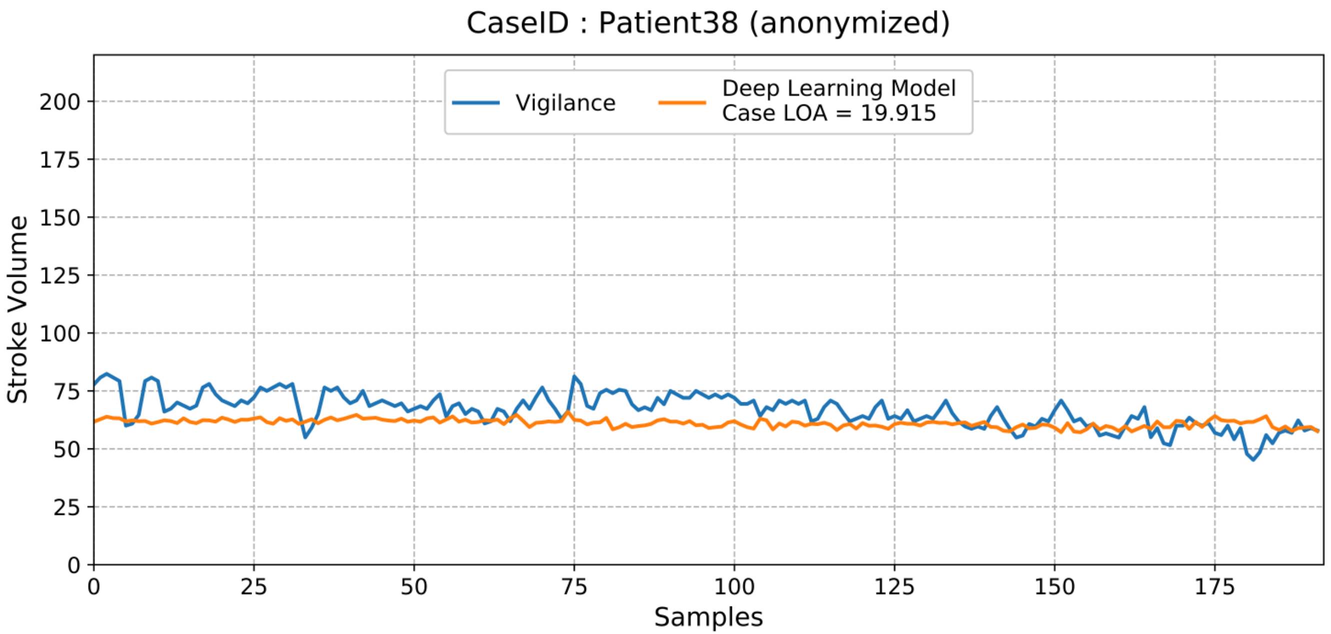

CaseID : Patient38 (anonymized)
Vigilance
Deep Learning Model
Case LOA = 19.915
Stroke Volume
Samples
0
25
50
75
100
125
150
175
200

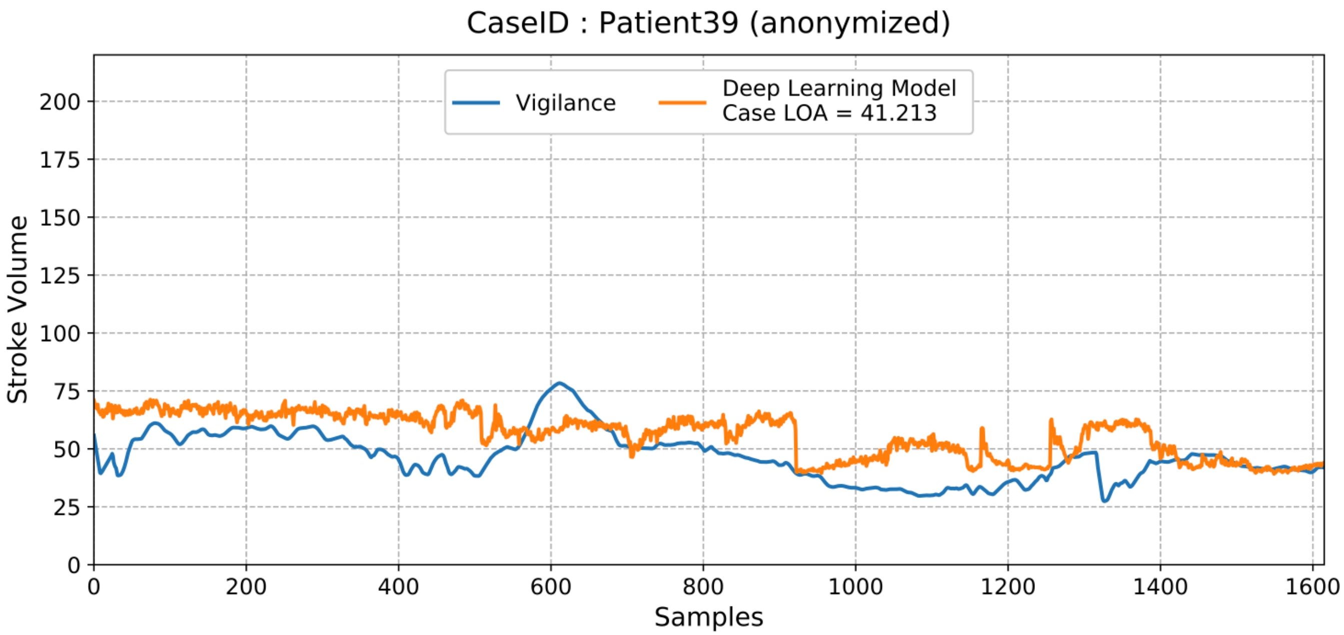

CaseID : Patient39 (anonymized)
Vigilance
Deep Learning Model
Case LOA = 41.213
Stroke Volume
Samples
0
25
50
75
100
125
150
175
200
0
200
400
600
800
1000
1200
1400
1600

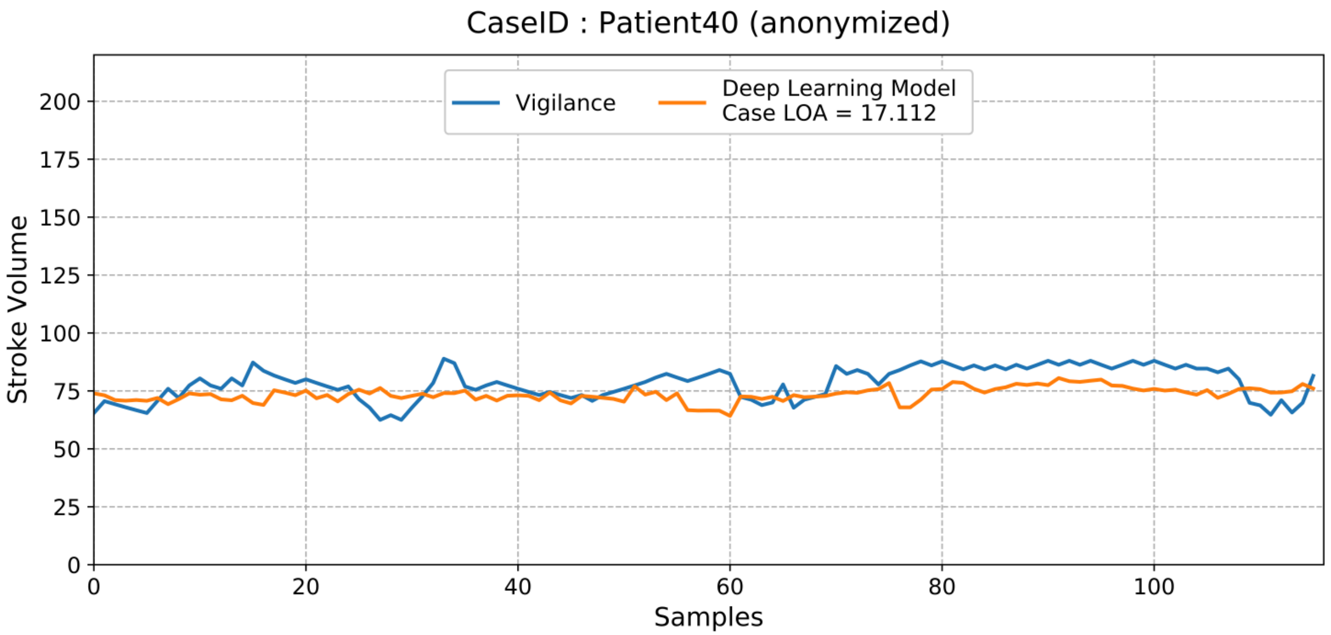
CaseID : Patient40 (anonymized)
Vigilance
Deep Learning Model
Case LOA = 17.112
Stroke Volume
Samples
0
25
50
75
100
125
150
175
200
0
20
40
60
80
100

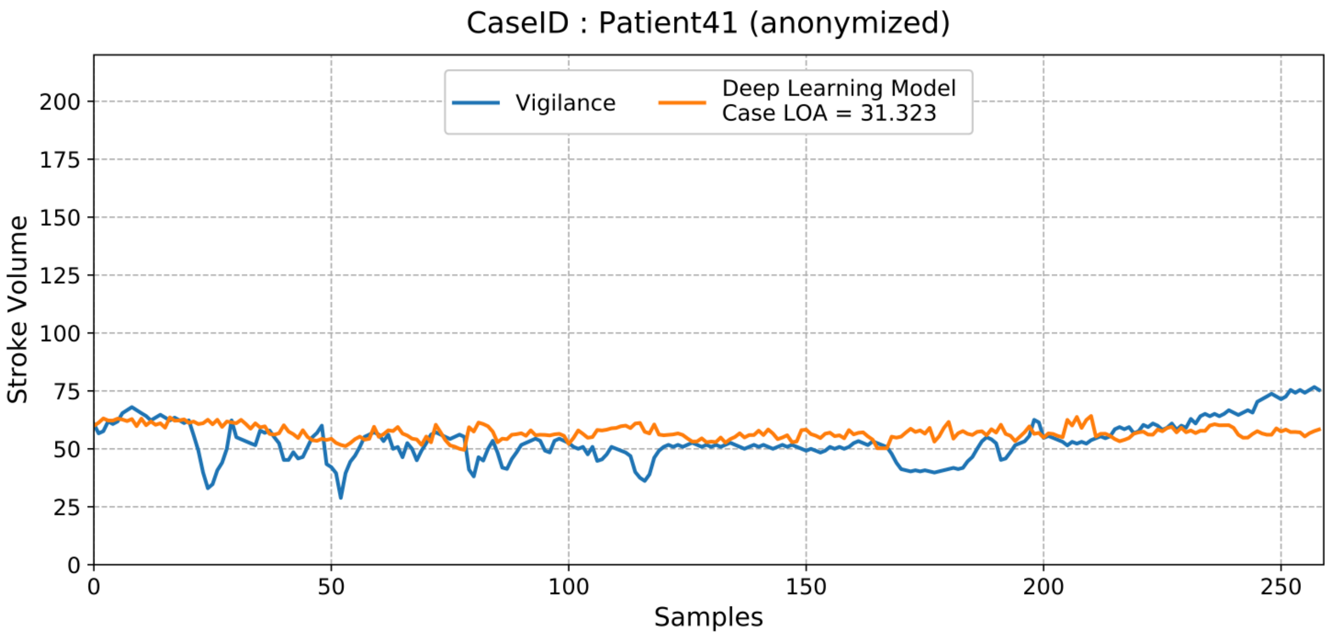

CaseID : Patient41 (anonymized)
Vigilance
Deep Learning Model
Case LOA = 31.323
Stroke Volume
Samples
0
25
50
75
100
125
150
175
200
0
50
100
150
200
250

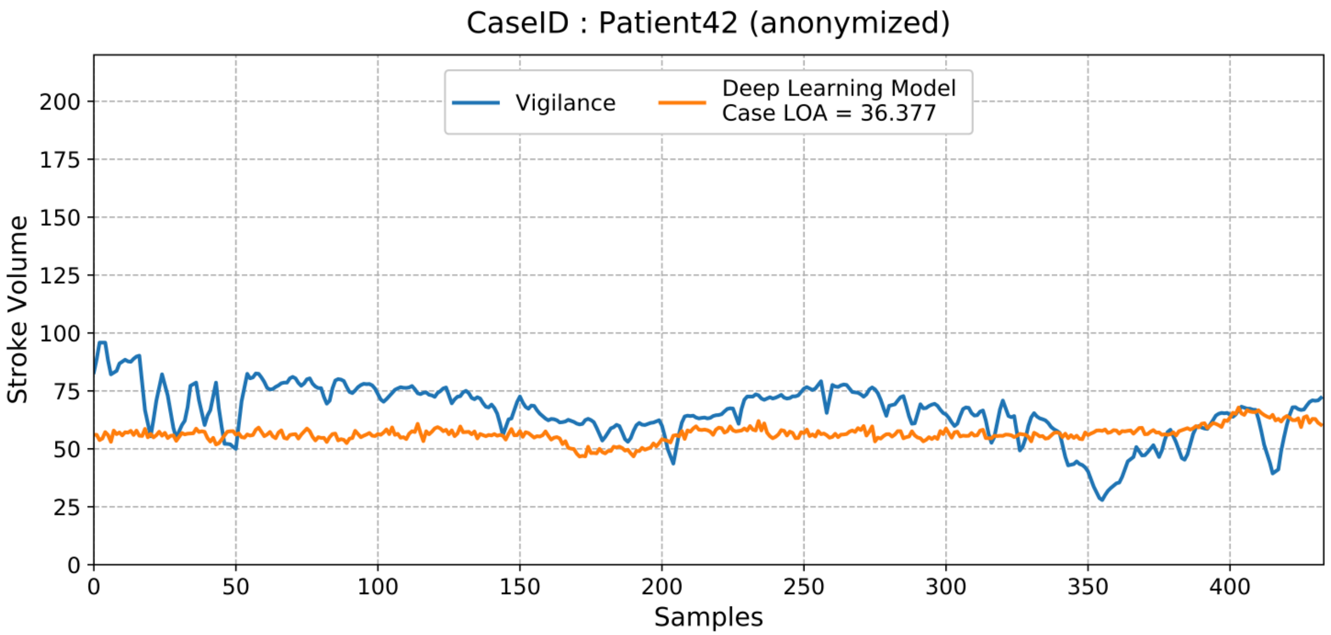

CaseID : Patient42 (anonymized)
Vigilance
Deep Learning Model
Case LOA = 36.377
Stroke Volume
Samples
0
25
50
75
100
125
150
175
200
0
50
100
150
200
250
300
350
400

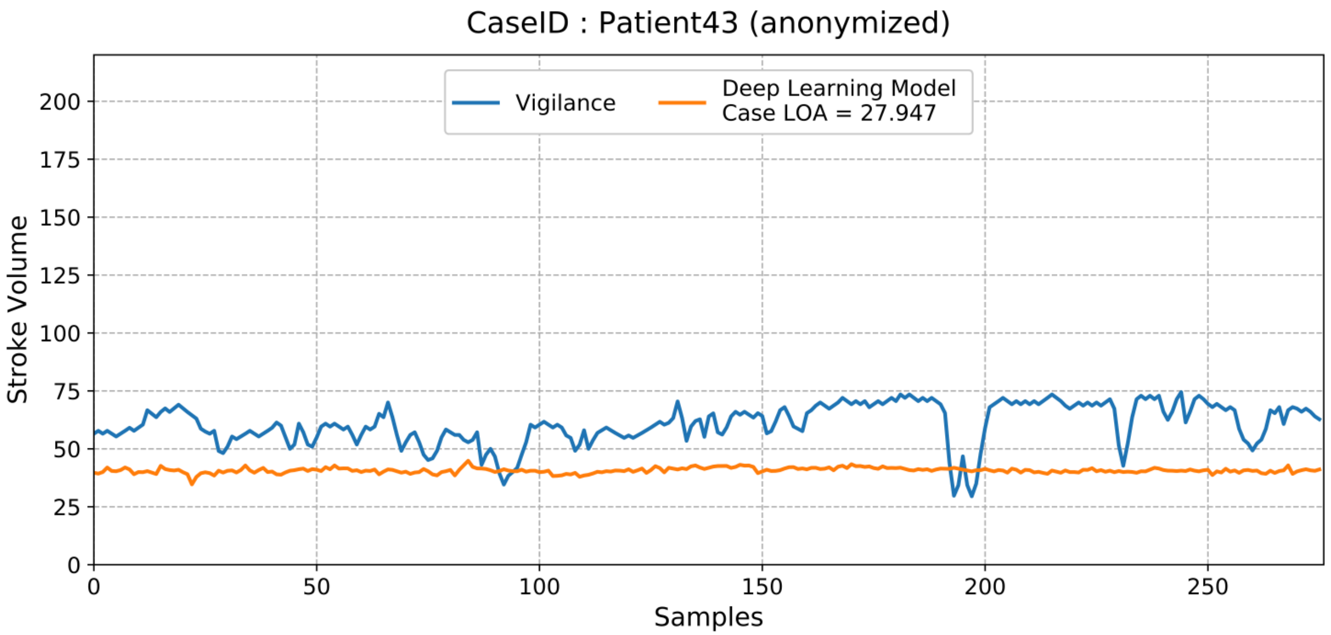

CaseID : Patient43 (anonymized)
Vigilance
Deep Learning Model
Case LOA = 27.947
Stroke Volume
Samples
0
25
50
75
100
125
150
175
200
250

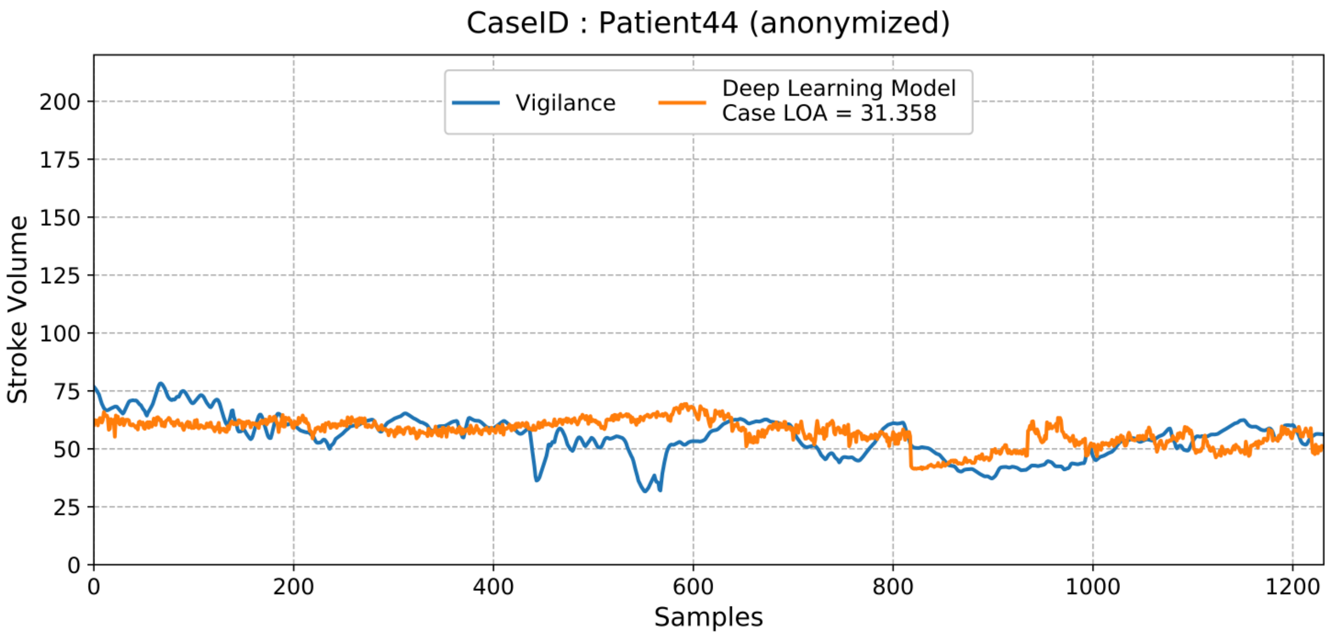
CaseID : Patient44 (anonymized)
Vigilance
Deep Learning Model
Case LOA = 31.358
Stroke Volume
Samples
0
25
50
75
100
125
150
175
200
0
200
400
600
800
1000
1200

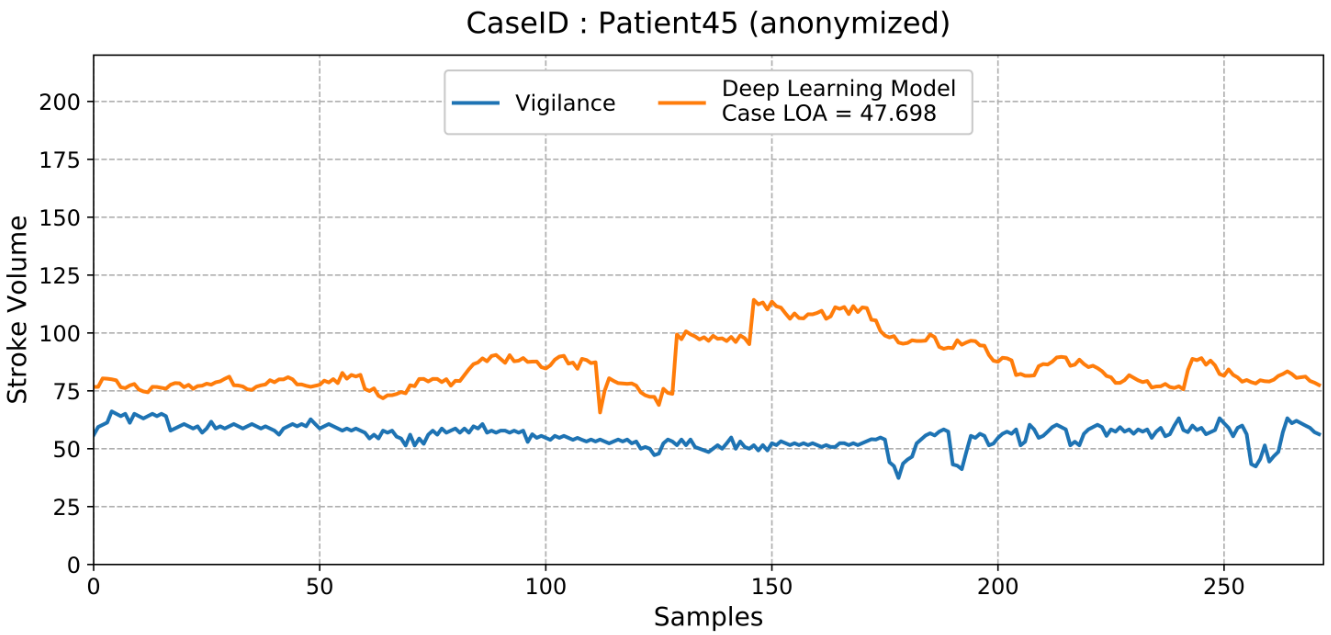
CaseID : Patient45 (anonymized)
Vigilance
Deep Learning Model
Case LOA = 47.698
Stroke Volume
Samples
0
25
50
75
100
125
150
175
200
0
50
100
150
200
250

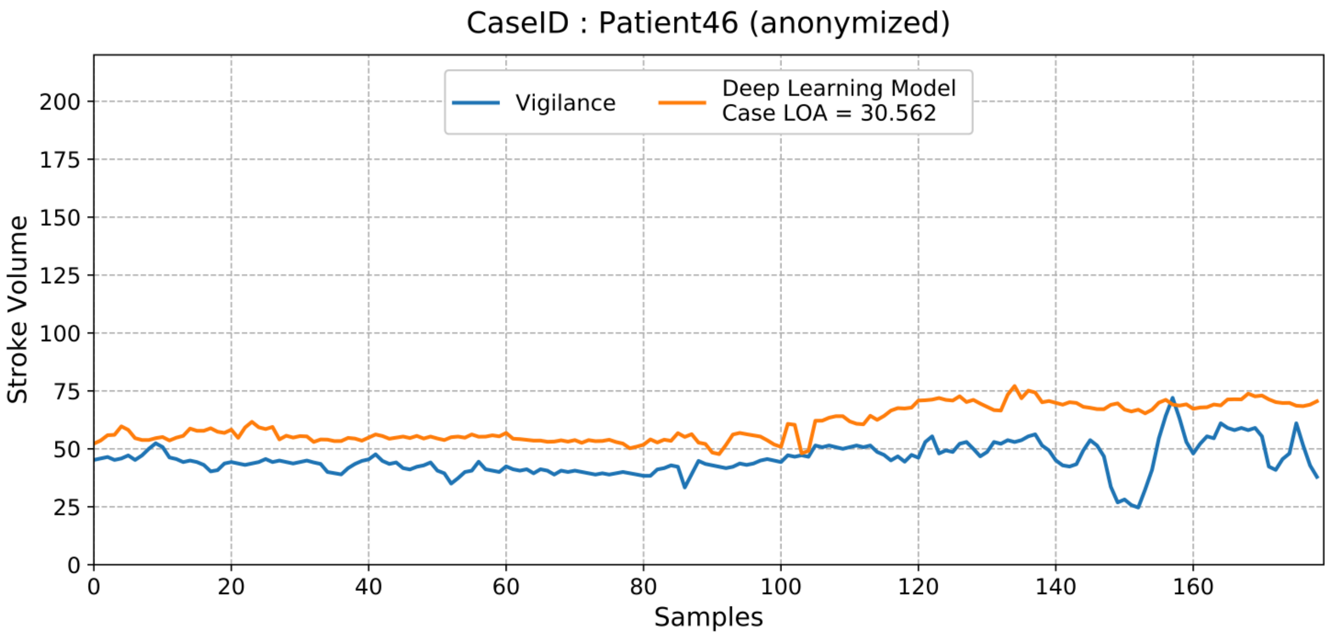

CaseID : Patient46 (anonymized)
Vigilance
Deep Learning Model
Case LOA = 30.562
Stroke Volume
Samples
0
25
50
75
100
125
150
175
200
0
20
40
60
80
100
120
140
160

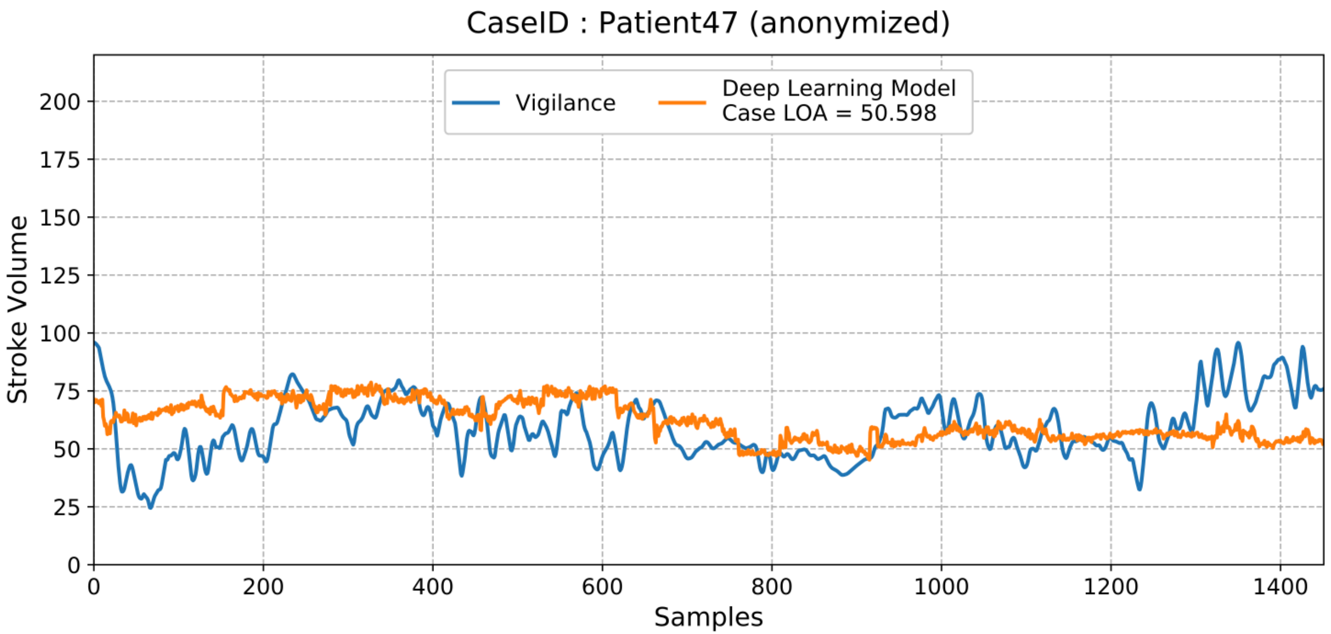

CaseID : Patient47 (anonymized)
Vigilance
Deep Learning Model
Case LOA = 50.598
Stroke Volume
Samples
0
25
50
75
100
125
150
175
200
0
200
400
600
800
1000
1200
1400

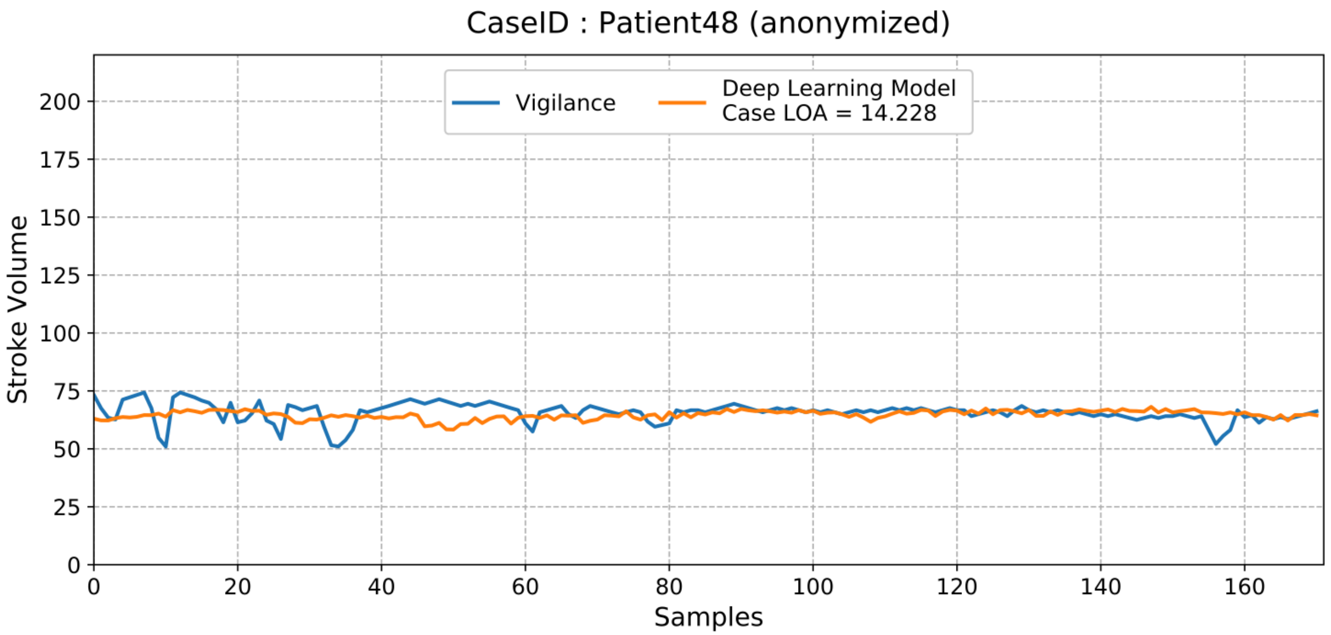

CaseID : Patient48 (anonymized)
Vigilance
Deep Learning Model
Case LOA = 14.228
Stroke Volume
Samples
0
25
50
75
100
125
150
175
200
0
20
40
60
80
100
120
140
160

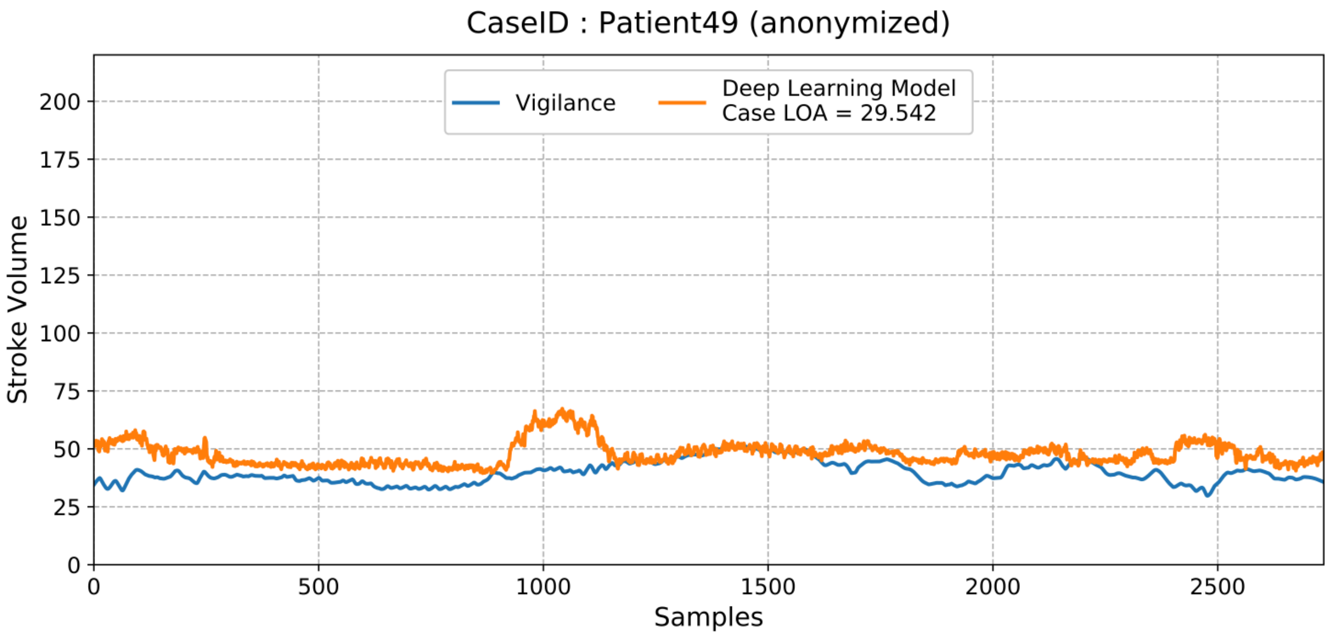
CaseID : Patient49 (anonymized)
Vigilance
Deep Learning Model
Case LOA = 29.542
Stroke Volume
Samples
0
25
50
75
100
125
150
175
200
500
1000
1500
2000
2500

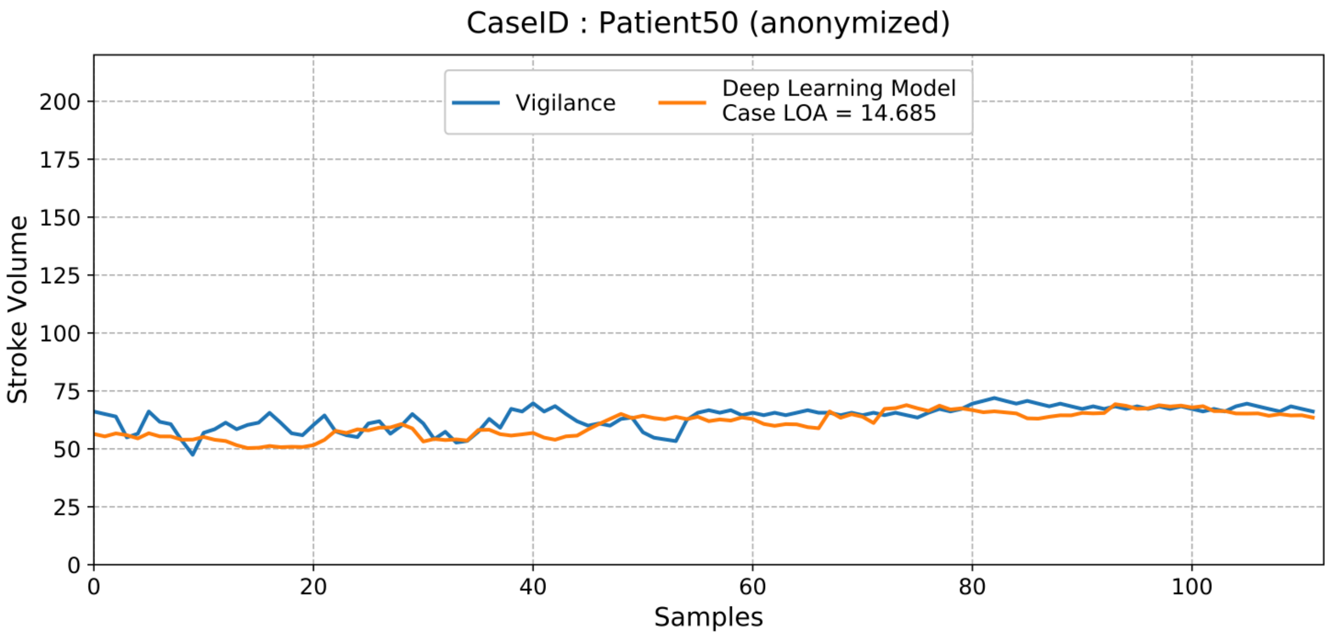

CaseID : Patient50 (anonymized)
Vigilance
Deep Learning Model
Case LOA = 14.685
Stroke Volume
Samples
0
25
50
75
100
125
150
175
200
0
20
40
60
80
100

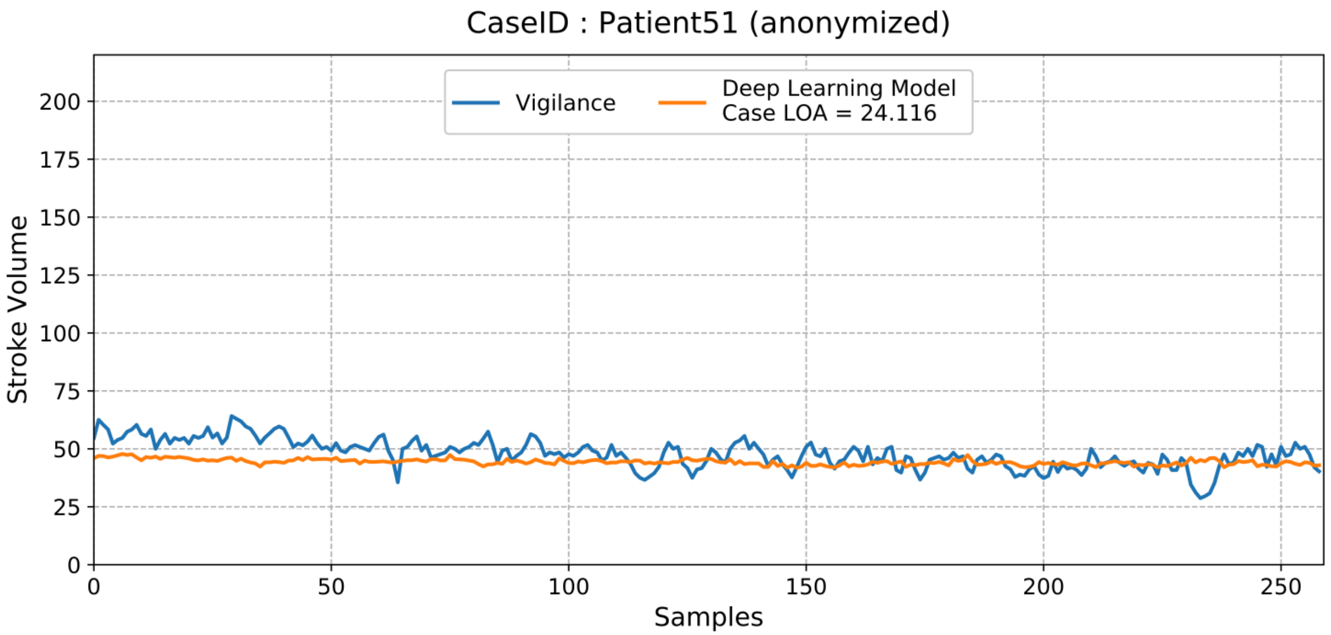

CaseID : Patient51 (anonymized)
Vigilance
Deep Learning Model
Case LOA = 24.116
Stroke Volume
Samples
0
25
50
75
100
125
150
175
200
250

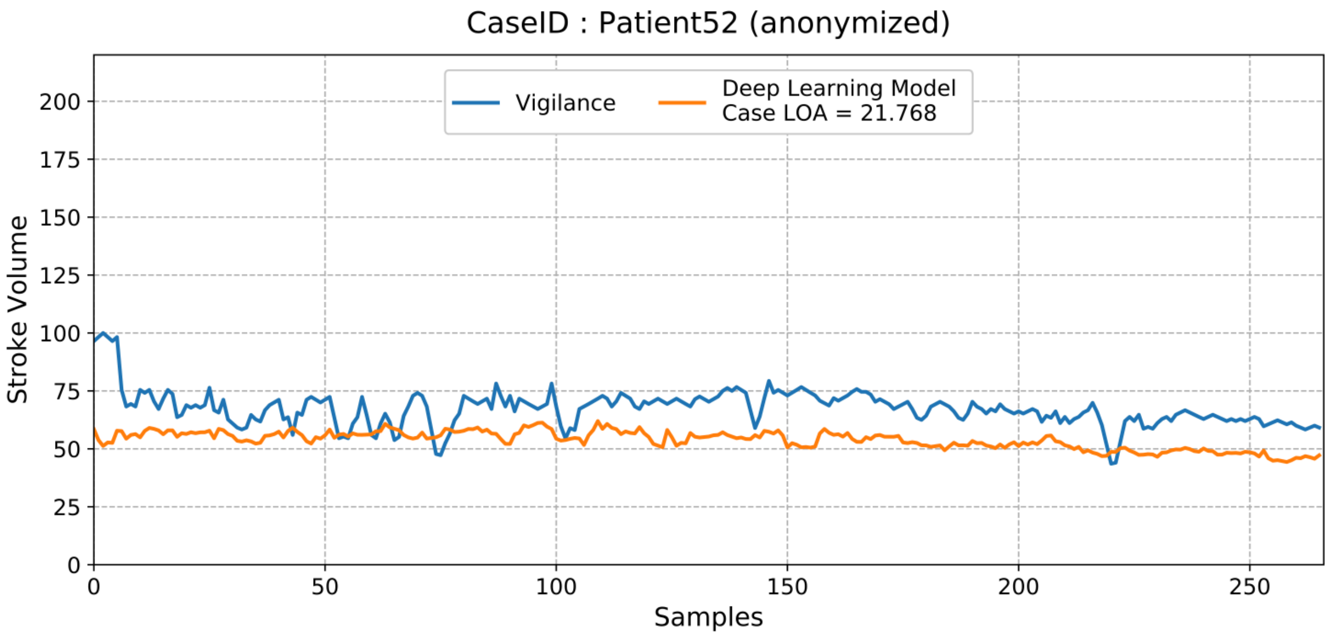

CaseID : Patient52 (anonymized)
Vigilance
Deep Learning Model
Case LOA = 21.768
Stroke Volume
Samples
0
25
50
75
100
125
150
175
200
0
50
100
150
200
250

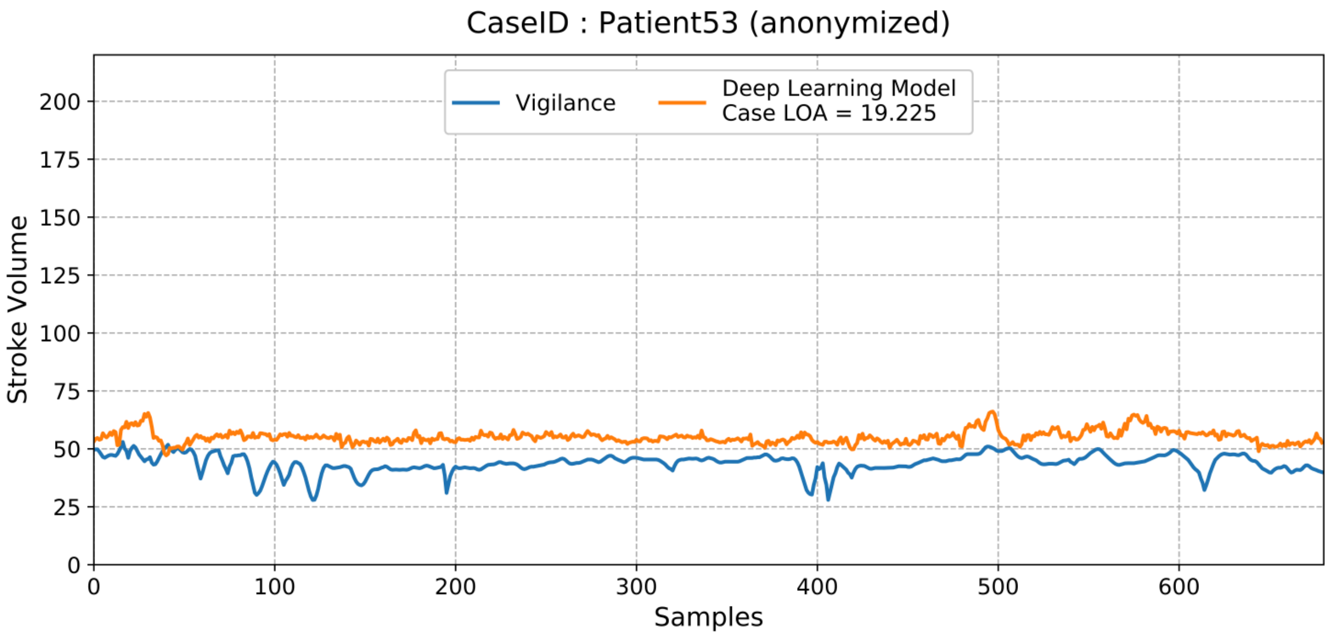
CaseID : Patient53 (anonymized)
Vigilance
Deep Learning Model
Case LOA = 19.225
Stroke Volume
Samples
0
25
50
75
100
125
150
175
200
0
100
200
300
400
500
600

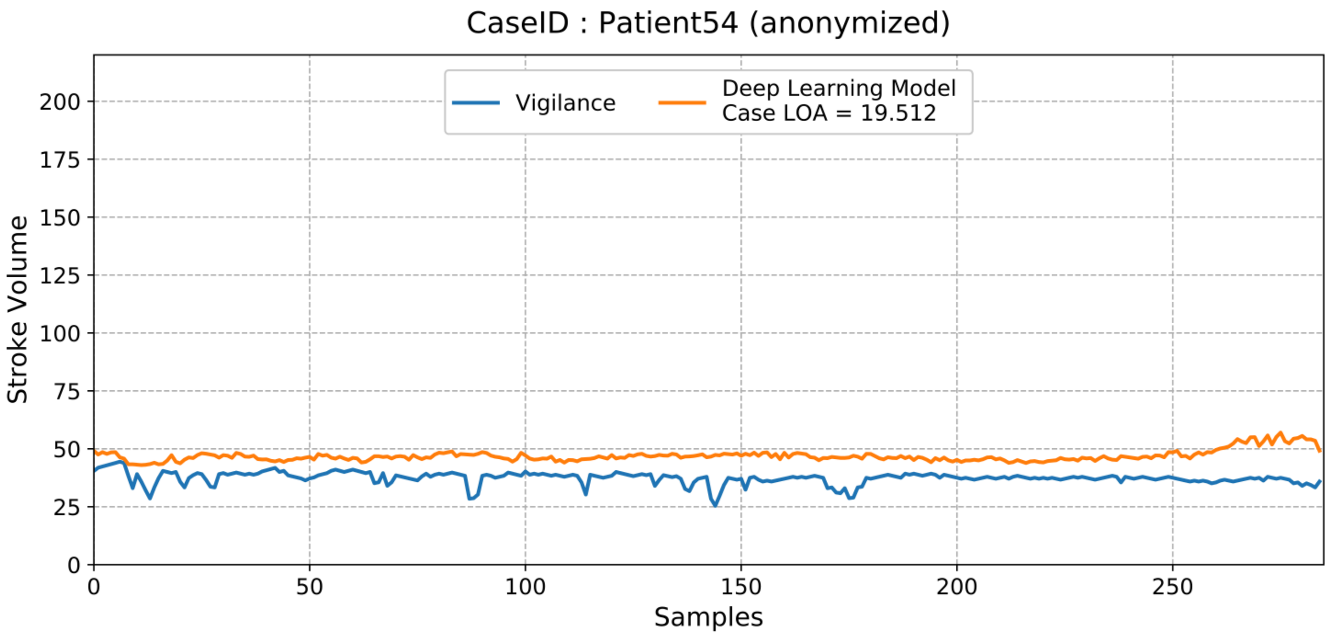

CaseID : Patient54 (anonymized)
Vigilance
Deep Learning Model
Case LOA = 19.512
Stroke Volume
Samples
0
25
50
75
100
125
150
175
200
250

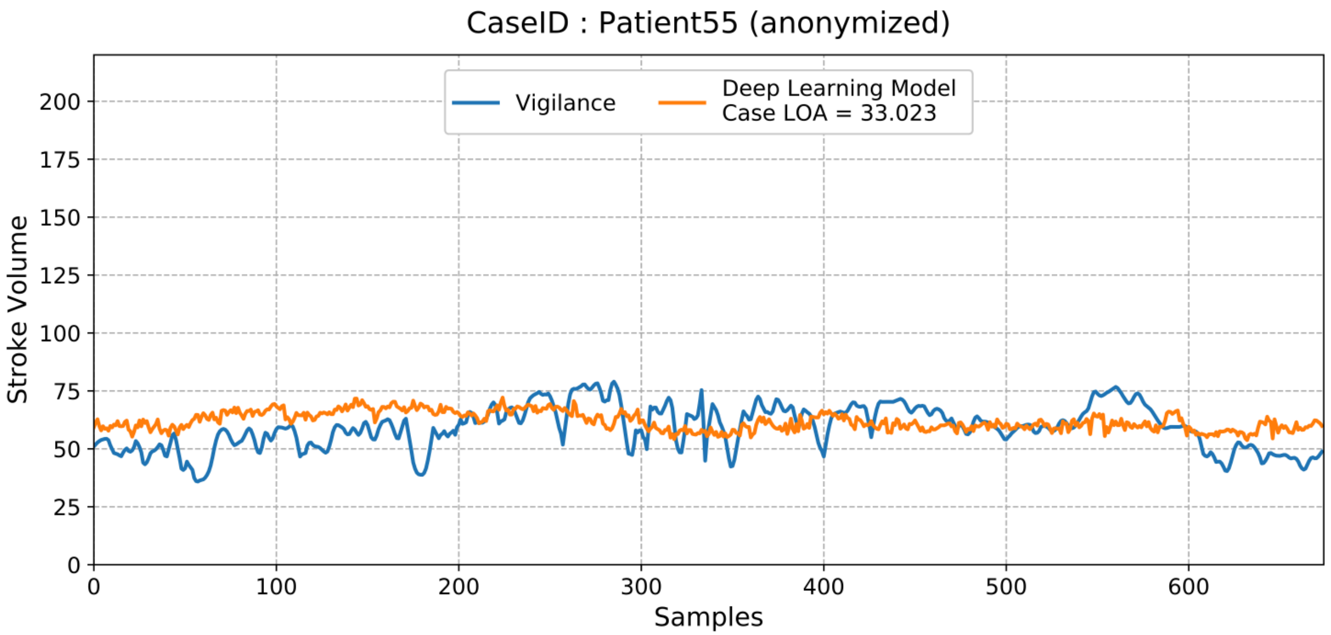
CaseID : Patient55 (anonymized)
Vigilance
Deep Learning Model
Case LOA = 33.023
Stroke Volume
Samples
0
25
50
75
100
125
150
175
200
0
100
200
300
400
500
600

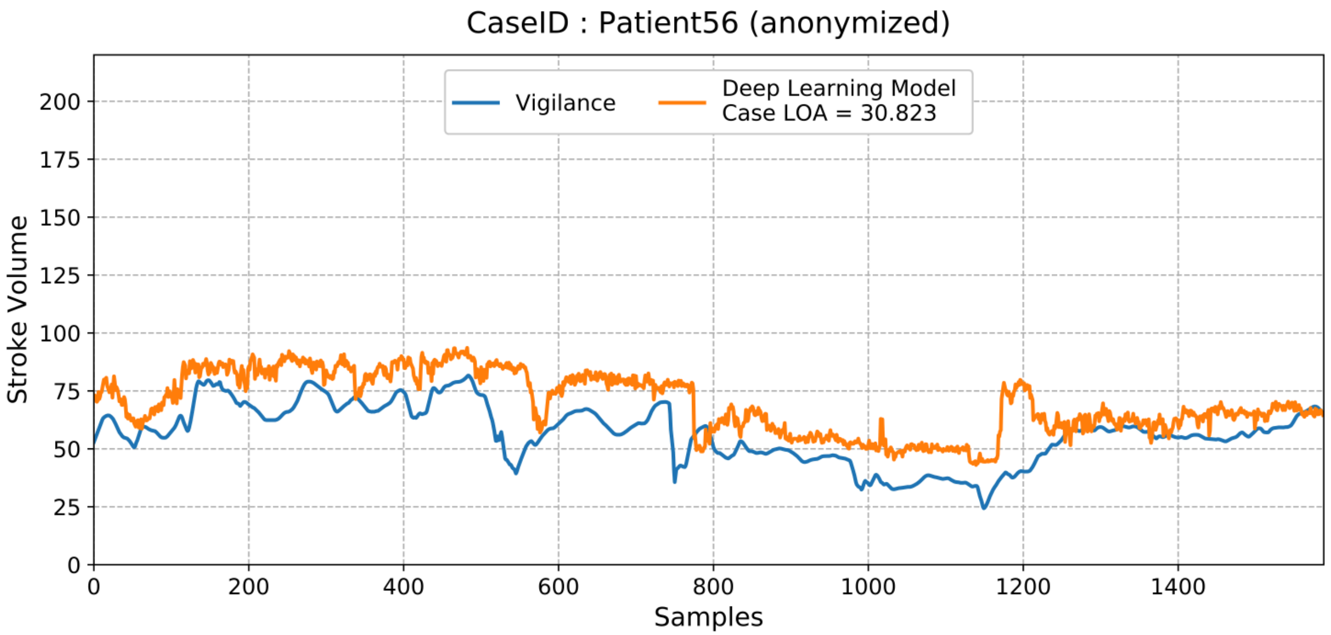
CaseID : Patient56 (anonymized)
Vigilance
Deep Learning Model
Case LOA = 30.823
Stroke Volume
Samples
0
25
50
75
100
125
150
175
200
0
200
400
600
800
1000
1200
1400

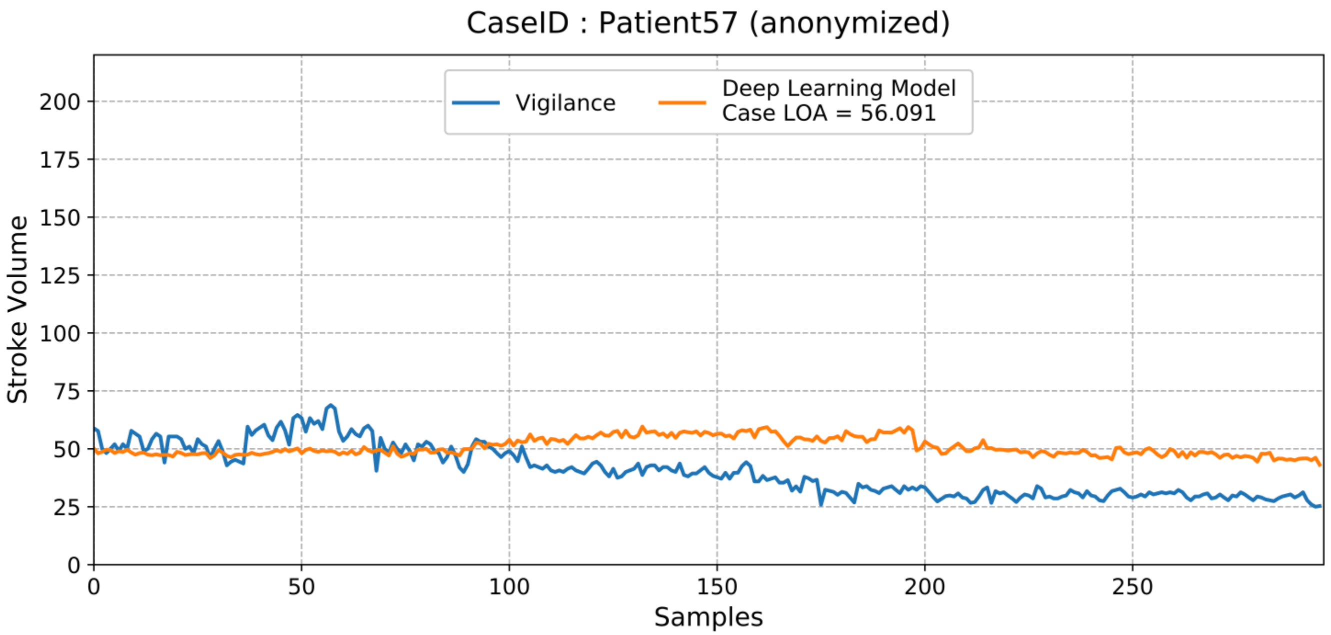

CaseID : Patient57 (anonymized)
Vigilance
Deep Learning Model
Case LOA = 56.091
Stroke Volume
Samples
0
25
50
75
100
125
150
175
200
0
50
100
150
200
250

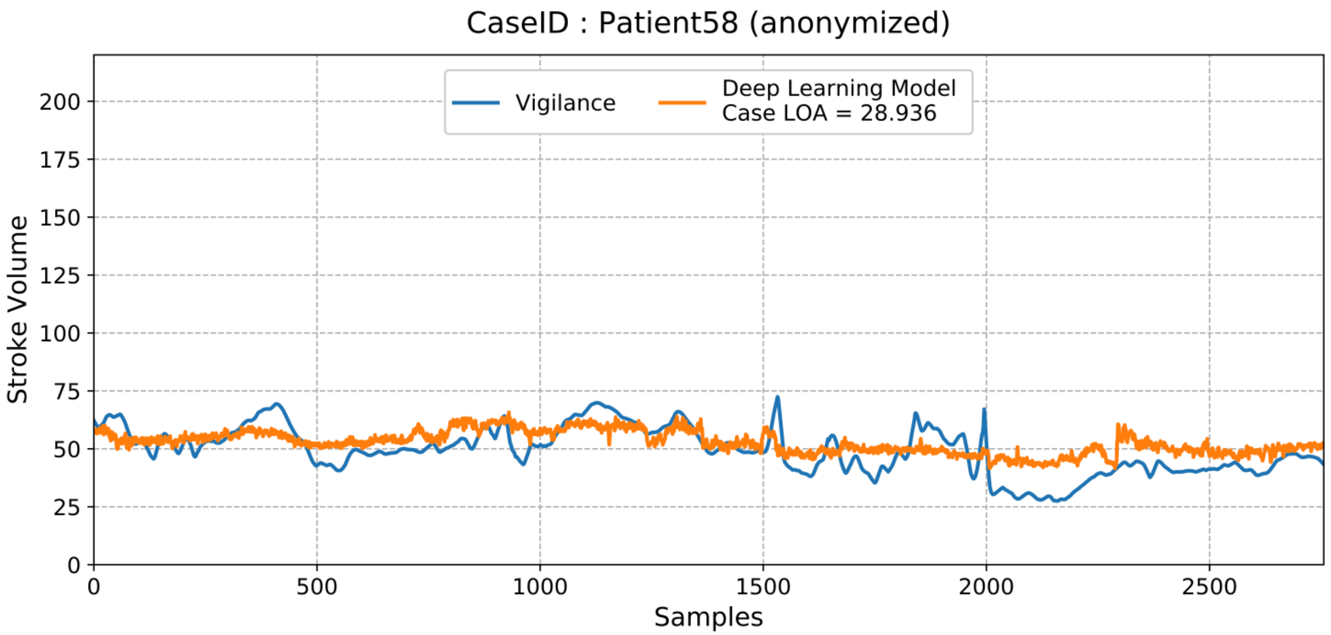

CaseID : Patient58 (anonymized)
Vigilance
Deep Learning Model
Case LOA = 28.936
Stroke Volume
Samples
0
25
50
75
100
125
150
175
200
0
500
1000
1500
2000
2500

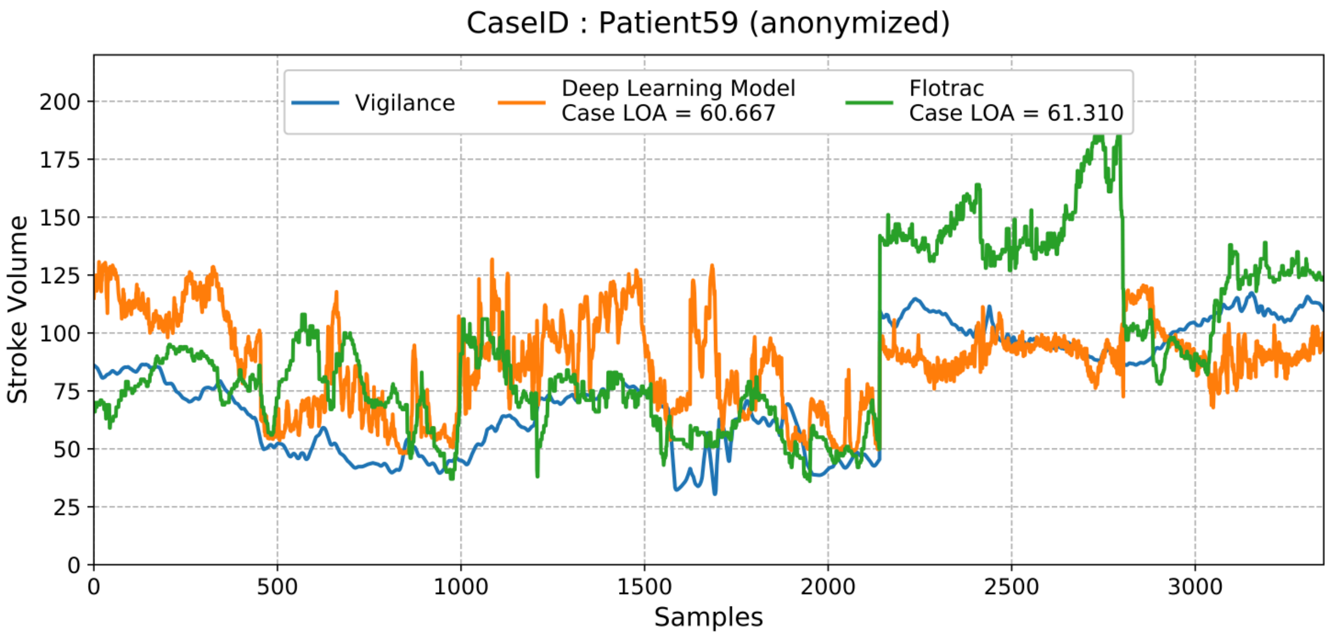

CaseID : Patient59 (anonymized)
Vigilance
Deep Learning Model
Case LOA = 60.667
Flotrac
Case LOA = 61.310
Stroke Volume
Samples
0
25
50
75
100
125
150
175
200
0
500
1000
1500
2000
2500
3000

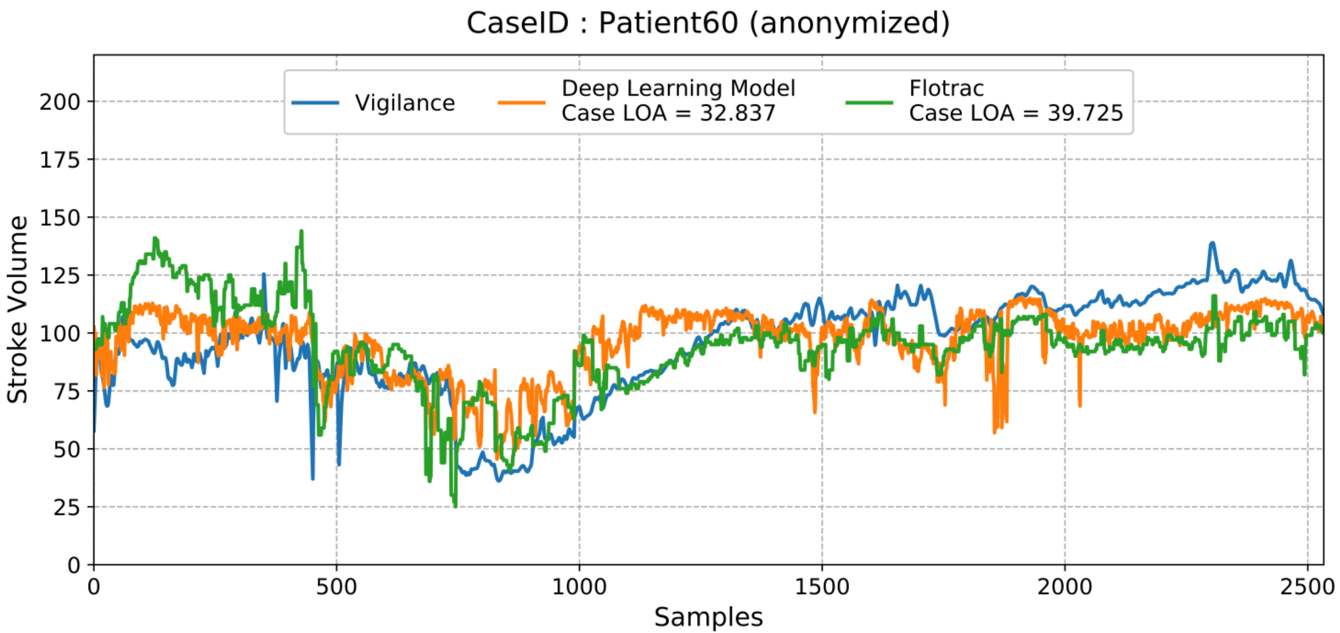
CaseID : Patient60 (anonymized)
Vigilance
Deep Learning Model
Case LOA = 32.837
Flotrac
Case LOA = 39.725
Stroke Volume
200
175
150
125
100
75
50
25
0
0
500
1000
1500
2000
2500
Samples

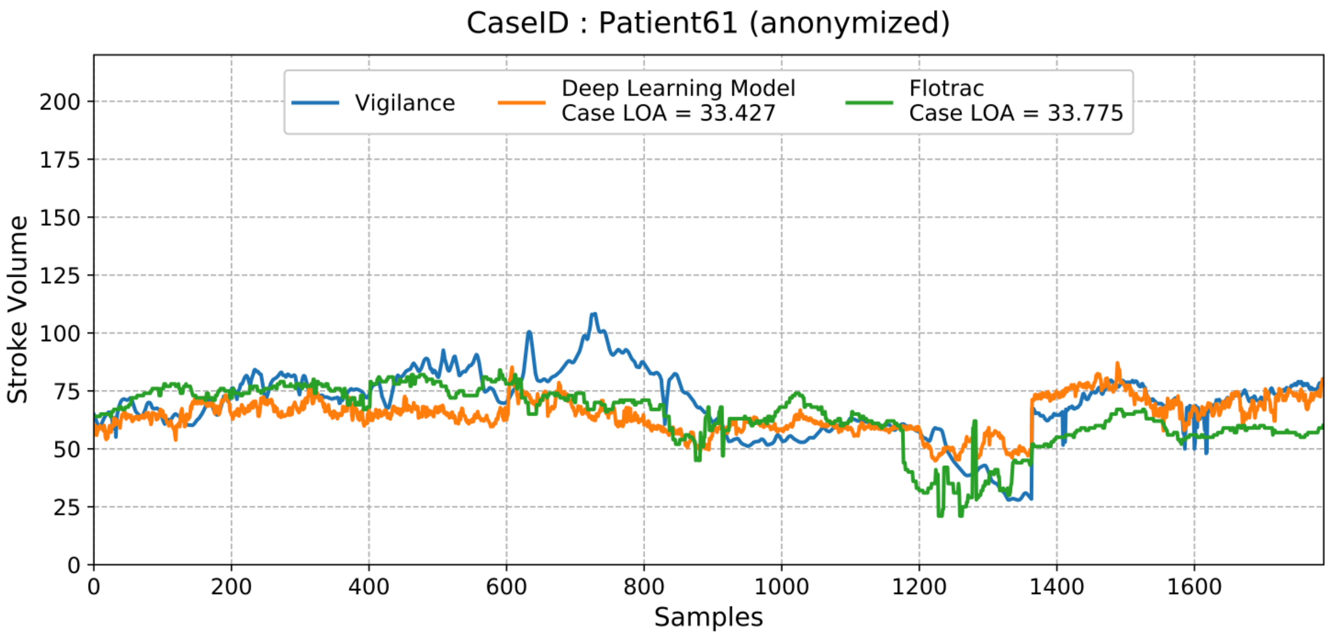

CaseID : Patient61 (anonymized)
Vigilance
Deep Learning Model
Case LOA = 33.427
Flotrac
Case LOA = 33.775
Stroke Volume
0
25
50
75
100
125
150
175
200
Samples
0
200
400
600
800
1000
1200
1400
1600

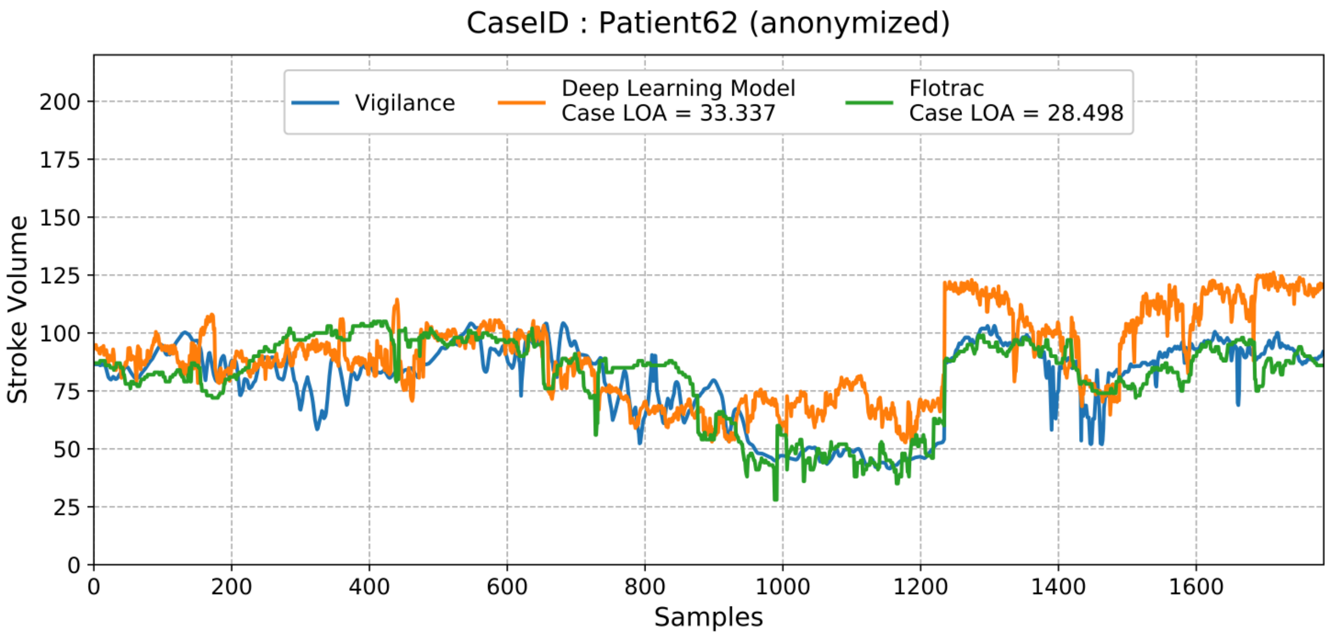

CaseID : Patient62 (anonymized)
Vigilance
Deep Learning Model
Case LOA = 33.337
Flotrac
Case LOA = 28.498
Stroke Volume
Samples
0
25
50
75
100
125
150
175
200
0
200
400
600
800
1000
1200
1400
1600

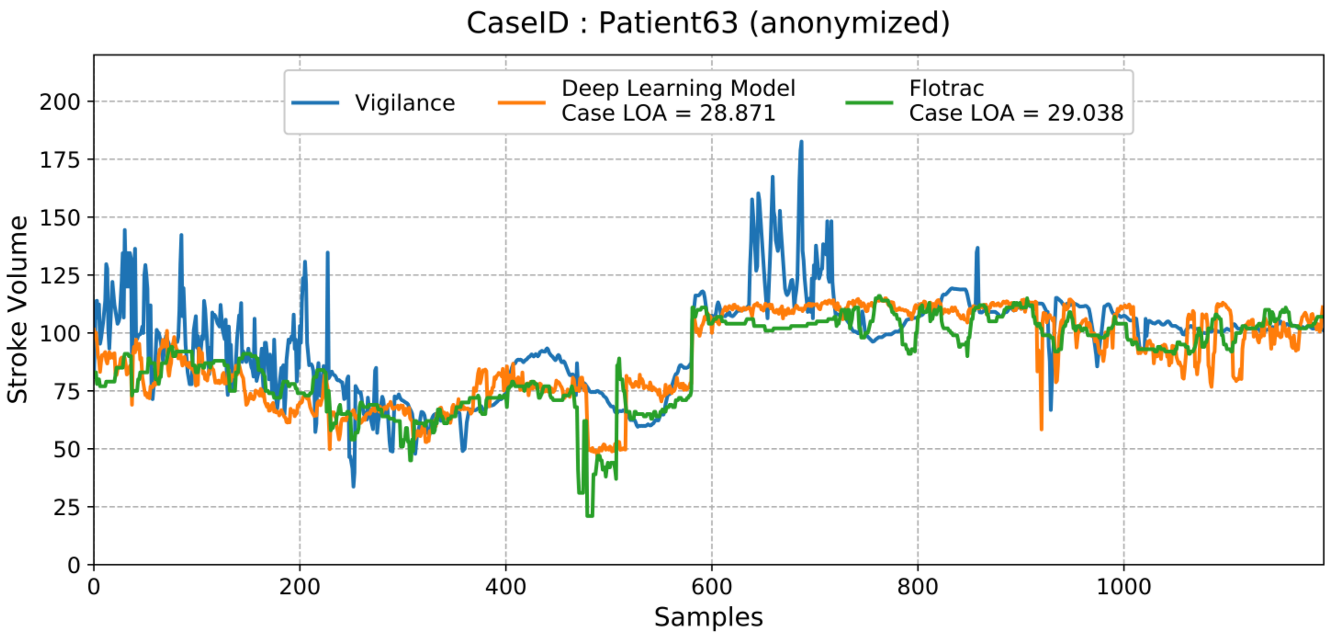

CaseID : Patient63 (anonymized)
Vigilance
Deep Learning Model
Case LOA = 28.871
Flotrac
Case LOA = 29.038
Stroke Volume
Samples
0
25
50
75
100
125
150
175
200
0
200
400
600
800
1000

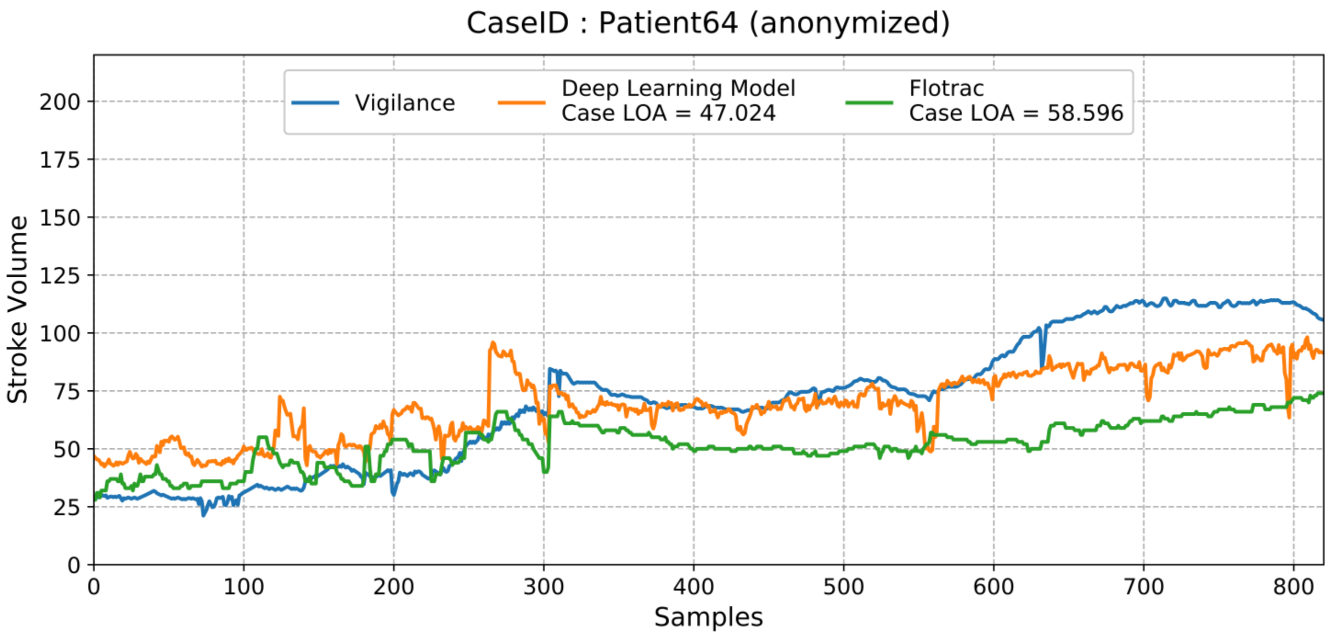

CaseID : Patient64 (anonymized)
Vigilance
Deep Learning Model
Case LOA = 47.024
Flotrac
Case LOA = 58.596
Stroke Volume
Samples
0
25
50
75
100
125
150
175
200
0
100
200
300
400
500
600
700
800

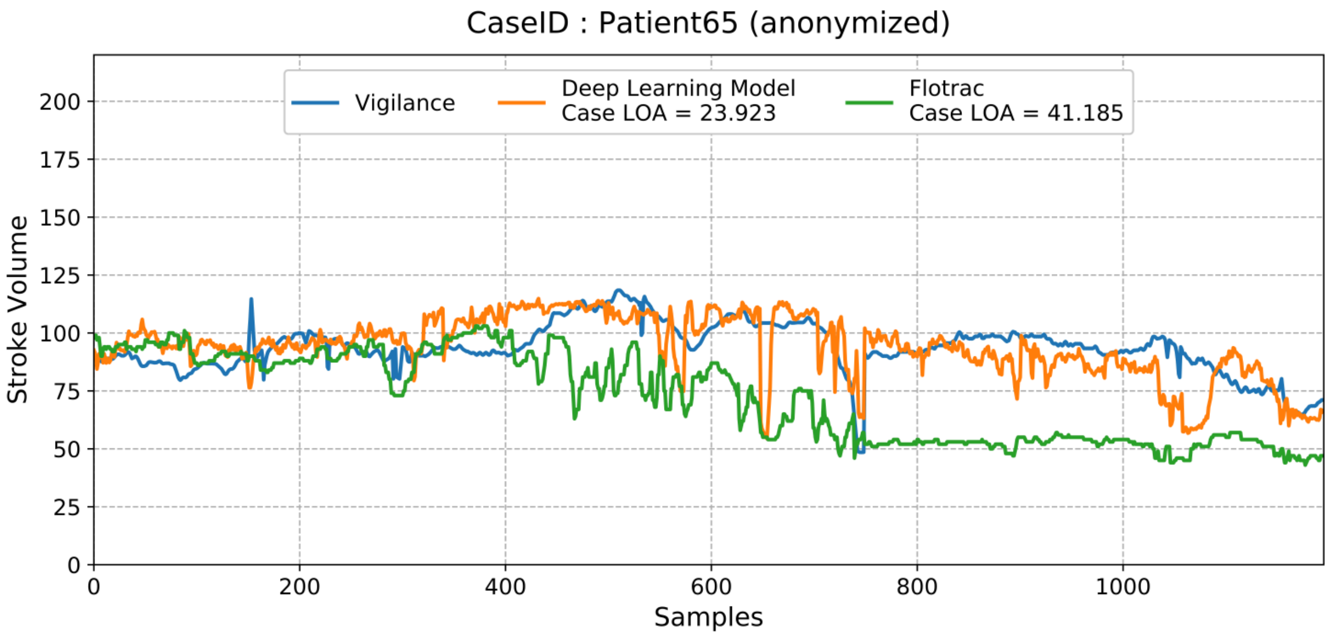

CaseID : Patient65 (anonymized)
Vigilance
Deep Learning Model
Case LOA = 23.923
Flotrac
Case LOA = 41.185
Stroke Volume
Samples
0
25
50
75
100
125
150
175
200
0
200
400
600
800
1000

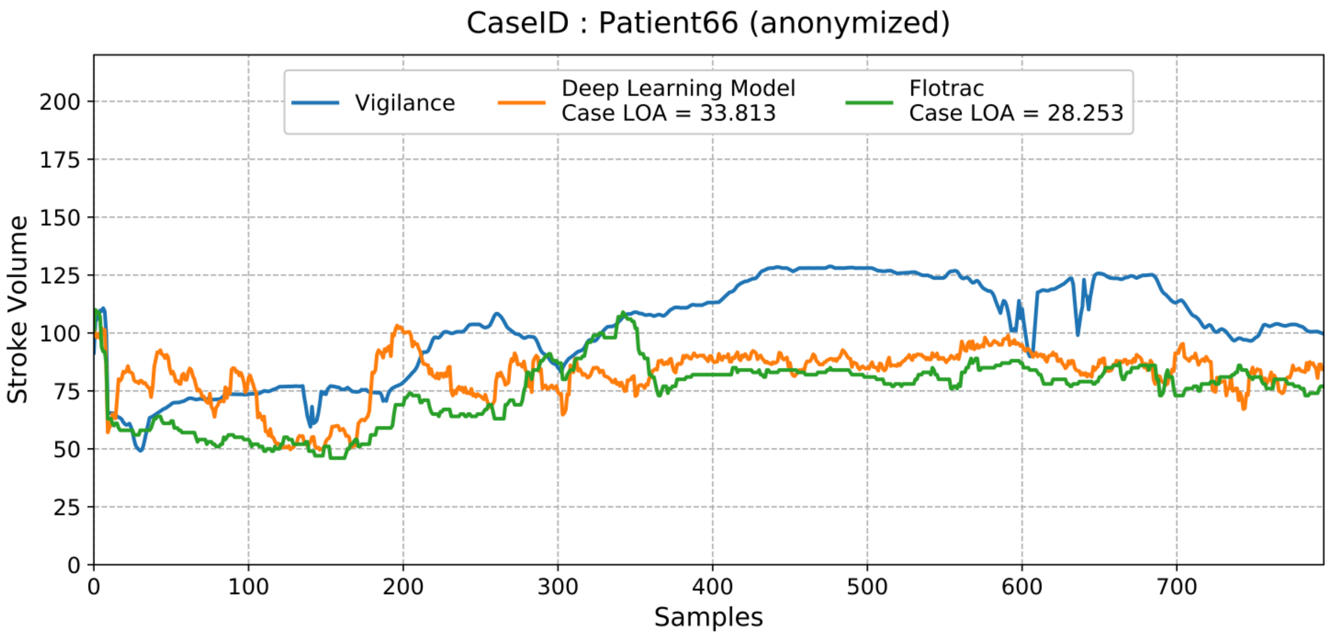
CaseID : Patient66 (anonymized)
Vigilance
Deep Learning Model
Case LOA = 33.813
Flotrac
Case LOA = 28.253
Stroke Volume
Samples
0
25
50
75
100
125
150
175
200
0
100
200
300
400
500
600
700

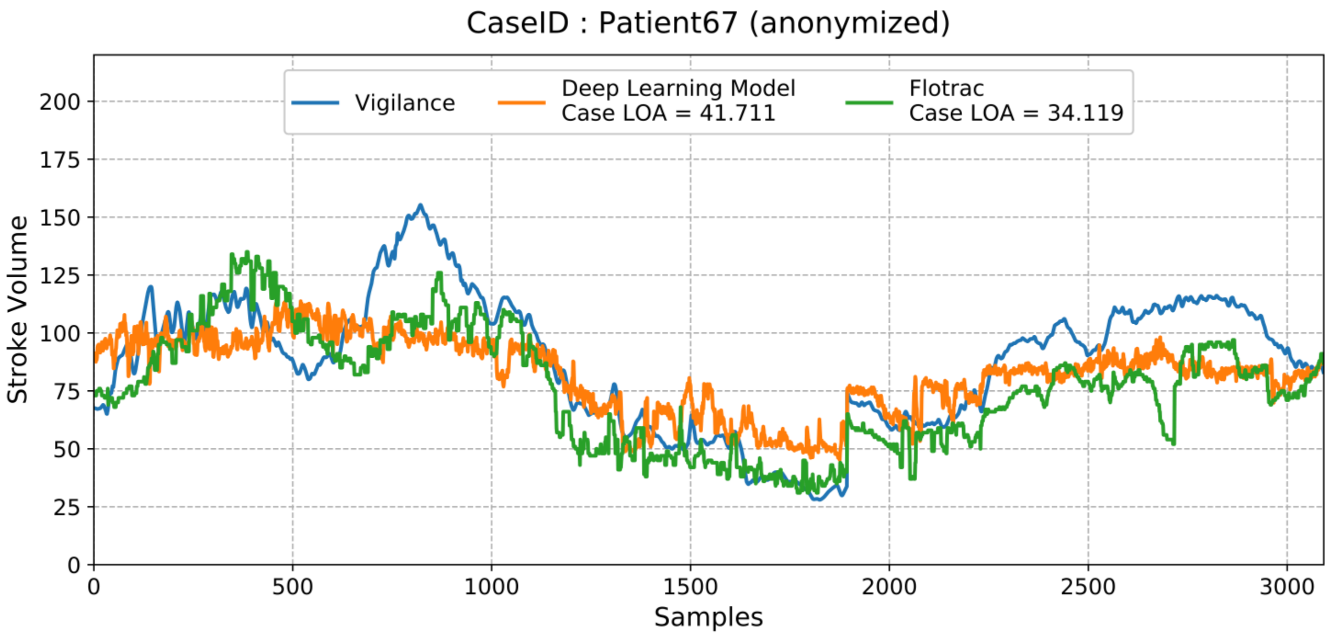

CaseID : Patient67 (anonymized)
Vigilance
Deep Learning Model
Case LOA = 41.711
Flotrac
Case LOA = 34.119
Stroke Volume
Samples
0
25
50
75
100
125
150
175
200
0
500
1000
1500
2000
2500
3000

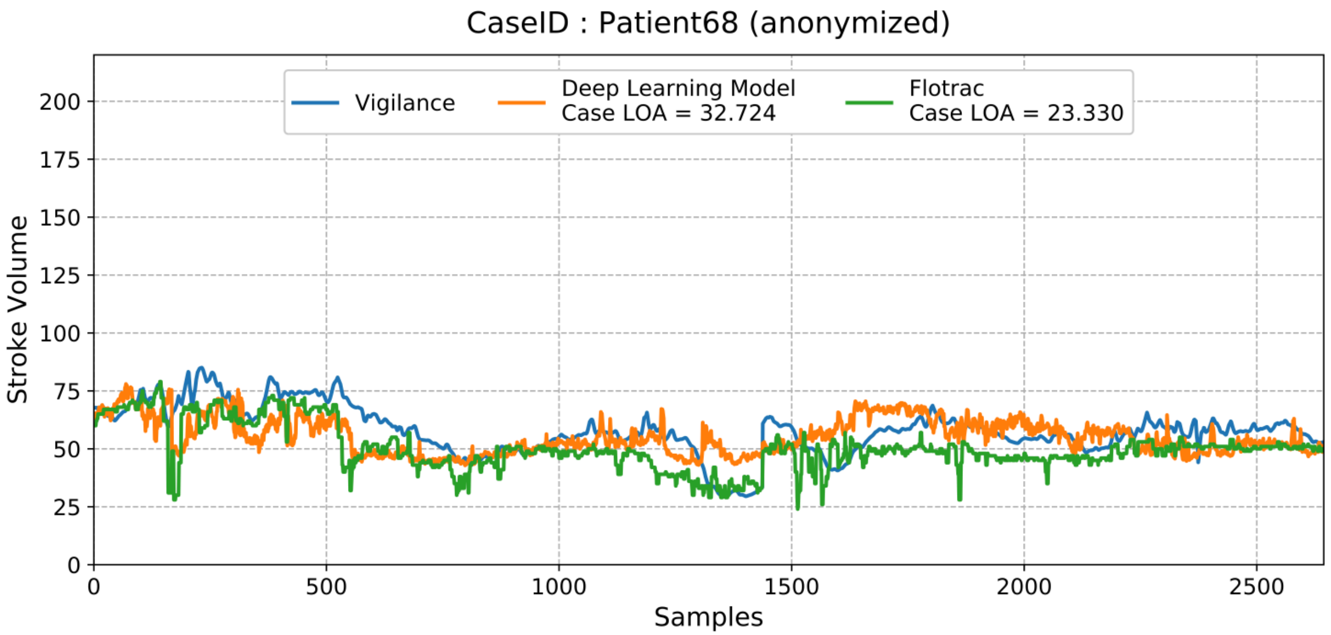

CaseID : Patient68 (anonymized)
Vigilance
Deep Learning Model
Case LOA = 32.724
Flotrac
Case LOA = 23.330
Stroke Volume
Samples
0
25
50
75
100
125
150
175
200
0
500
1000
1500
2000
2500

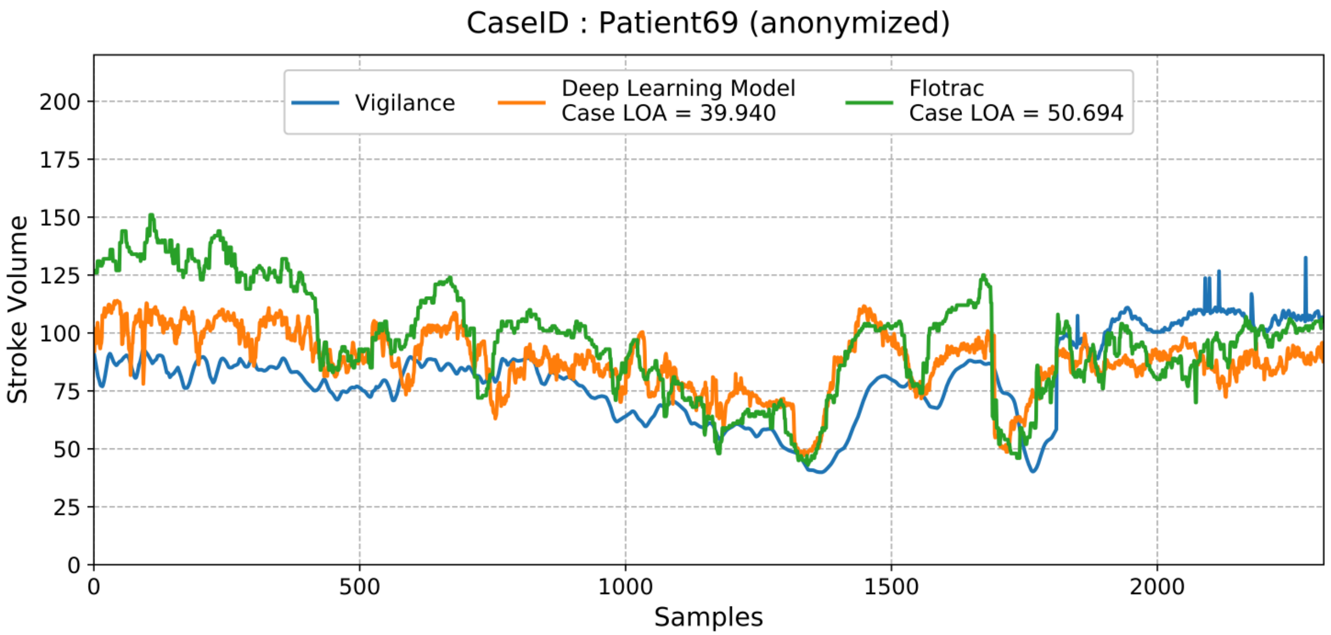

CaseID : Patient69 (anonymized)
Vigilance
Deep Learning Model
Case LOA = 39.940
Flotrac
Case LOA = 50.694
Stroke Volume
Samples
0
25
50
75
100
125
150
175
200
0
500
1000
1500
2000

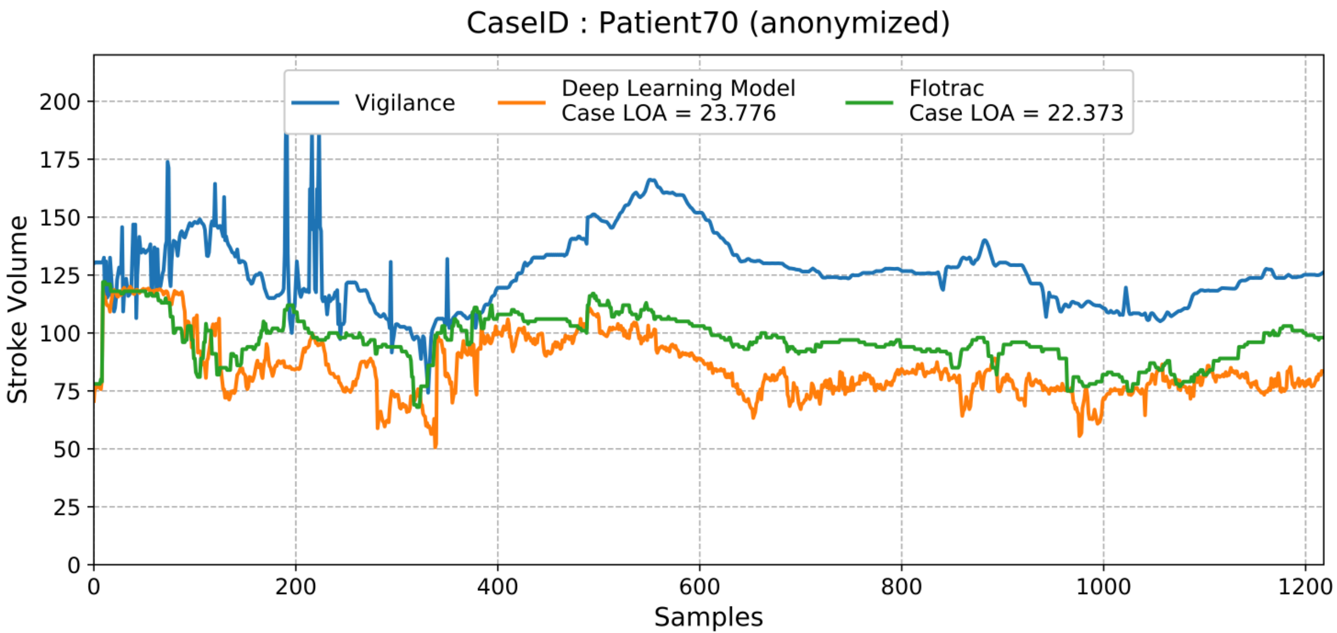

CaseID : Patient70 (anonymized)
Vigilance
Deep Learning Model
Case LOA = 23.776
Flotrac
Case LOA = 22.373
Stroke Volume
Samples
0
25
50
75
100
125
150
175
200
0
200
400
600
800
1000
1200

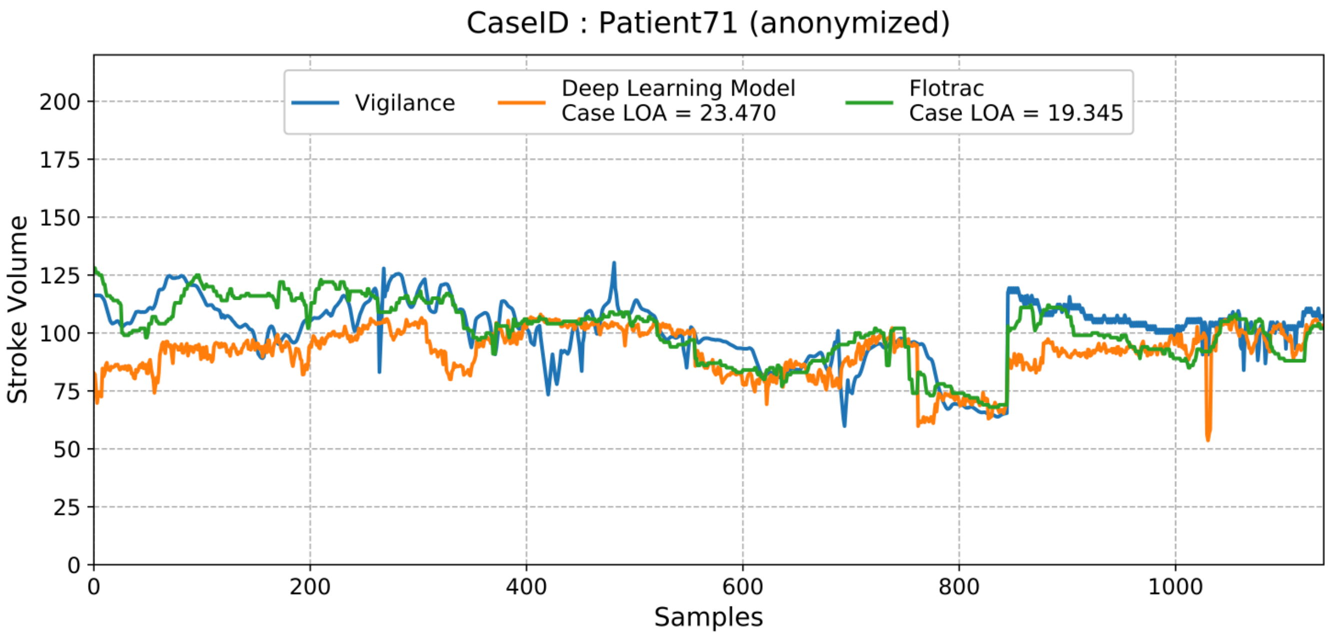

CaseID : Patient71 (anonymized)
Vigilance
Deep Learning Model
Case LOA = 23.470
Flotrac
Case LOA = 19.345
Stroke Volume
Samples
0
25
50
75
100
125
150
175
200
0
200
400
600
800
1000

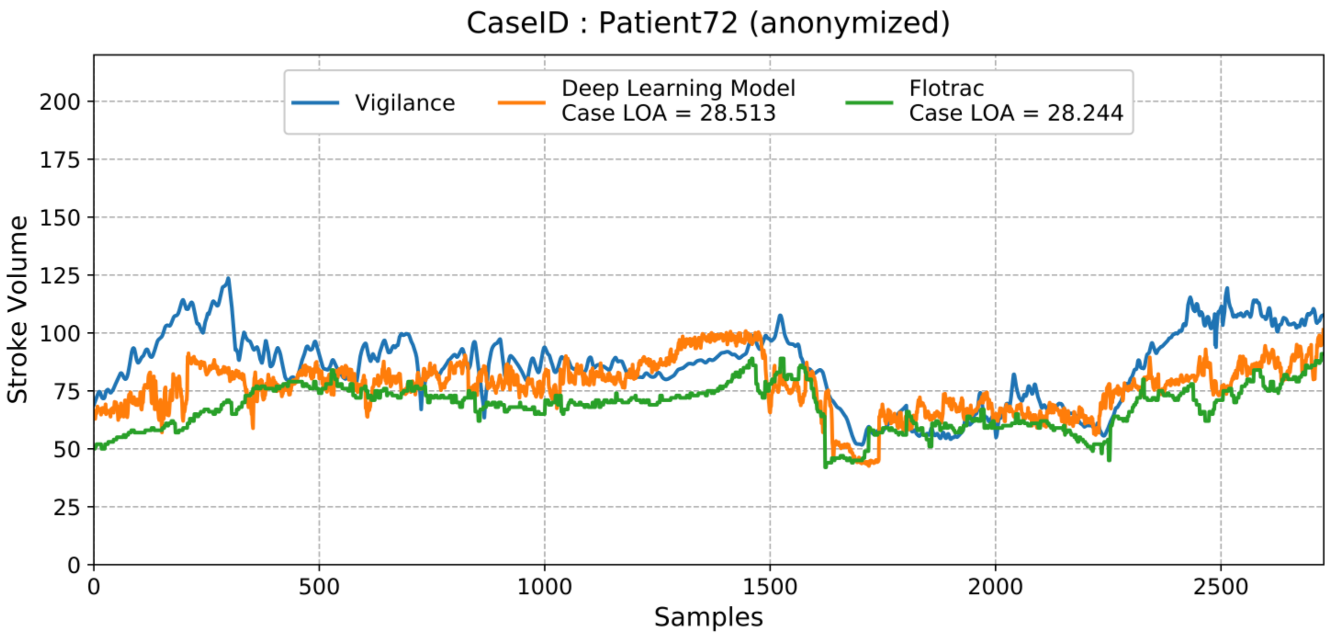

CaseID : Patient72 (anonymized)
Vigilance
Deep Learning Model
Case LOA = 28.513
Flotrac
Case LOA = 28.244
Stroke Volume
Samples
0
25
50
75
100
125
150
175
200
0
500
1000
1500
2000
2500

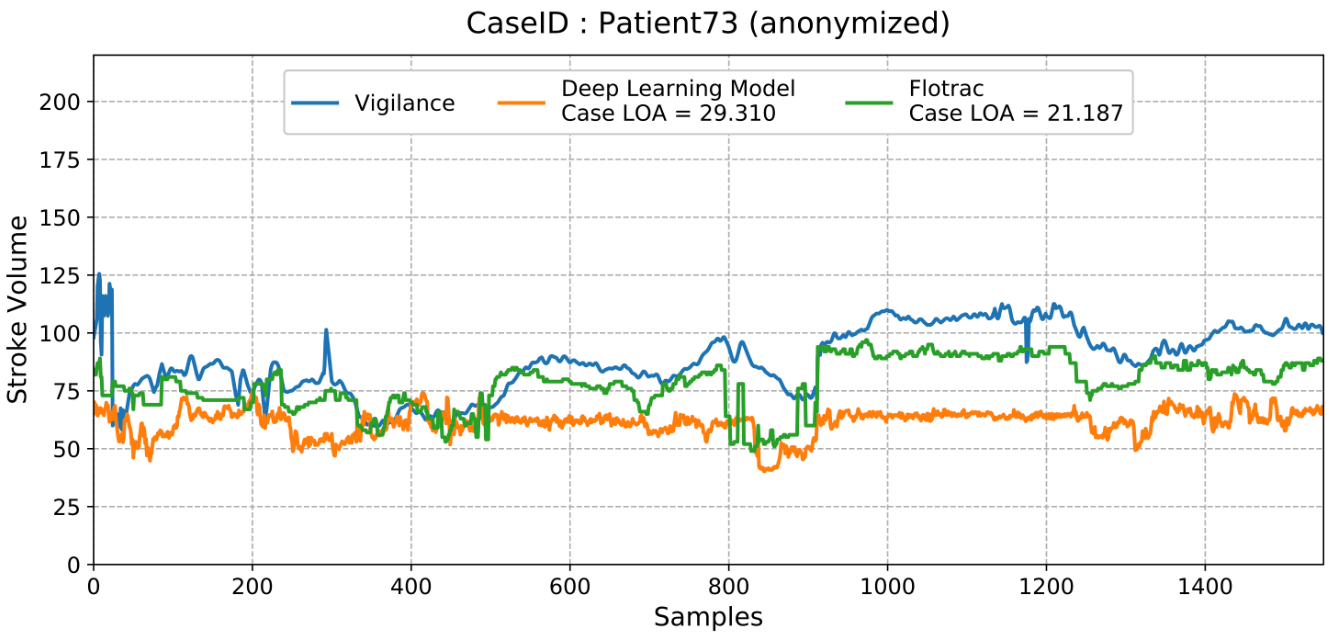
CaseID : Patient73 (anonymized)
Vigilance
Deep Learning Model
Case LOA = 29.310
Flotrac
Case LOA = 21.187
Stroke Volume
Samples
0
25
50
75
100
125
150
175
200
0
200
400
600
800
1000
1200
1400

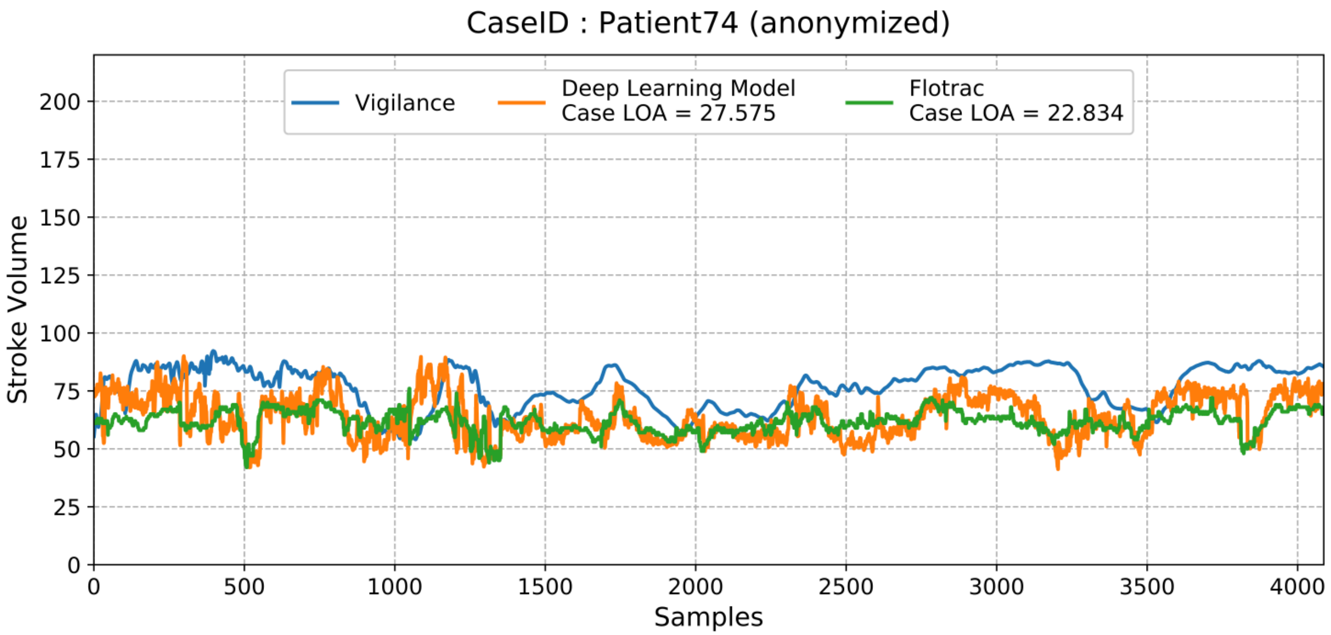

CaseID : Patient74 (anonymized)
Vigilance
Deep Learning Model
Case LOA = 27.575
Flotrac
Case LOA = 22.834
Stroke Volume
Samples
0
25
50
75
100
125
150
175
200
0
500
1000
1500
2000
2500
3000
3500
4000

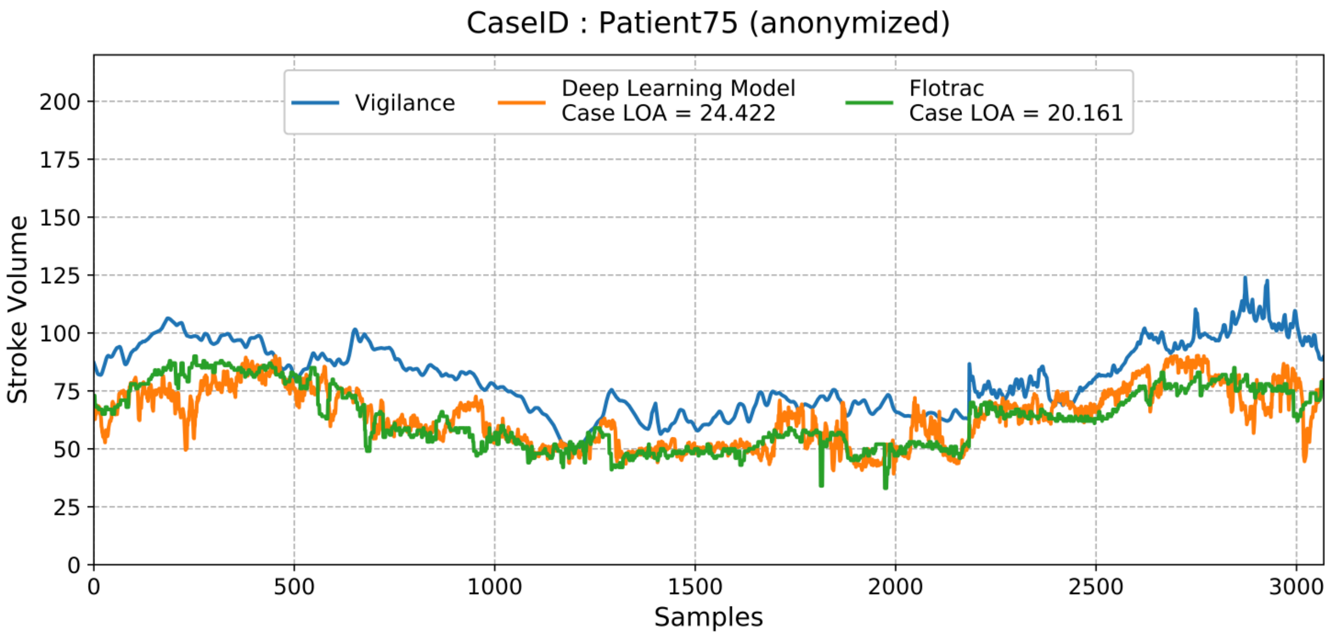

CaseID : Patient75 (anonymized)
Vigilance
Deep Learning Model
Case LOA = 24.422
Flotrac
Case LOA = 20.161
Stroke Volume
Samples
0
25
50
75
100
125
150
175
200
0
500
1000
1500
2000
2500
3000

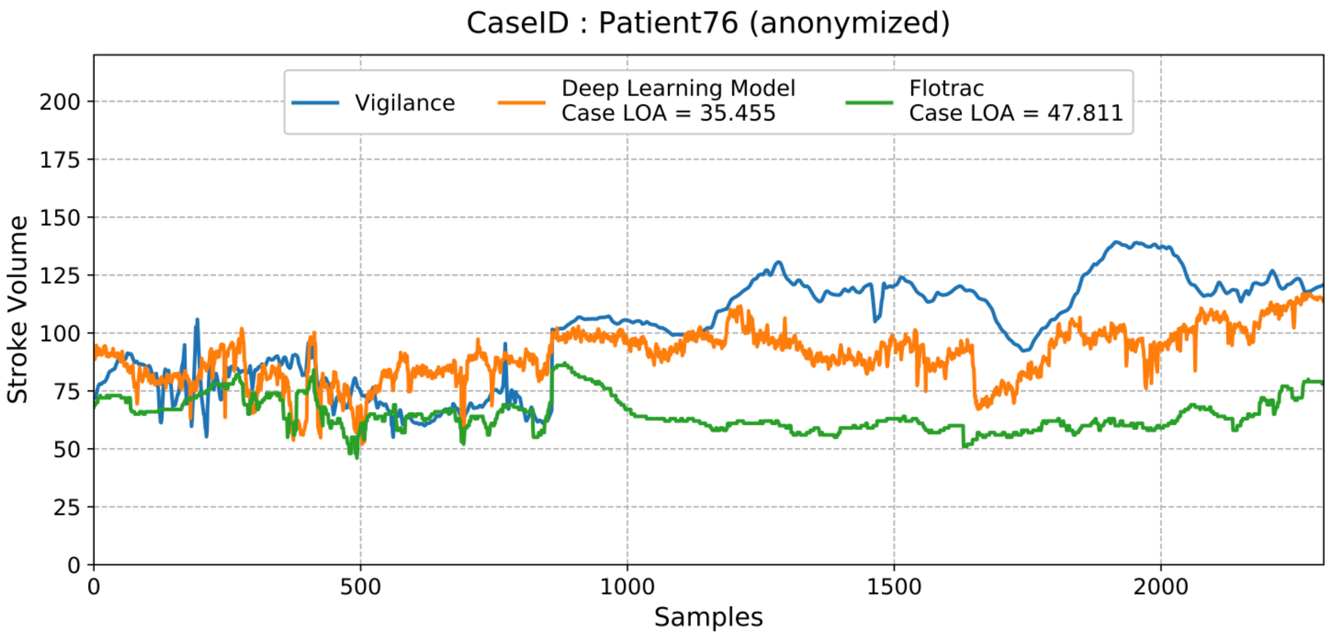
CaseID : Patient76 (anonymized)
Vigilance
Deep Learning Model
Case LOA = 35.455
Flotrac
Case LOA = 47.811
Stroke Volume
Samples
0
25
50
75
100
125
150
175
200
0
500
1000
1500
2000

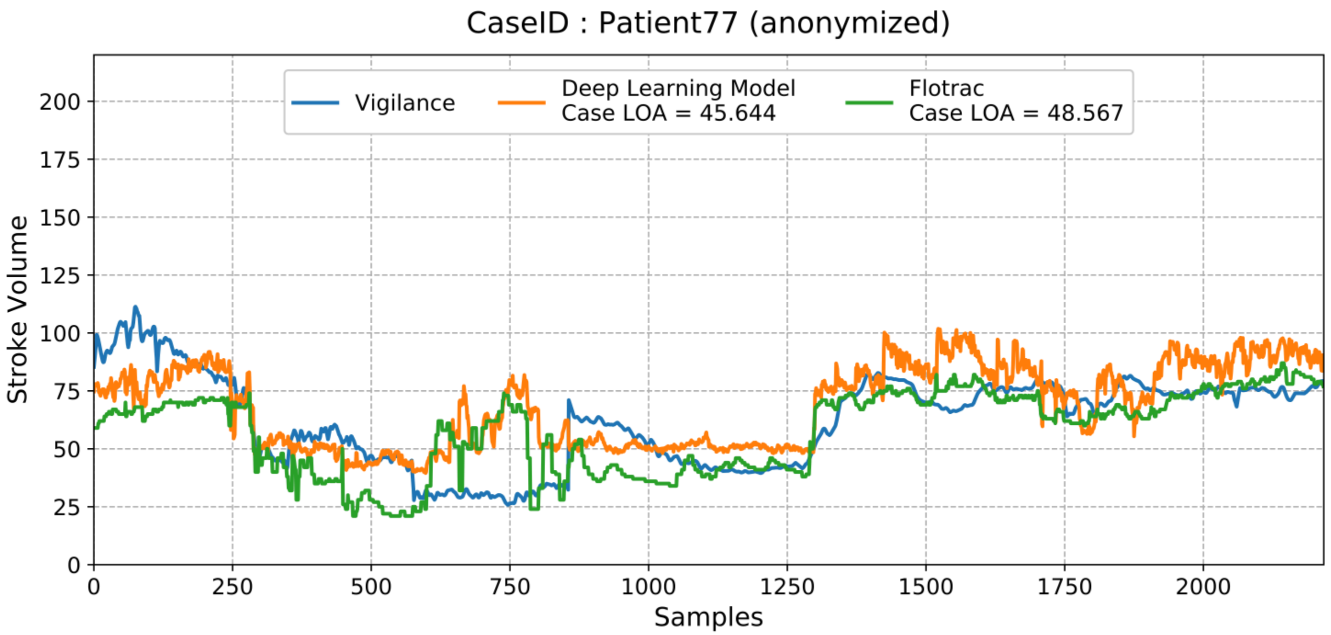
CaseID : Patient77 (anonymized)
Vigilance
Deep Learning Model
Case LOA = 45.644
Flotrac
Case LOA = 48.567
Stroke Volume
Samples
0
25
50
75
100
125
150
175
200
0
250
500
750
1000
1250
1500
1750
2000

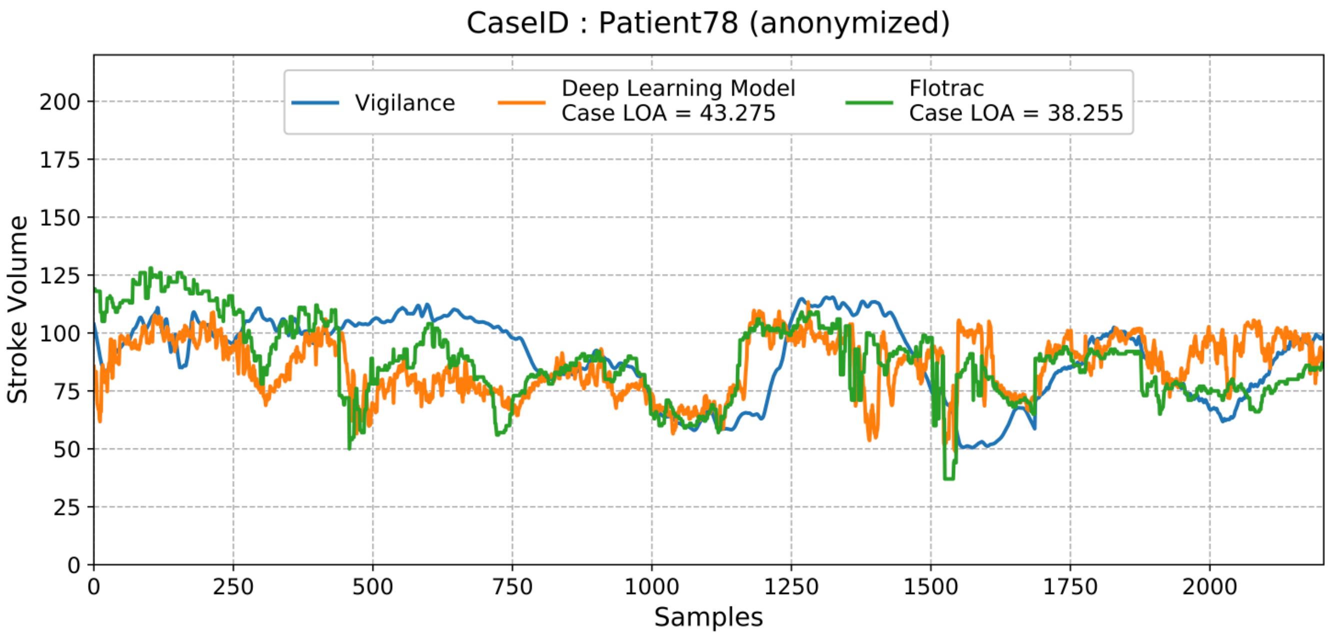

CaseID : Patient78 (anonymized)
Vigilance
Deep Learning Model
Case LOA = 43.275
Flotrac
Case LOA = 38.255
Stroke Volume
Samples
0
25
50
75
100
125
150
175
200
0
250
500
750
1000
1250
1500
1750
2000

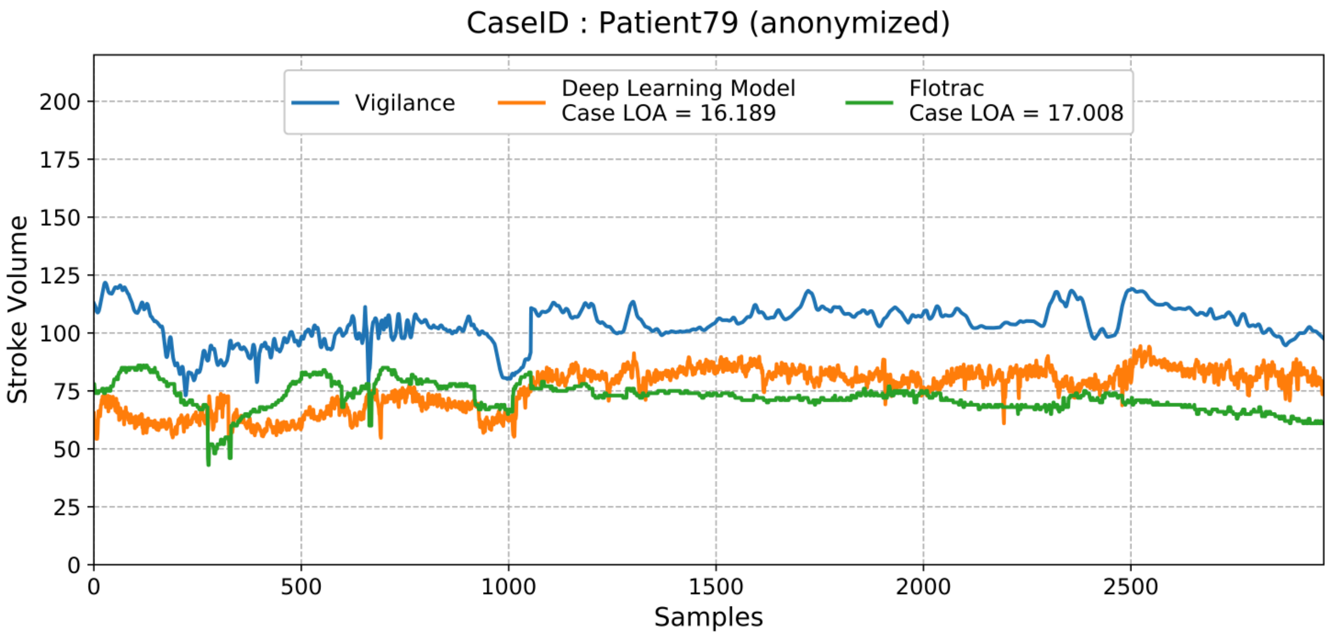

CaseID : Patient79 (anonymized)
Vigilance
Deep Learning Model
Case LOA = 16.189
Flotrac
Case LOA = 17.008
Stroke Volume
Samples
0
25
50
75
100
125
150
175
200
0
500
1000
1500
2000
2500

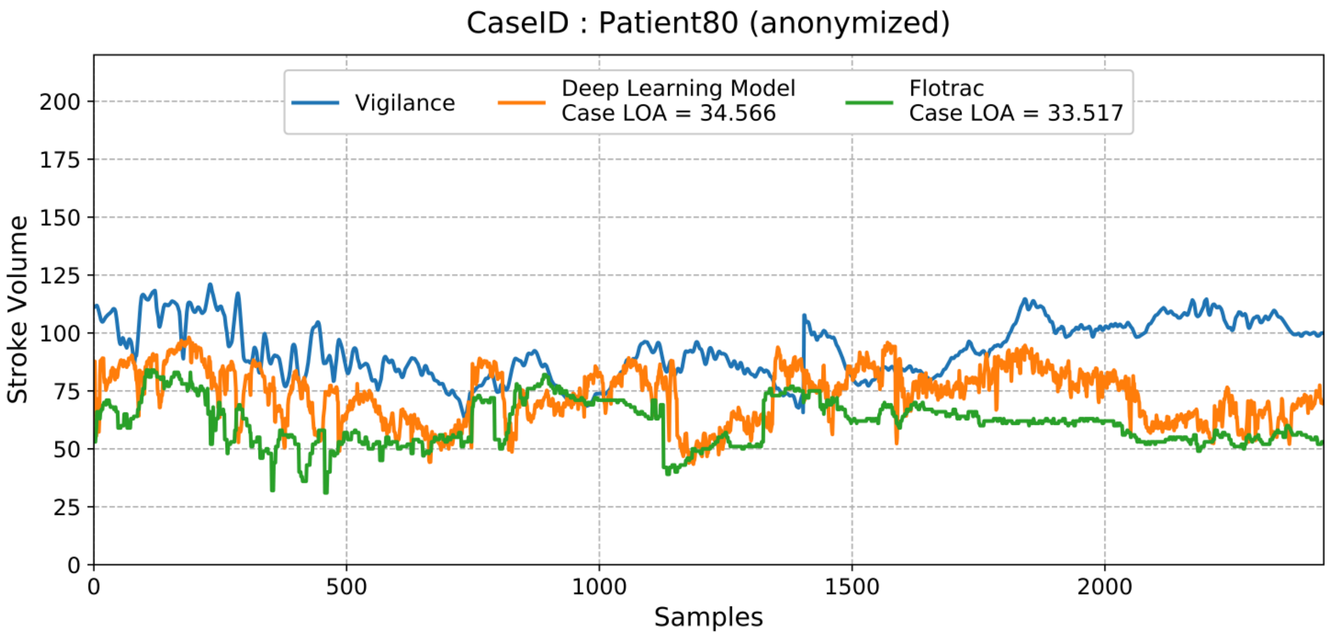

CaseID : Patient80 (anonymized)
Vigilance
Deep Learning Model
Case LOA = 34.566
Flotrac
Case LOA = 33.517
Stroke Volume
Samples
0
25
50
75
100
125
150
175
200
0
500
1000
1500
2000

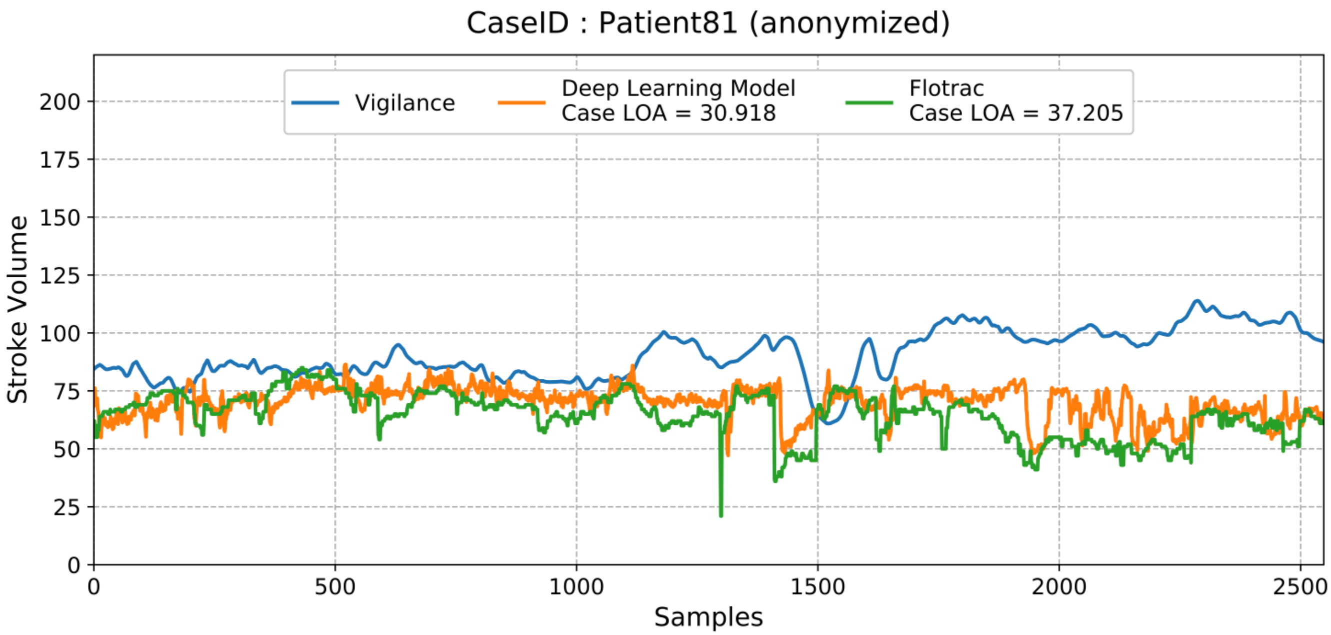
CaseID : Patient81 (anonymized)
Vigilance
Deep Learning Model
Case LOA = 30.918
Flotrac
Case LOA = 37.205
Stroke Volume
Samples
0
25
50
75
100
125
150
175
200
500
1000
1500
2000
2500

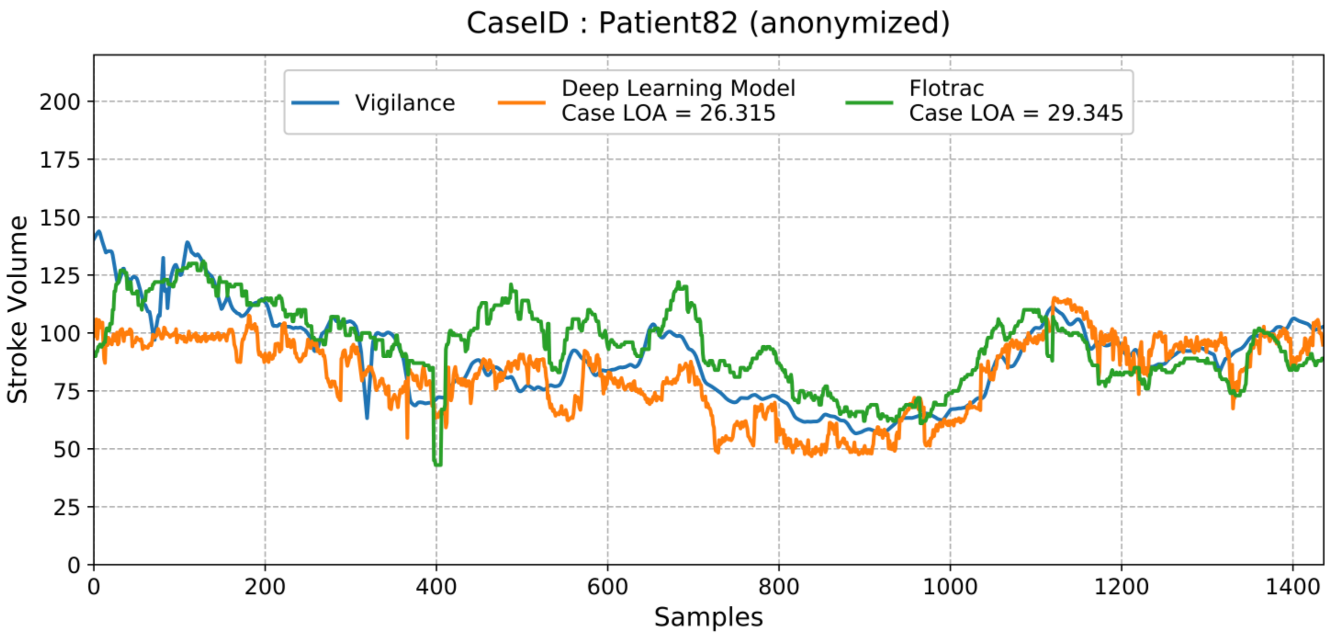
CaseID : Patient82 (anonymized)
Vigilance
Deep Learning Model
Case LOA = 26.315
Flotrac
Case LOA = 29.345
Stroke Volume
Samples
0
25
50
75
100
125
150
175
200
0
200
400
600
800
1000
1200
1400

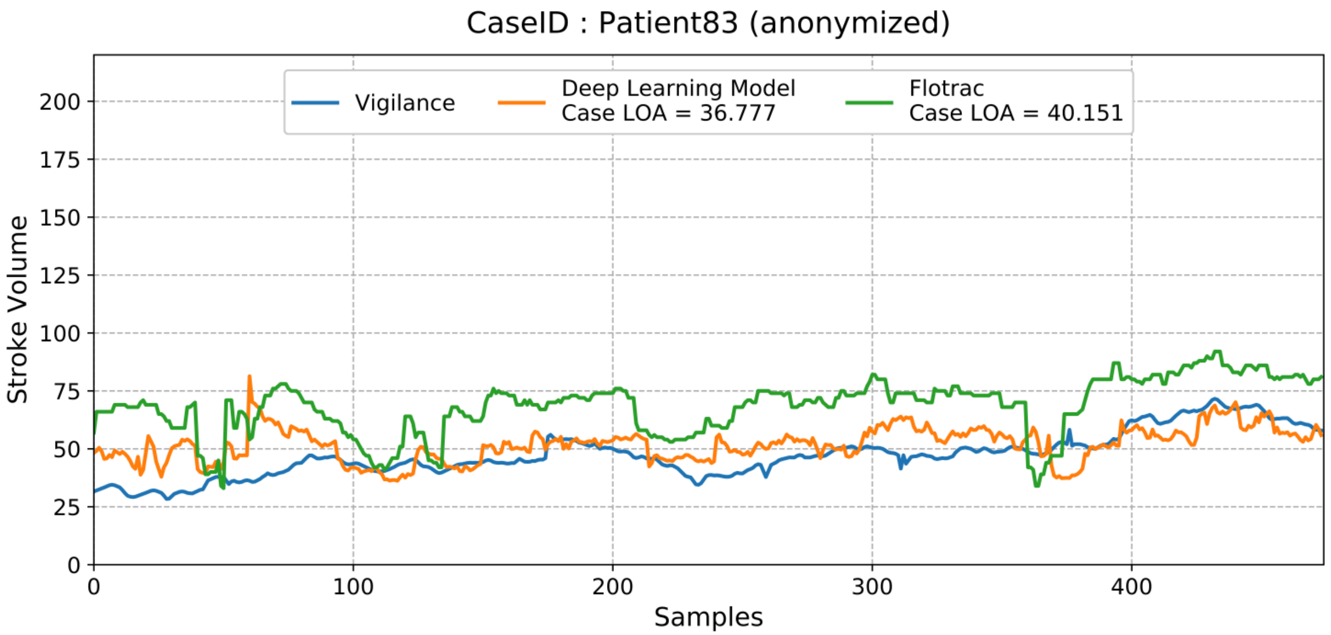

CaseID : Patient83 (anonymized)
Vigilance
Deep Learning Model
Case LOA = 36.777
Flotrac
Case LOA = 40.151
Stroke Volume
Samples
0
25
50
75
100
125
150
175
200
0
100
200
300
400

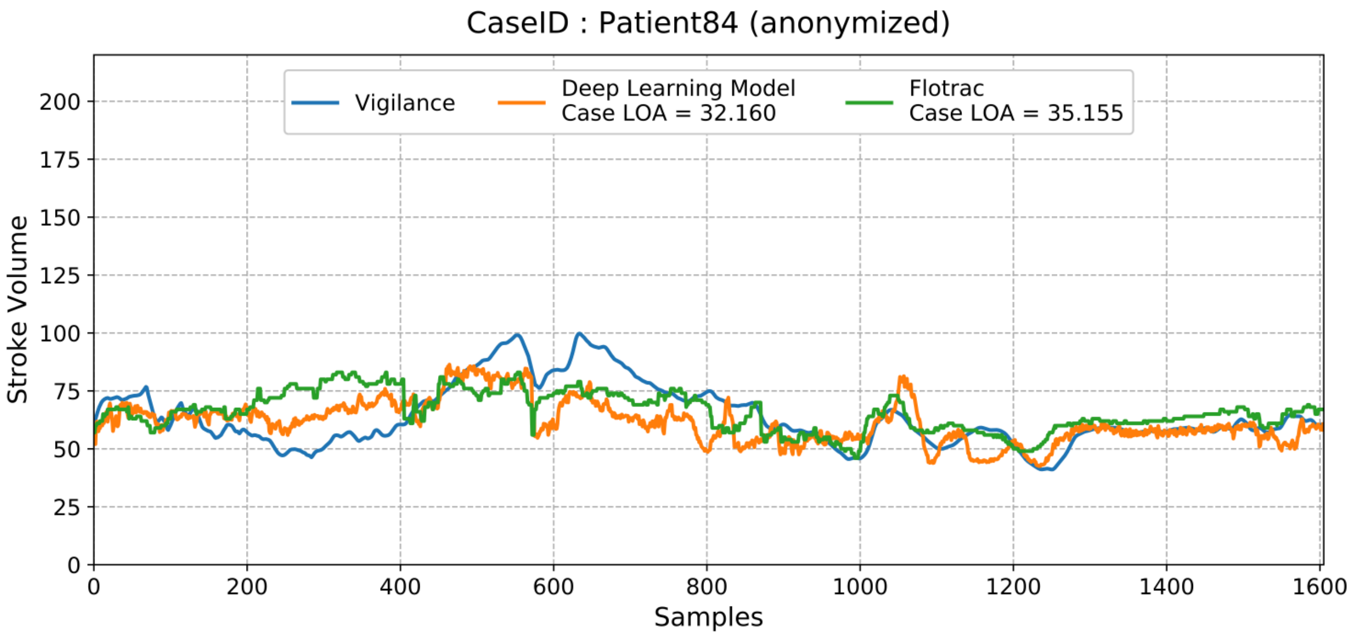
CaseID : Patient84 (anonymized)
Vigilance
Deep Learning Model
Case LOA = 32.160
Flotrac
Case LOA = 35.155
Stroke Volume
Samples
0
25
50
75
100
125
150
175
200
0
200
400
600
800
1000
1200
1400
1600

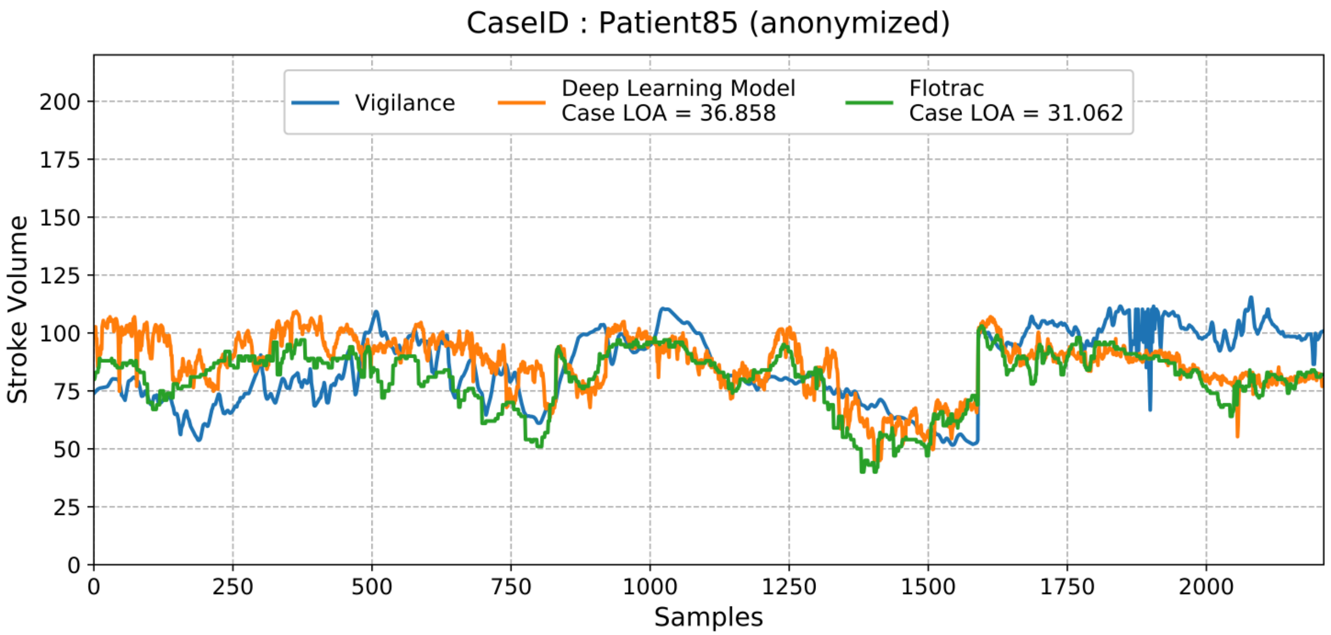
CaseID : Patient85 (anonymized)
Vigilance
Deep Learning Model
Case LOA = 36.858
Flotrac
Case LOA = 31.062
Stroke Volume
0
25
50
75
100
125
150
175
200
Samples
0
250
500
750
1000
1250
1500
1750
2000

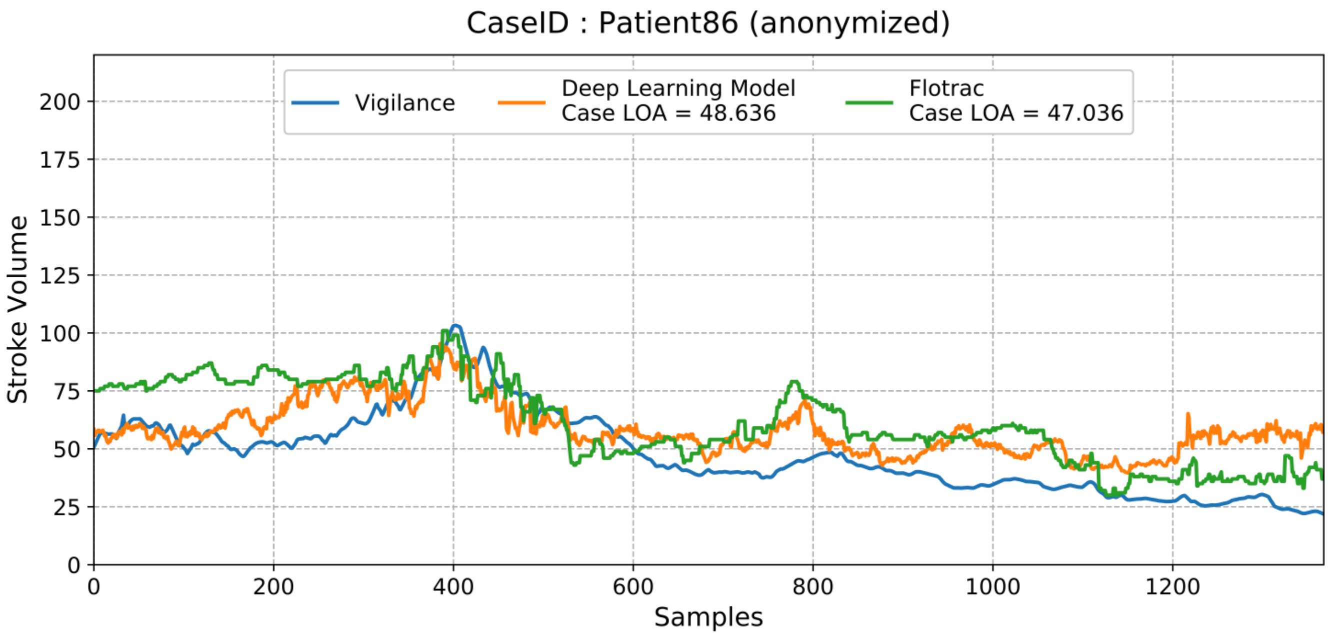

CaseID : Patient86 (anonymized)
Vigilance
Deep Learning Model
Case LOA = 48.636
Flotrac
Case LOA = 47.036
Stroke Volume
Samples
0
25
50
75
100
125
150
175
200
0
200
400
600
800
1000
1200

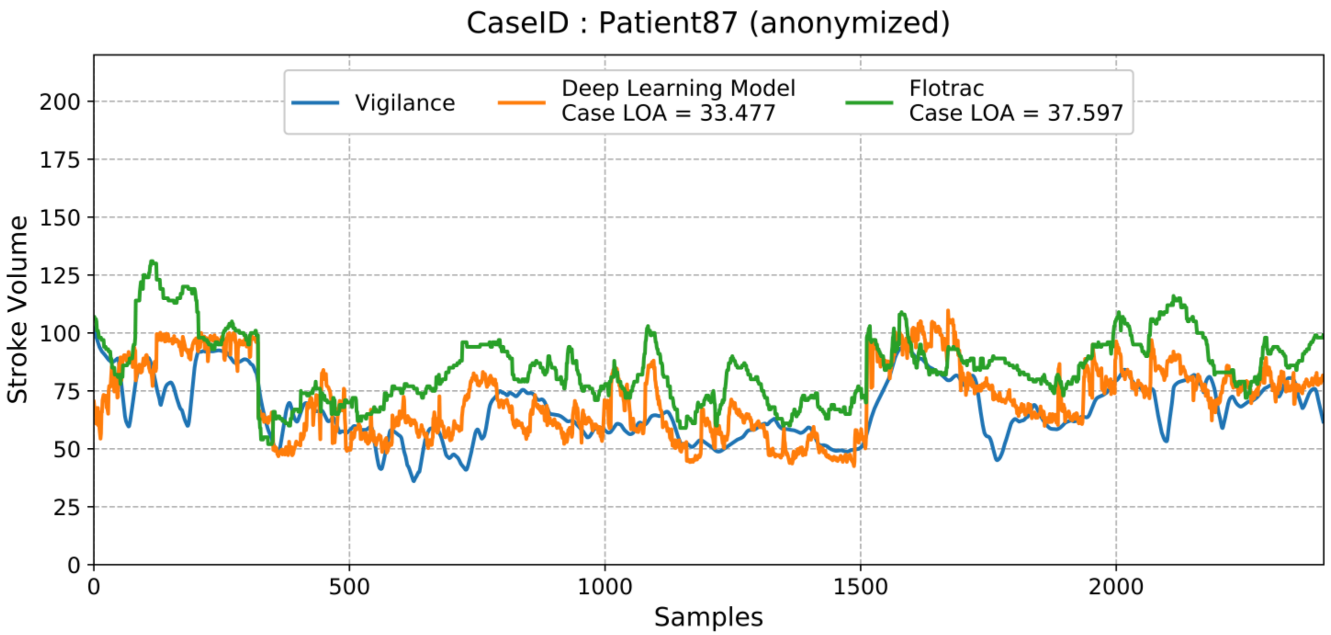
CaseID : Patient87 (anonymized)
Vigilance
Deep Learning Model
Case LOA = 33.477
Flotrac
Case LOA = 37.597
Stroke Volume
200
175
150
125
100
75
50
25
0
0
500
1000
1500
2000
Samples

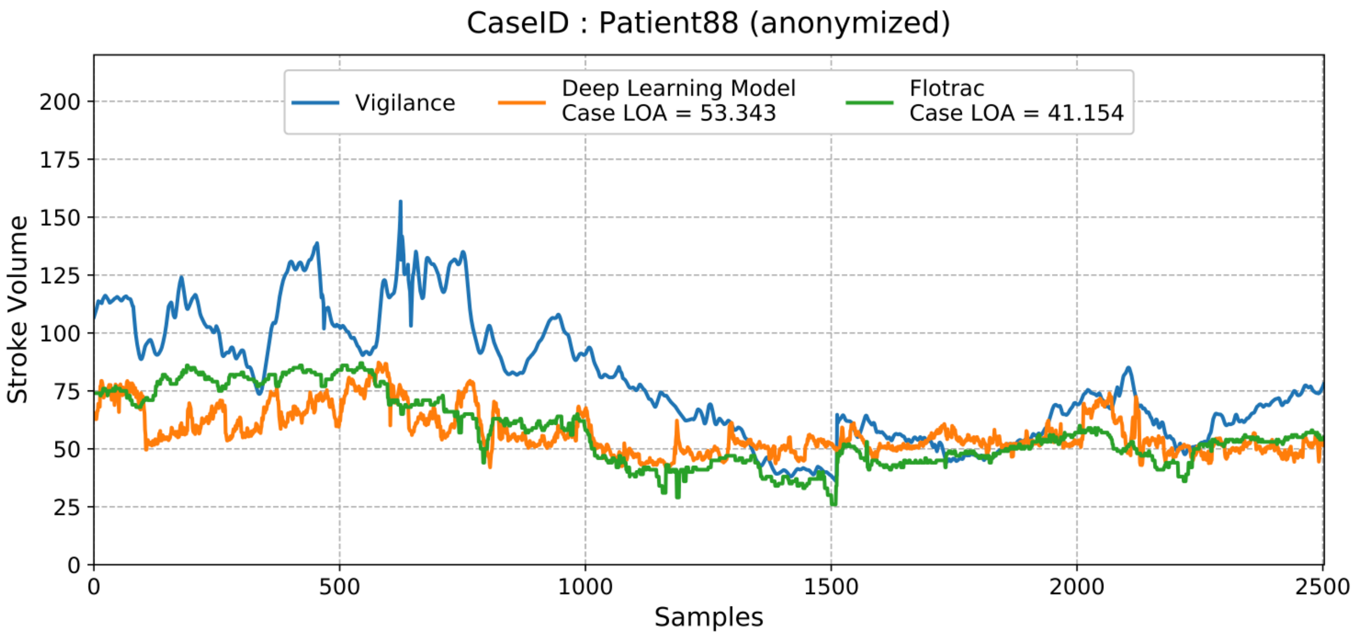

CaseID : Patient88 (anonymized)
Vigilance
Deep Learning Model
Case LOA = 53.343
Flotrac
Case LOA = 41.154
Stroke Volume
Samples
0
25
50
75
100
125
150
175
200
0
500
1000
1500
2000
2500

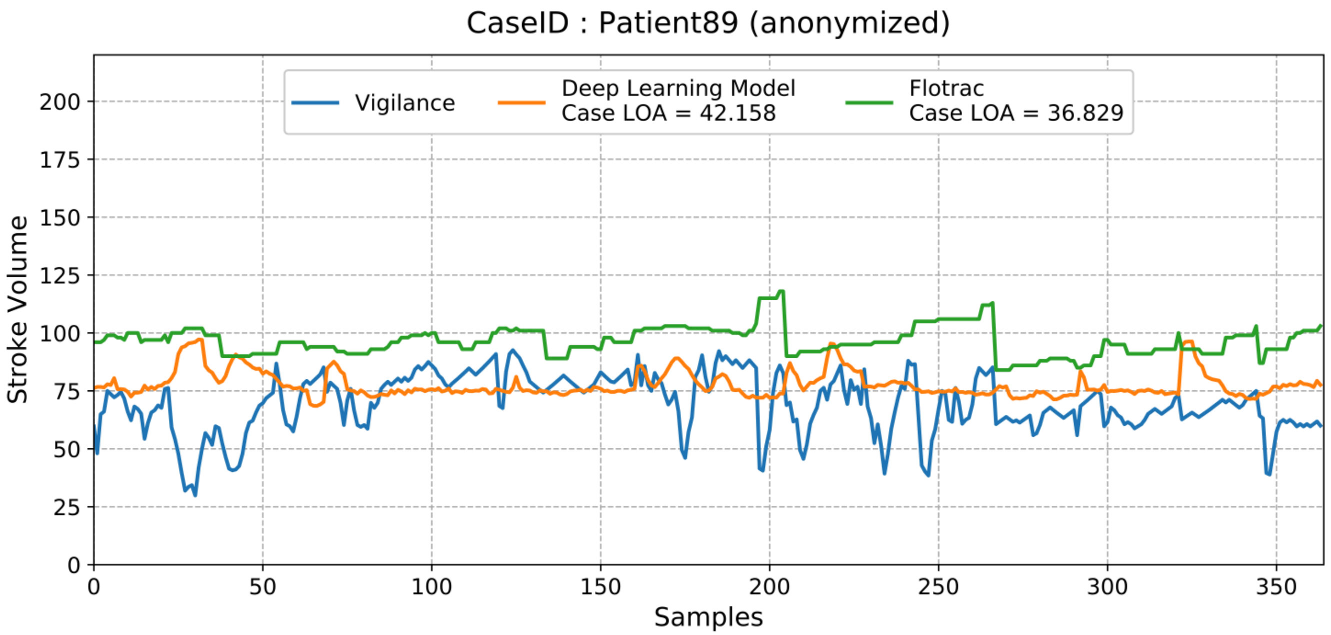

CaseID : Patient89 (anonymized)
Vigilance
Deep Learning Model
Case LOA = 42.158
Flotrac
Case LOA = 36.829
Stroke Volume
Samples
0
25
50
75
100
125
150
175
200
0
50
100
150
200
250
300
350

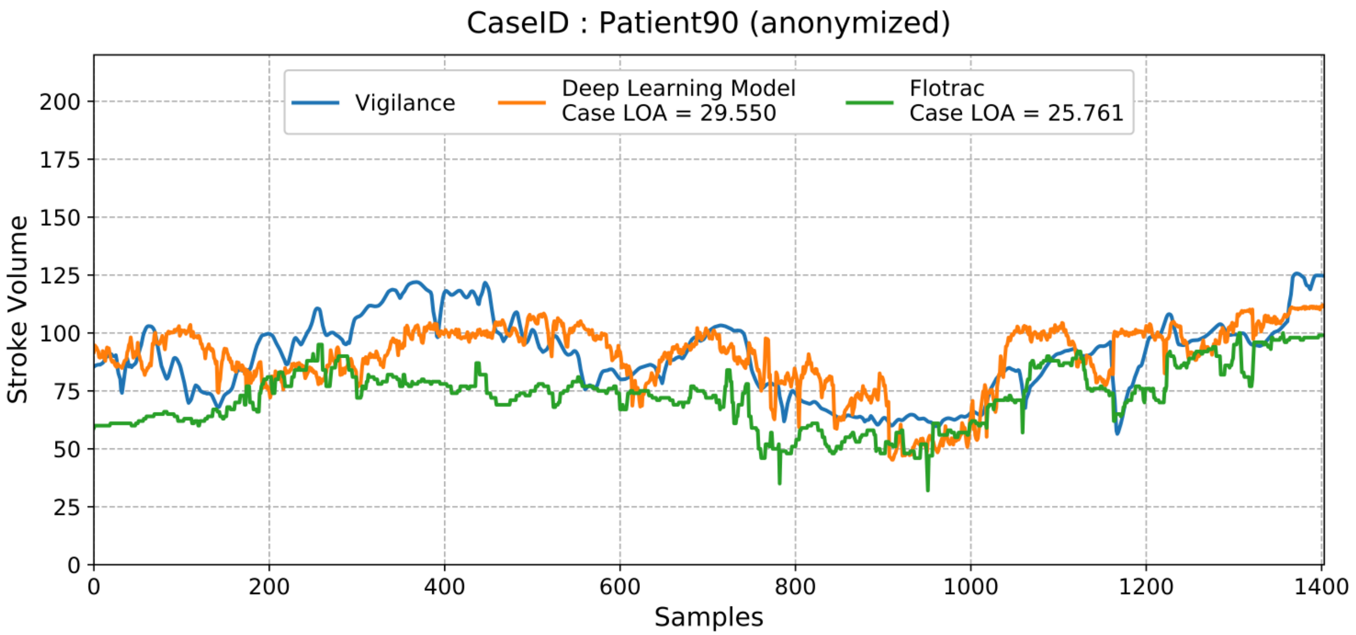
CaseID : Patient90 (anonymized)
Vigilance
Deep Learning Model
Case LOA = 29.550
Flotrac
Case LOA = 25.761
Stroke Volume
Samples
0
25
50
75
100
125
150
175
200
0
200
400
600
800
1000
1200
1400

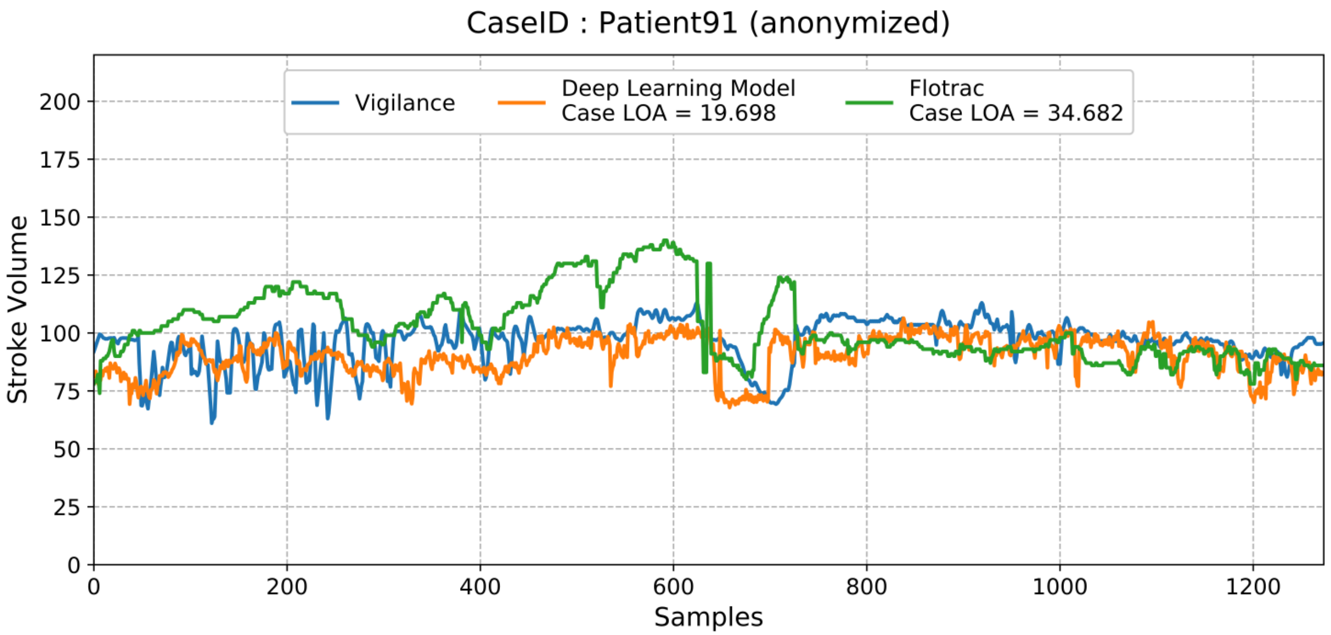
CaseID : Patient91 (anonymized)
Vigilance
Deep Learning Model
Case LOA = 19.698
Flotrac
Case LOA = 34.682
Stroke Volume
Samples
0
25
50
75
100
125
150
175
200
0
200
400
600
800
1000
1200

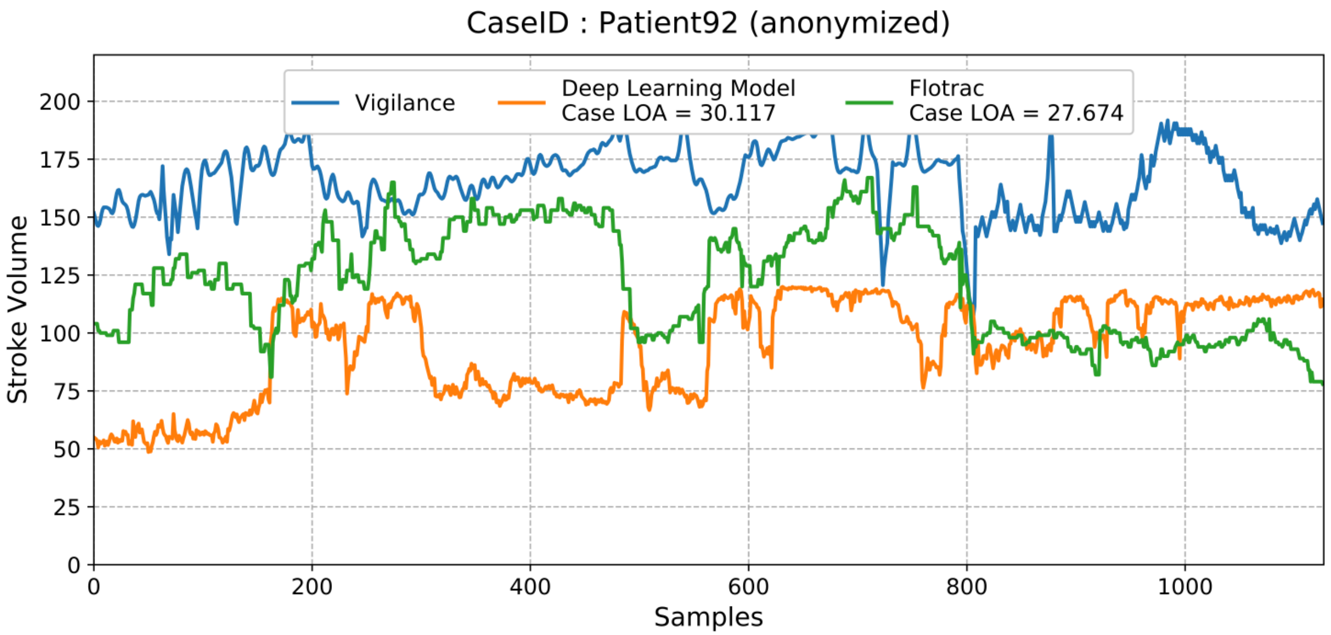

CaseID : Patient92 (anonymized)
Vigilance
Deep Learning Model
Case LOA = 30.117
Flotrac
Case LOA = 27.674
Stroke Volume
Samples
0
25
50
75
100
125
150
175
200
0
200
400
600
800
1000

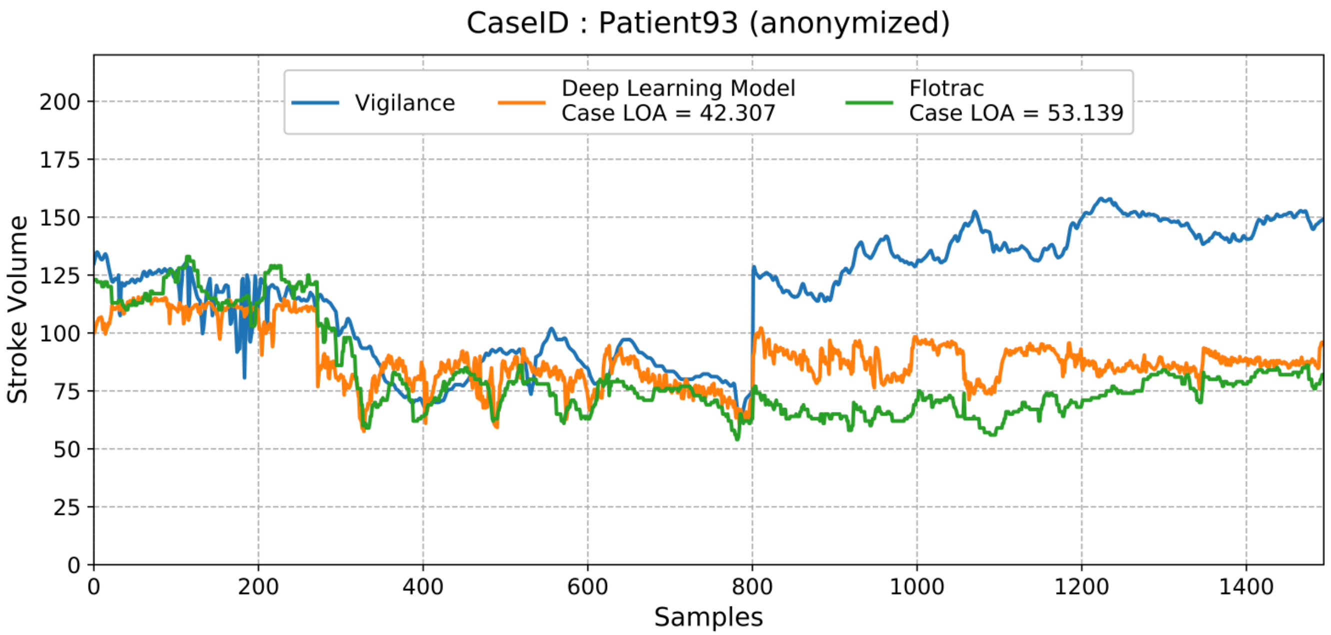
CaseID : Patient93 (anonymized)
Vigilance
Deep Learning Model
Case LOA = 42.307
Flotrac
Case LOA = 53.139
Stroke Volume
Samples
0
25
50
75
100
125
150
175
200
0
200
400
600
800
1000
1200
1400

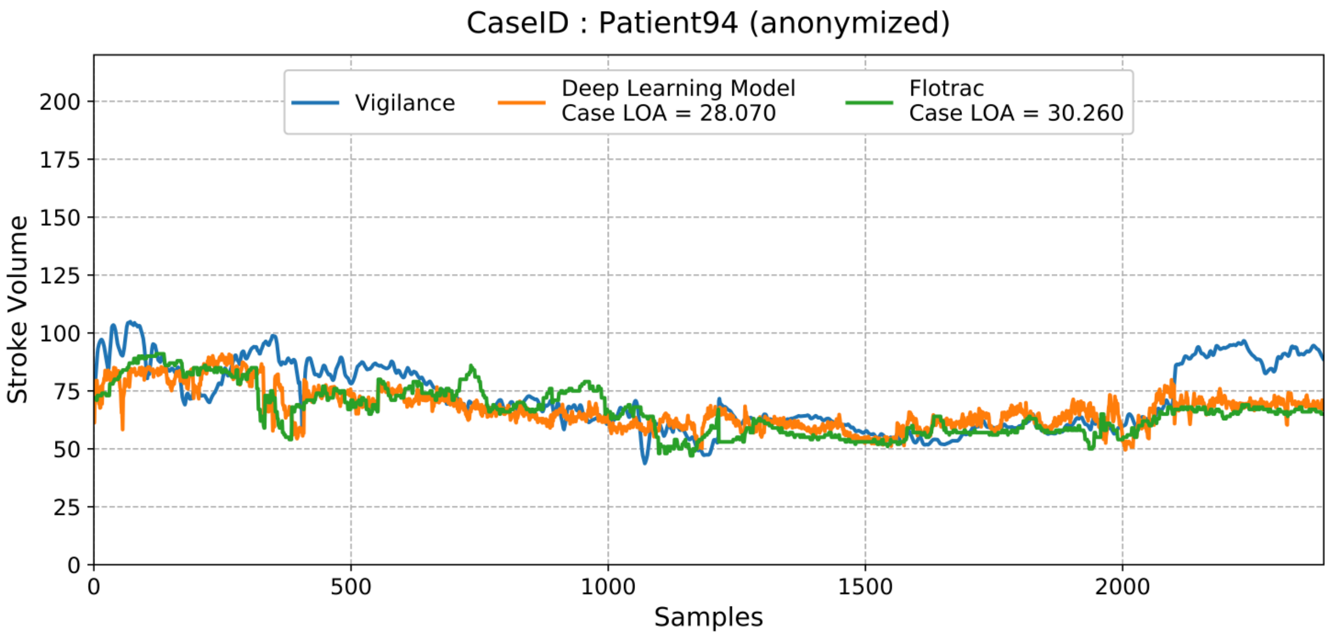
CaseID : Patient94 (anonymized)
Vigilance
Deep Learning Model
Case LOA = 28.070
Flotrac
Case LOA = 30.260
Stroke Volume
Samples
0
25
50
75
100
125
150
175
200
500
1000
1500
2000

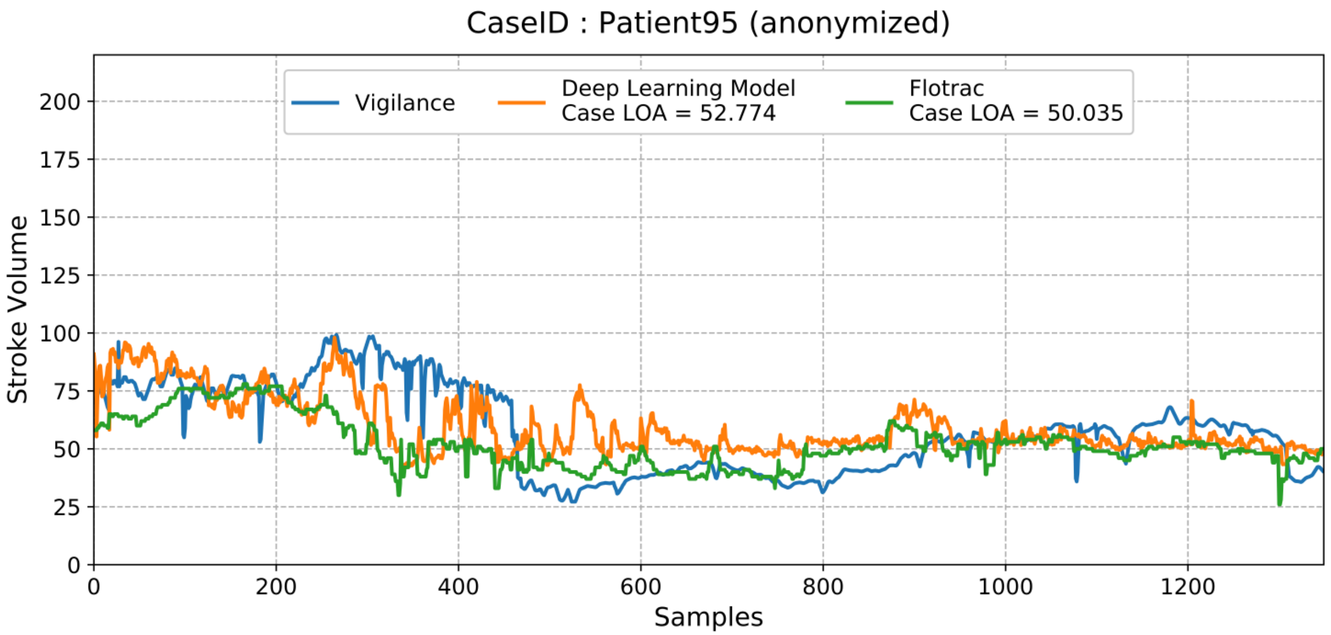

CaseID : Patient95 (anonymized)
Vigilance
Deep Learning Model
Case LOA = 52.774
Flotrac
Case LOA = 50.035
Stroke Volume
Samples
0
25
50
75
100
125
150
175
200
0
200
400
600
800
1000
1200

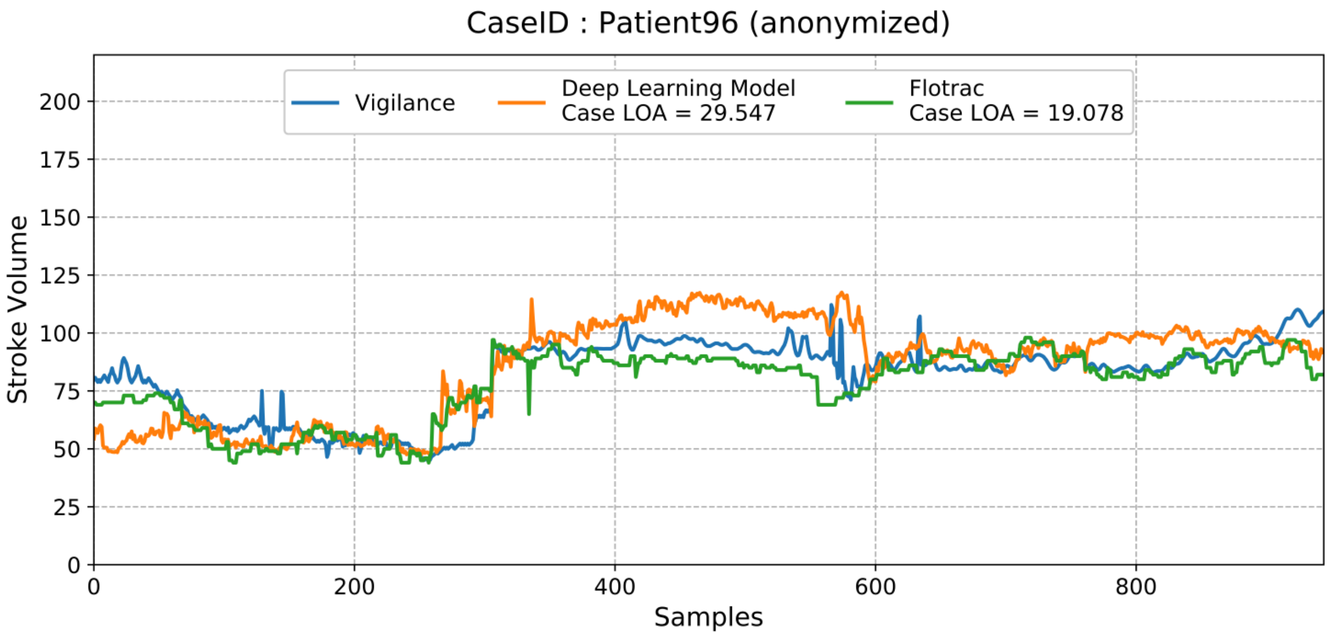

CaseID : Patient96 (anonymized)
Vigilance
Deep Learning Model
Case LOA = 29.547
Flotrac
Case LOA = 19.078
Stroke Volume
Samples
0
25
50
75
100
125
150
175
200
0
200
400
600
800

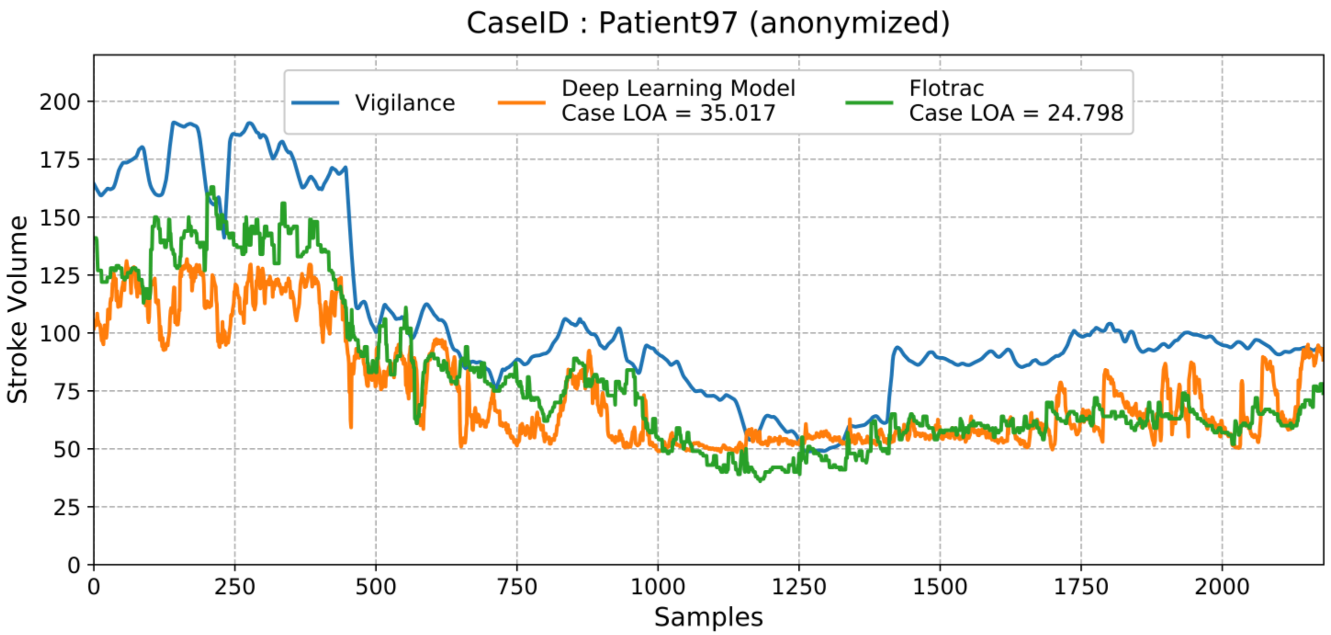

CaseID : Patient97 (anonymized)
Vigilance
Deep Learning Model
Case LOA = 35.017
Flotrac
Case LOA = 24.798
Stroke Volume
Samples
0
25
50
75
100
125
150
175
200
250
500
750
1000
1250
1500
1750
2000

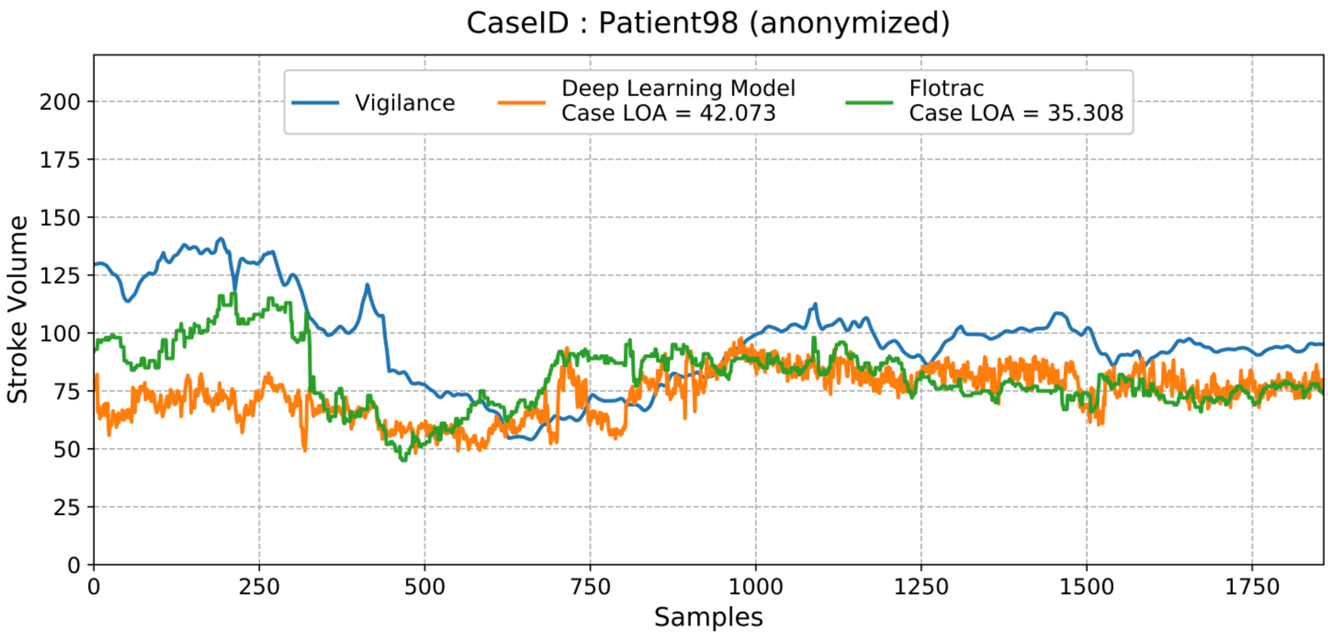

CaseID : Patient98 (anonymized)
Vigilance
Deep Learning Model
Case LOA = 42.073
Flotrac
Case LOA = 35.308
Stroke Volume
Samples
0
25
50
75
100
125
150
175
200
0
250
500
750
1000
1250
1500
1750
